# Statistical inference for heterogeneous treatment effect with right-censored data from synthesizing randomized clinical trials and real-world data

**DOI:** 10.1093/biomtc/ujaf131

**Published:** 2025-10-08

**Authors:** Guangcai Mao, Shu Yang, Xiaofei Wang

**Affiliations:** Department of Biostatistics and Bioinformatics, Duke University, Durham, NC 27710, United States; Department of Statistics, North Carolina State University, Raleigh, NC 27695, United States; Department of Biostatistics and Bioinformatics, Duke University, Durham, NC 27710, United States

**Keywords:** heterogeneous treatment effect, inverse probability weighting, nonparametric penalized estimation, sieve approximation, time-to-event endpoint

## Abstract

The heterogeneous treatment effect plays a crucial role in precision medicine. There is evidence that real-world data, even subject to biases, can be employed as supplementary evidence for randomized clinical trials to improve the statistical efficiency of the heterogeneous treatment effect estimation. In this paper, for survival data with right censoring, we consider estimating the heterogeneous treatment effect, defined as the difference of the treatment-specific conditional restricted mean survival times given covariates, by synthesizing evidence from randomized clinical trials and the real-world data with possible biases. We define an omnibus bias function to characterize the effect of biases caused by unmeasured confounders, censoring, and outcome heterogeneity, and further, identify it by combining the trial and real-world data. We propose a penalized sieve method to estimate the heterogeneous treatment effect and the bias function. We further study the theoretical properties of the proposed integrative estimators based on the theory of reproducing kernel Hilbert space and empirical process. The proposed methodology outperforms the approach solely based on the trial data through simulation studies and an integrative analysis of the data from a randomized trial and a real-world registry on early-stage non-small-cell lung cancer.

## 1 INTRODUCTION

The average treatment effect is commonly used to assess treatment effects, but it may not account for differences among individuals. Precision medicine, considering individual characteristics, has spurred interest in heterogeneous treatment effect (HTE). Randomized clinical trials (RCTs) are reliable but limited in sample diversity and external validity. Real-world data (RWD) can provide valuable complementary information but may be biased. Addressing these biases and integrating the RWD with the RCT data enhances study efficiency and accuracy.

The integration of RCTs with the RWD is becoming increasingly common, particularly in oncology and rare-disease settings, where registry and electronic health record (EHR) systems can be readily linked to Phase III trials (FDA, 2021; Carrigan et al., 2022). For example, several cancer RCTs have been successfully augmented with National Cancer Database data (Lee et al., 2024b) and with Flatiron Health EHR data to bolster subgroup analyses in older adults and smaller tumor-size strata (Ye et al., 2021). Other disease areas, such as diabetes, have likewise benefited from RCT–EHR integration (Kurki et al., 2024). Significant attention in the literature has been drawn to the integrative analysis method of the RCT data and the RWD. Prentice et al. (2008) introduced joint analysis for pooled data, while Soares et al. (2014) developed a hierarchical Bayesian model based on network meta-analysis. Efthimiou et al. (2017) compared various approaches, including naive data synthesis, design-adjust synthesis, and a three-level hierarchical model using the RWD as prior information. Verde and Ohmann (2015) summarized meta-analysis methods comprehensively, and Wang and Rosner (2019) extended the propensity score adjustment to a multi-study setting, proposing a Bayesian nonparametric Dirichlet process mixture model. Lee et al. (2023, 2024b) introduced an integrative estimator of average treatment effect, and Lee et al. (2022) and Lee et al. (2024a) proposed doubly robust estimators for generalizing average treatment effects on survival outcomes from trial data to a target population. These methods, however, often assume no biases in the RWD, which is unlikely. Methods addressing biases include preliminary testing (Yang et al., 2023), instrumental variable methods (Angrist et al., 1996), negative controls (Kuroki and Pearl, 2014), and sensitivity analysis (Robins et al., 1999). Yang et al. (2025) introduced a confounding function approach to handle unmeasured confounder bias, leveraging transportability and trial treatment randomization to improve the HTE estimator. Colnet et al. (2023) reviewed methods for combining the RCT data and the RWD for non-survival outcomes.

For censored survival data, the restricted mean survival time is an easily interpretable, a clinically meaningful summary of survival function, which is defined as the area under the survival curve up to a pre-specified time. A common way to measure the HTE is to define it as the difference in the conditional restricted mean survival time (CRMST) between the treatment and control groups. The existing estimations for the CRMST can be roughly divided into 2 classes. One uses survival models for hazard rate. For example, Zucker (1998), Chen and Tsiatis (2001), and Zhang and Schaubel (2011) used the Cox proportional hazards model (Cox, 1972) for hazard rate, deriving conditional survival function estimates from the relationship between hazard rate and survival function. Another approach is to model the CRMST directly. For example, Tian et al. (2014) used a generalized linear regression model and estimated parameters through an inverse probability censoring weighted function, relying on the assumption that censoring time is independent of covariates. Wang and Schaubel (2018) relaxed such an assumption and estimated the survival function of censoring time by the Cox model and further constructed the estimating equation.

This paper considers statistical inference for the HTE with right-censored survival data by combining the RCT data and the RWD with possible biases. The HTE is defined as the difference in the CRMST between the treatment and control groups. Inspired by Wu and Yang (2022) and Yang et al. (2025), we define an omnibus bias function to summarize all sources of bias in the RWD. This results in a flexible and generalizable framework that can be applied across a wide range of study designs and data sources. As such, our approach is a valuable tool for researchers and practitioners working in various fields. Its capacity to handle biases in the RWD, without relying on unrealistic assumptions, makes it an essential tool for improving the accuracy and reliability of causal inference in real-world scenarios. In our methodology, the HTE and the bias function are modeled with fully nonparametric models and estimated by minimizing a proposed penalized loss function. To implement the proposed estimators, a sieve method is utilized to approximate the HTE and the bias function. Furthermore, we derive the convergence rates and local asymptotic normalities of the proposed estimators by reproducing kernel Hilbert space and empirical process theory. Simulation studies demonstrate the excellent performance of the proposed method, and an illustrative application of the method to the real data reveals some intriguing findings.

The remainder of the paper is structured as follows: Section 2 introduces some preliminaries, such as notations and definitions of the HTE and bias function. Section 3 introduces the penalized loss function and sieve method for estimating the unknown functional parameters. Section 4 presents the asymptotic properties of the proposed integrative estimators. Section 5 includes simulation studies and an application to a real non-small-cell lung cancer dataset for finite sample performance evaluation of the proposed approach. Finally, in Section 6, we present some discussions, and Web Appendix E in the supplementary material, including the proofs of theoretical results, is also provided.

## 2 PRELIMINARIES

### 2.1 Notations: HTE and data structure

For 2 positive sequences $a_n$ and $b_n$, $a_n \asymp b_n$ means $\lim _{n\rightarrow \infty }a_n / b_n = c$ for some constant $c > 0$. For real numbers *a* and $b$, let $a\wedge b = \min \lbrace a , b \rbrace$. Let *T* and *C* denote the failure and censoring times, respectively. Under right censoring, the observed variable is $(Y, \Delta )$, where $Y = T \wedge C$ is the observed time and $\Delta = I(T \le C )$ is the censoring indicator. Let $\boldsymbol{X} = (X_1, \ldots , X_p)^{\rm T}$ be the *p*-dimensional covariate and $A \in \lbrace 0, 1\rbrace$ be the binary treatment, where $A = 1$ and $A = 0$ indicate the active and the control treatments, respectively.

We consider 2 independent data sources, the RCT data, and the RWD. Let $S = 1$ denote the RCT participation and $S = 0$ denote the RWD participation. Therefore, the observed data structure for subject *i* can be concluded as $(Y_i, \Delta _i, \boldsymbol{X}_i, A_i, S_i)$. It is postulated that the data gathered from RCT comprises ${\cal V}_1 = \lbrace (Y_i, \Delta _i, \boldsymbol{X}_{i}, A_i, S_i = 1): i = 1,\ldots , n_1\rbrace$, with sample size $n_1$, which represents independent replications of $(Y, \Delta , \boldsymbol{X} , A , S = 1)$, while the data from RWD is represented by ${\cal V}_0 = \lbrace (Y_i, \Delta _i, \boldsymbol{X}_i, A_i, S_i = 0): i = n_1 + 1,\ldots , n_1+ n_0 \rbrace$ with a sample size of $n_0$, which are independent copies of $(Y, \Delta , \boldsymbol{X} , A , S = 0)$.

This paper treats the RCT as the target population and utilizes potential outcomes (Neyman, 1923; Rubin, 1974) as the framework to define causal effects. Specifically, let $T(a)$ denote the potential outcome corresponding to the treatment $A = a$ for $a = 0 , 1$. We make the causal consistency assumption that $T = A T(1) + (1 - A) T(0)$. For a restricted time point *L*, the HTE is defined as


\begin{eqnarray*}
\tau (\boldsymbol{X}) = E \left\lbrace T(1)\wedge L - T(0)\wedge L \mid \boldsymbol{X}, S = 1 \right\rbrace .
\end{eqnarray*}

Our research goal is to estimate $\tau (\boldsymbol{X})$ based on the integrative dataset ${\cal V}_1 \cup {\cal V}_0$ containing $n = n_1 + n_0$ samples.

### 2.2 Identifiability of the HTE

We impose assumptions to explore the identiﬁability of the HTE from the observed data.

Assumption 1: (i) $0 < c_1 \le P(A = 1 \mid \boldsymbol{X} , S = 1 ) \le c_2 < 1$, where $c_1$ and $c_2$ are some constants; and (ii) $E\lbrace T(a)\wedge L \mid \boldsymbol{X} , A, S = 1\rbrace = E\lbrace T(a)\wedge L \mid \boldsymbol{X} , S = 1\rbrace$ for $a = 0 ,1$.

Assumption 2: (i) $T(a) \perp C \mid (\boldsymbol{X} , A, S = 1 )$ for $a = 0 , 1$; and (ii) $P(Y \ge L \mid \boldsymbol{X}, A , S = 1 ) > 0$.

Assumption 1(i) implies that each subject in the RCT has a positive probability of receiving treatment. It is also considered a fundamental assumption. Assumption 1(ii) is satisfied by default for the RCT. Of note, this assumption is formally weaker than the strong ignorability assumption on trial participation, that is, $T(a) \perp A \mid (\boldsymbol{X} , S = 1)$ for $a = 0 ,1$, which is a traditional assumption in causal inference. Assumption 2 is a standard assumption in survival analysis. Assumption 2(i) is also imposed in Zhang and Schaubel (2011). Assumption 2(ii) is a classical assumption in survival analysis and guarantees that the observed survival time can possess values within the vicinity of the restricted time point *L*. Particularly, these Assumptions are exclusively imposed on the RCT and not on the RWD, thereby extensively broadening the scope of the proposed methodology.

Let $T_L = A\lbrace T(1) \wedge L \rbrace + (1 - A)\lbrace T(0)\wedge L \rbrace$, $Y_L = Y \wedge L$, $e(\boldsymbol{X}) = P(A = 1 \mid \boldsymbol{X} , S = 1)$ be the treatment propensity score, and $\mu _a(\boldsymbol{X}) = E( T_L \mid \boldsymbol{X} , A = a, S = 1 )$ with $a = 0 ,1$. We now deliberate on the identifiability of the HTE from the RCT data. If the failure time *T* is precisely observed for all subjects, to identify the HTE, we can use the method proposed by Lee et al. (2017), in which an augmented inverse probability weighting (AIPW) approach is proposed for complete data. To be specific, let


\begin{eqnarray*}
R_1 &=& \frac{A T_L}{e(\boldsymbol{X})} - \frac{A - e(\boldsymbol{X})}{e(\boldsymbol{X})} \mu _1(\boldsymbol{X}), \\
R_0 &=& \frac{(1 - A) T_L}{1 - e(\boldsymbol{X})} + \frac{A - e(\boldsymbol{X})}{1- e(\boldsymbol{X})} \mu _0(\boldsymbol{X}), \quad R = R_1 - R_0.
\end{eqnarray*}

Under Assumption 1, Proposition S1 in Web Appendix C shows that $E( R \mid \boldsymbol{X} , S = 1 ) = \tau (\boldsymbol{X})$, which indicates the identifiability of $\tau (\cdot )$ from the RCT data in the uncensored case.

To handle the right censoring, an augmented inverse probability-of-censoring weighting (AIPCW) method (Zhao et al., 2015) is employed. Let $G_T(t \mid \boldsymbol{X} , A, S = 1) = P(T \ge t \mid \boldsymbol{X}, A , S = 1)$ and $G_C(t \mid \boldsymbol{X} , A, S = 1) = P(C \ge t \mid \boldsymbol{X}, A , S = 1)$ be the conditional survival functions of *T* and *C* given $\boldsymbol{X}$, *A* and $S = 1$, respectively. Let $\widetilde{\Delta } = I(T_L \le C)$, $N_C(t) = ( 1 - \widetilde{\Delta } ) I(Y_L \le t)$, $Q_C(t) = \int _0^t I(Y_L \ge u ) G^{-1}_C(u \mid \boldsymbol{X}, A, S = 1){\rm d}G_C(u \mid \boldsymbol{X}, A, S = 1)$, $M_C(t) = N_C(t) + Q_C(t)$, $B(t) = E(T_L \mid T_L > t , \boldsymbol{X}, A, S = 1 ) = t + { \int _t^L G_T(u \mid \boldsymbol{X}, A , S = 1 ){\rm d}u } / {G_T(t \mid \boldsymbol{X}, A , S = 1 )}$, and further


\begin{eqnarray*}
\widetilde{T}_L &=& {Y_L \widetilde{\Delta }}{G^{-1}_C( Y_L \mid \boldsymbol{X} , A, S = 1)} \\
&&- \int _0^L B(t)G^{-1}_C(t \mid \boldsymbol{X} , A, S = 1){\rm d}M_C(t).
\end{eqnarray*}

The first term of $\widetilde{T}_L$ is the familiar IPCW transformation, which is to convert the right-censored survival outcome into a complete outcome while preserving the conditional expectation of the outcome. The second term of $\widetilde{T}_L$ is the augmented component, which enhances the efficiency and robustness of the transformation provided by the first term. When implementing the transformation, one needs to use some survival models to estimate $G_C$ and $G_T$. Obviously, to ensure that the transformation is valid, the censoring time model must be correctly specified if there is no the second term. However, with the inclusion of the second half, either the failure time model or the censoring time model is correctly specified, not necessarily both. This is known as the double robustness property, which increases the tolerance for model misspecification.

Since $M_C(t)$ is a zero-mean martingale (Fleming and Harrington, 1991), the conditional mean of the second term of $\widetilde{T}_L$ is zero. Intuitively, we have $E( \widetilde{T}_L \mid \boldsymbol{X} , A , S = 1 ) = E( {T}_L \mid \boldsymbol{X} , A , S = 1 )$. The detailed theoretical proof is provided in Proposition S2 in Web Appendix C. Consequently, defining $\widetilde{R}$ by replacing $T_L$ with $\widetilde{T}_L$ in the definition of *R*, we obtain $E( \widetilde{R} \mid \boldsymbol{X} , S = 1 ) = \tau (\boldsymbol{X})$. We call $\widetilde{R}$ the pseudo-individual treatment effect (pseudo-ITE).

If $E(T(1)\wedge L \mid A = 1, \boldsymbol{X}, S = 0) - E(T(0)\wedge L \mid A = 0, \boldsymbol{X}, S = 0) = \tau (\boldsymbol{X})$ and all trial assumptions are met for the RWD, one can define the pseudo-ITE for the RWD in a similar manner to that for the RCT, denoted by $\widetilde{R}^{*}$. In such a case, we have $E(\widetilde{R}^{*} \mid \boldsymbol{X}, S = 0 ) = \tau (\boldsymbol{X})$. Furthermore, let $D = S\, \widetilde{R} + (1 - S )\, \widetilde{R}^{*}$, then $E(D \mid \boldsymbol{X}, S) = \tau (\boldsymbol{X})$, which implies the identifiability of $\tau (\cdot )$ by combining the RCT data and the RWD. However, these assumptions might not be true for the RWD due to the various biases in the RWD. Thus, $\lambda (\boldsymbol{X}) = E( \widetilde{R}^{*} \mid \boldsymbol{X} , S = 0) - \tau (\boldsymbol{X}) = E( D \mid \boldsymbol{X} , S = 0) - \tau (\boldsymbol{X})$, referred to as the bias function, captures the impact of any assumption violation in the RWD. Therefore, we conclude that


(1) \begin{eqnarray*}
E\left( D \mid \boldsymbol{X} , S \right) = \tau (\boldsymbol{X}) + (1 - S) \lambda (\boldsymbol{X}),
\end{eqnarray*}

which implies the identifiability of $\tau (\cdot )$ and $\lambda (\cdot )$ from the integrative dataset ${\cal V}_1 \cup {\cal V}_0$. In fact, the choice of the pseudo-ITE for the RWD is not unique; it can be any reasonable proxy. In this paper, we opt to use $Y_L$ as a substitute for $\widetilde{R}^{*}$.

## 3 ESTIMATION METHODOLOGY

Let $\sigma ^2(\boldsymbol{x} , s) = \sigma ^2_s(\boldsymbol{x}) = {\rm Var}(D \mid \boldsymbol{X} = \boldsymbol{x}, S = s)$. Based on the integrative dataset ${\cal V}_1 \cup {\cal V}_0 = \lbrace (Y_i, \Delta _i, \boldsymbol{X}_i, A_i, S_i), i = 1,\ldots , n \rbrace$, equation (1) enables us to construct the loss function for $\tau (\cdot )$ and $\lambda (\cdot )$ as ${\ell }_n(\tau , \lambda ) = (2n)^{-1} \sum _{i = 1}^n \left\lbrace \sigma ^{2}(\boldsymbol{X}_i , S_i)\right\rbrace ^{-1} \left\lbrace {D}_i - \tau (\boldsymbol{X}_i) - (1 - S_i)\lambda (\boldsymbol{X}_i) \right\rbrace ^2$. Then, an estimated version of the loss function, $\widehat{\ell }_n(\tau , \lambda )$, can be written as $\widehat{\ell }_n(\tau , \lambda ) = (2n)^{-1} \sum _{i = 1}^n \left\lbrace \widehat{\sigma }^{2}(\boldsymbol{X}_i , S_i)\right\rbrace ^{-1}\big \lbrace \widehat{D}_i - \tau (\boldsymbol{X}_i) - (1 - S_i)\lambda (\boldsymbol{X}_i) \big \rbrace ^2$, where $\widehat{D} = S\, \widehat{R} + (1 - S)\, Y_L$, $\widehat{R}$ is defined by replacing ${e}(\boldsymbol{X})$, ${\mu }_a(\boldsymbol{X})$, ${G}_T(t \mid \boldsymbol{X} , A, S = 1)$, and ${G}_C(t \mid \boldsymbol{X} , A, S = 1)$ with the estimators $\widehat{e}(\boldsymbol{X})$, $\widehat{\mu }_a(\boldsymbol{X})$, $\widehat{G}_T(t \mid \boldsymbol{X} , A, S = 1)$, and $\widehat{G}_C(t \mid \boldsymbol{X} , A, S = 1)$ in the definition of $\widetilde{R}$, respectively, and $\widehat{\sigma }^2(\boldsymbol{X}, S)$ is the estimator of ${\rm Var}({D} \mid \boldsymbol{X}, S)$. In this paper, we propose the penalized loss function for $\tau (\cdot )$ and $\lambda (\cdot )$ as $\widehat{\ell }_{n, \gamma _1, \gamma _0}(\tau , \lambda ) = \widehat{\ell }_n(\tau , \lambda ) + {\gamma _1}/{2}J_1(\tau , \tau ) + {\gamma _0}/{2}J_0(\lambda , \lambda )$, where $\gamma _1$ and $\gamma _0$ are some positive penalized parameters and converge to zero as the sample size *n* goes to infinity, $J_1(\cdot , \cdot )$ and $J_0(\cdot , \cdot )$ are roughness penalties to avoid overfitting, encourage smoothness and information borrowing, and are defined in Web Appendix A.

Let $\tau _0(\cdot )$ and $\lambda _0(\cdot )$ be the true values of $\tau (\cdot )$ and $\lambda (\cdot )$. Throughout the paper, we assume that $\tau _0(\cdot )$ and $\lambda _0(\cdot )$ belong to the reproducing kernel Hilbert spaces ${\cal H}_1$ and ${\cal H}_0$, respectively, equipped with the norms $\Vert \cdot \Vert _{{\cal H}_1}$ and $\Vert \cdot \Vert _{{\cal H}_0}$. The spaces ${\cal H}_1$ and ${\cal H}_0$, as well as the norms $\Vert \cdot \Vert _{{\cal H}_1}$ and $\Vert \cdot \Vert _{{\cal H}_0}$, are defined in Web Appendix A. For inferring $\tau _0(\cdot )$ and $\lambda _0(\cdot )$, we use sieve expansion to approximate $\tau (\cdot )$ and $\lambda (\cdot )$ in $\widehat{\ell }_{n, \gamma _1, \gamma _0}(\tau , \lambda )$. Specifically, let $\lbrace \phi _1(\cdot ), \ldots , \phi _{r_1}(\cdot )\rbrace$ and $\lbrace \psi _1(\cdot ), \ldots , \psi _{r_0}(\cdot )\rbrace$ denote 2 sets of sieve basis functions, and let $\Phi _n$ and $\Psi _n$ be the corresponding spanned linear spaces, respectively, where $r_1$ and $r_0$ are the numbers of basis functions and represent the complexities of the approximations. Then, we propose the penalized nonparametric estimator of $(\tau _0, \lambda _0)$ as $(\widehat{\tau }_{n}, \widehat{\lambda }_{n}) = \arg {\min }_{(\tau ,\lambda ) \in \Phi _n \times \Psi _n}\widehat{\ell }_{n, \gamma _1, \gamma _0}(\tau , \lambda )$.

## 4 ASYMPTOTIC PROPERTIES

In this section, we delve into the asymptotic properties of the proposed estimators, focusing on consistency, convergence rates, and asymptotic normality.

Theorem 1: Assuming that Assumptions 1–2 are met, as well as Conditions S1-S6 as outlined in Web Appendix B. Then, $\Vert \widehat{\tau }_n - {\tau }_0 \Vert _{{\cal H}_1} + \Vert \widehat{\lambda }_{n} - \lambda _0 \Vert _{{\cal H}_0} = o_P(1)$.

Given that $\mu _a(\boldsymbol{X}) = E(T_L \mid \boldsymbol{X}, A = a, S = 1) = \int _0^L G_T(t \mid \boldsymbol{X}, A = a, S = 1 ){\rm d}t$, using $\widehat{\mu }_a(\boldsymbol{X}) = \int _0^L \widehat{G}_T(t \mid \boldsymbol{X}, A = a, S = 1 ){\rm d}t$ as an estimator for $\mu _a(\boldsymbol{X})$ is appropriate. In this context, Theorem 1 implies that if $\widehat{G}_T(t \mid \boldsymbol{X}, A , S = 1)$ is consistent, or both $\widehat{G}_C(t \mid \boldsymbol{X}, A , S = 1)$ and $\widehat{e}(\boldsymbol{X})$ are consistent, then $\widehat{\tau }_n(\cdot )$ and $\widehat{\lambda }_n(\cdot )$ are consistent estimators of $\tau _0(\cdot )$ and $\lambda _0(\cdot )$, respectively. For the RCT, where the probability of receiving treatment is typically known, Theorem 1 establishes that if either $\widehat{G}_T$ or $\widehat{G}_C$ is consistent, $\widehat{\tau }_n$ and $\widehat{\lambda }_n$ remain consistent. In such a case, Theorem 1 thus highlights the double robustness of the proposed HTE estimator, an important attribute that enhances its reliability. Alternatively, regardless of the knowledge of $e(\boldsymbol{X})$, one can use a nonparametric estimation approach for $\mu _a(\boldsymbol{X})$. Employing such methods ensures consistency, thereby providing reliable estimators $\widehat{\tau }_n$ and $\widehat{\lambda }_n$. Under stronger assumptions, Theorem 2 provides the convergence rates of the proposed estimators, making it a strengthened version of Theorem 1.

Theorem 2: Assuming that Assumptions 1–2 are met, as well as Conditions S1-S5, and S7-S9 as outlined in Web Appendix B. Then,

\begin{eqnarray*}
&& \Vert \widehat{\tau }_{n} - \tau _0 \Vert _{{\cal H}_1} + \Vert \widehat{\lambda }_{n} - \lambda _0 \Vert _{{\cal H}_0} = O_P(n^{-1/2}\gamma _1^{-p/(4m_1)} \\
&& \quad + n^{-1/2}\gamma _0^{-p/(4m_0)} + \gamma _1^{1/2} + \gamma _0^{1/2}).
\end{eqnarray*}

Remark 1: If $m_1 = m_0 = m$ and $\gamma _1 \asymp \gamma _0 \asymp n^{-2m/(2m+p)}$, then $\widehat{\tau }_{n}$ and $\widehat{\lambda }_n$ achieve the same optimal convergence rate of $O_P(n^{-m/(2m + p )})$, which is also the optimal rate in the most commonly used nonparametric methods.

Suppose that the covariate $\boldsymbol{X}$ takes values in an open connect set $\Omega$ with $C^{\infty }$ boundary. The pointwise asymptotic normality, which is crucial for constructing confidence intervals, is investigated in Theorem 3 under additional conditions.

Theorem 3: Assuming that the assumptions in Theorem 2 are met, as well as Conditions S10-S14 as outlined in Web Appendix B. Then, given $\boldsymbol{x}_0 \in \Omega$, we have

\begin{eqnarray*}
&& \left(n^{1/2}\gamma _1^{p/(4m_1)}\lbrace \widehat{\tau }_n(\boldsymbol{x}_0) - \tau ^{*}_0(\boldsymbol{x}_0) \rbrace , \right. \\
&& \quad \left. n^{1/2}\gamma _0^{p/(4m_0)}\lbrace \widehat{\lambda }_n(\boldsymbol{x}_0) - \lambda ^{*}_0(\boldsymbol{x}_0) \rbrace \right)^{\rm T} \rightsquigarrow N(0, \boldsymbol{\Sigma }),
\end{eqnarray*}

where $\rightsquigarrow$ denotes convergence in distribution, $\tau ^{*}_0$ and $\lambda ^{*}_0$ are the biased “true values,” and $\boldsymbol{\Sigma }$ is the covariance matrix. The definitions of $\tau ^{*}_0$, $\lambda ^{*}_0$, and $\boldsymbol{\Sigma }$ are provided in Web Appendix A.

Theorem 3 further indicates that the penalized components bias the proposed integrative estimators (see the definitions of $\tau ^{*}_0$ and $\lambda ^{*}_0$ in Web Appendix A). Analyzing the biases is challenging; however, the biases can be disregarded under specific undersmoothing conditions, as shown in Corollary 1.

Corollary 1: Suppose that the assumptions in Theorem 3 hold; furthermore, $n(\gamma _1 + \gamma _0) = O(1)$. Then, given $\boldsymbol{x}_0 \in \Omega$,

\begin{eqnarray*}
&& \left(n^{1/2}\gamma _1^{p/(4m_1)}\lbrace \widehat{\tau }_n(\boldsymbol{x}_0) - \tau _0(\boldsymbol{x}_0) \rbrace , \right.\\
&& \quad \left. n^{1/2}\gamma _0^{p/(4m_0)}\lbrace \widehat{\lambda }_n(\boldsymbol{x}_0) - \lambda _0(\boldsymbol{x}_0) \rbrace \right)^{\rm T} \rightsquigarrow N(0, \boldsymbol{\Sigma }).
\end{eqnarray*}

Let $\widehat{\tau }_{\rm rct}(\cdot )$ denote the estimator of $\tau _0(\cdot )$ derived solely from the trial data. The last aim is to theoretically show that the proposed integrative method is more efficient than using only the trial data, specifically ${\rm Var}\lbrace \widehat{\tau }_n(\boldsymbol{x}_0) \rbrace \le {\rm Var}\lbrace \widehat{\tau }_{\rm rct}(\boldsymbol{x}_0) \rbrace$. However, the estimators involve estimating nuisance functions, which makes calculating the variances very complex. For simplification, under some stronger conditions and treating nuisance functions as known, we show the proposed method has the advantage of gaining efficiency in estimating $\tau (\cdot )$, which is summarized in Theorem 4.

Theorem 4: Assuming that Assumptions 1–2 are met, as well as Conditions S1-S3, and S15-S17 as outlined in Web Appendix B. Then, given $\boldsymbol{x}_0 \in \Omega$, ${\rm Var} \lbrace \widehat{\tau }_n(\boldsymbol{x}_0)\rbrace \le {\rm Var} \lbrace \widehat{\tau }_{\rm rct}(\boldsymbol{x}_0)\rbrace $.

## 5 NUMERICAL STUDIES

### 5.1 Simulation study

In this section, we conduct some simulation studies to provide technical support for applying the proposed method. We consider various types of bias in the real-world study, including selection bias, censoring, outcome heterogeneity, and unmeasured confounding. We first consider the case where $p = 2$, that is $\boldsymbol{X} = (X_1, X_2)^{\rm T}$. Tensor B-splines are employed to get the sieve basis. In the simulation, $\widehat{G}_T$ and $\widehat{G}_C$ is obtained by fitting a Cox proportional hazards model (Cox, 1972), furthermore, $\widehat{\mu }_a(\boldsymbol{X}) = \int _0^L \widehat{G}_T(t \mid \boldsymbol{X}, A = a, S = 1){\rm d}t$, where $a = 0 , 1$. $\widehat{\sigma }^2_s(\boldsymbol{x})$ is obtained by a kernel estimation method and the corresponding bandwidth is twice the optimal bandwidth by the generalized cross-validation (GCV), where $s = 0 ,1$. The penalized parameters $\gamma _1$ and $\gamma _0$ are selected by GCV. For comparison, we also consider the estimators based solely on the RCT data and those based solely on the RWD.

Throughout the simulation, $e(\boldsymbol{X}) = P(A = 1 \mid \boldsymbol{X}, S = 1) = 0.5$ is known and $P(A = 1 \mid \boldsymbol{X}, S = 0 )$ is estimated via a generalized linear model. We consider three cases, each representing different scenarios. Case 1 corresponds to the situation where the failure time model is correctly specified, and $E(T_L \mid \boldsymbol{X}, A, S = 1) = E(T_L \mid \boldsymbol{X}, A, S = 0)$. Case 2 corresponds to the situation where the failure time model is correctly specified, but $E(T_L \mid \boldsymbol{X}, A, S = 1) \ne E(T_L \mid \boldsymbol{X}, A, S = 0)$. Case 3 addresses the situation where the failure time model is incorrectly specified, and $E(T_L \mid \boldsymbol{X}, A, S = 1) = E(T_L \mid \boldsymbol{X}, A, S = 0)$. The specific settings are summarized in Table 1.

**TABLE 1:** Simulation settings.

Restricted time point $L = 3$; Sample size $(n_1, n_0) = (500, 1000)$ and $(1000, 2000)$; Replication times = 1000			
Case	Parameter	RCT	RWD
Case 1	$X_1, X_2$	$\mathcal {N}(0, 1)$	$\mathcal {N}(0, 0.5)$
	$X_u$	${\rm Exp}(5)$	${\rm Exp}(5)$
	*A*	Bernoulli(0.5)	Bernoulli(${\rm expit}\lbrace 0.5(X_1 + X_2 - X_u + 1 )\rbrace$)
	*T*	$G_{T}(t \mid X_1, X_2, A , X_u ) = (1 + 0.02t)\exp \lbrace -0.1X_u t -0.2t \exp ( -0.2X_1 -0.5 X_2 + 0.4AX_1 + 1.3AX_2 )\rbrace $	
	*C*	$h_C(t \mid X_1, X_2) = h_{0C}(t) \exp ( 0.5 X_1 + 0.5 X_2 )$	
		$h_{0C}(t) = 1.47 \times 10^{-2}$	$h_{0C}(t) = 0.441$
	$\widetilde{L}$	4.9	4.5
	CR	40%	70%
Case 2	$X_1, X_2$	Same as in Case 1	
	$X_u$	$-$	$\mathcal {N}(0, 1)$
	*A*	Same as in Case 1	
	*T*	$h_{T}(t \mid X_1, X_2, A ) =$	
		$0.2 \exp ( -0.2X_1 -0.5 X_2 + 0.4AX_1 + 1.3AX_2 )$	$0.2 \exp ( -0.2X_1 -0.5 X_2 + 0.4AX_1 + 1.3AX_2 + X_u )$
	*C*	$h_C(t \mid X_1, X_2) = h_{0C}(t) \exp ( 0.5 X_1 + 0.5 X_2 )$	
		$h_{0C}(t) = 1.84 \times 10^{-2}$	$h_{0C}(t) = 0.552$
	$\widetilde{L}$	5	4.5
	CR	Same as in Case 1	
Case 3	$X_1, X_2$	Same as in Case 1	
	$X_u$	$\mathcal {N}(0, 1)$	$\mathcal {N}(0, 1)$
	*A*	Same as in Case 1	
	*T*	$h_{T}(t \mid X_1, X_2, A ) = 0.2 \exp ( -0.2X_1 -0.5 X_2 + 0.4AX_1 + 1.3AX_2 + X_u )$	
	*C*	$h_C(t \mid X_1, X_2) = h_{0C}(t) \exp ( 0.5 X_1 + 0.5 X_2 )$	
		$h_{0C}(t) = 1.47 \times 10^{-2}$	$h_{0C}(t) = 0.552$
	$\widetilde{L}$	5.2	4.5
	CR	Same as in Case 1	

$X_1, X_2$ : Baseline covariates; $X_u$: Unmeasured confounder in the RWD; *A*: Treatment assignment; *T*: Failure time; *C*: Censoring time; $\widetilde{L}$: Study duration; CR: Censoring rate; $\mathcal {N}$: Normal distribution; Exp: Exponential distribution; expit: ${\rm expit}(x) = \exp (x) / \exp (1 + x)$; $G_T$: Survival function of *T*; $h_T$: Hazard function of *T*; $h_C$: Hazard function of *C*; $h_{0C}$: Baseline hazard function of *C*.

**FIGURE 1:**
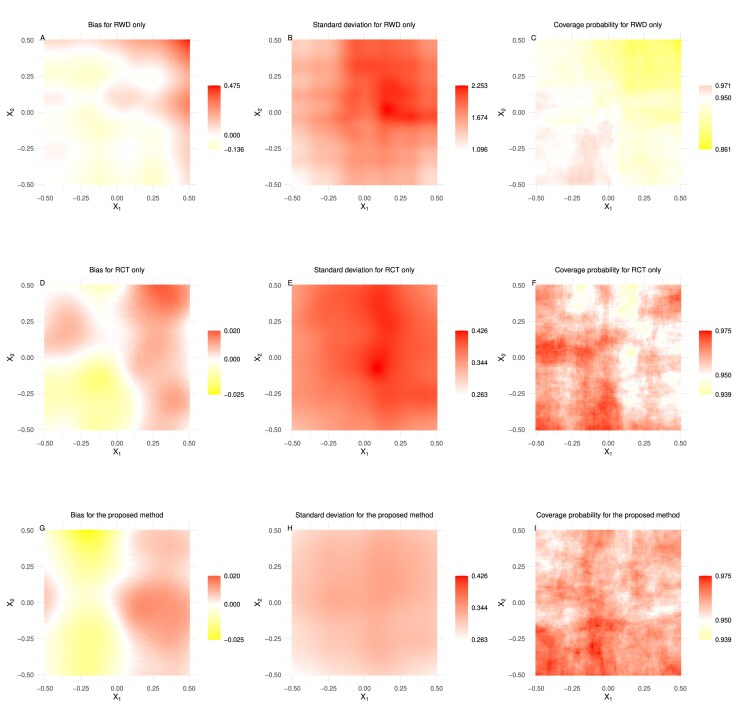
The simulation results of Case 1 with $(n_1, n_0) = (500, 1000)$.

**FIGURE 2:**
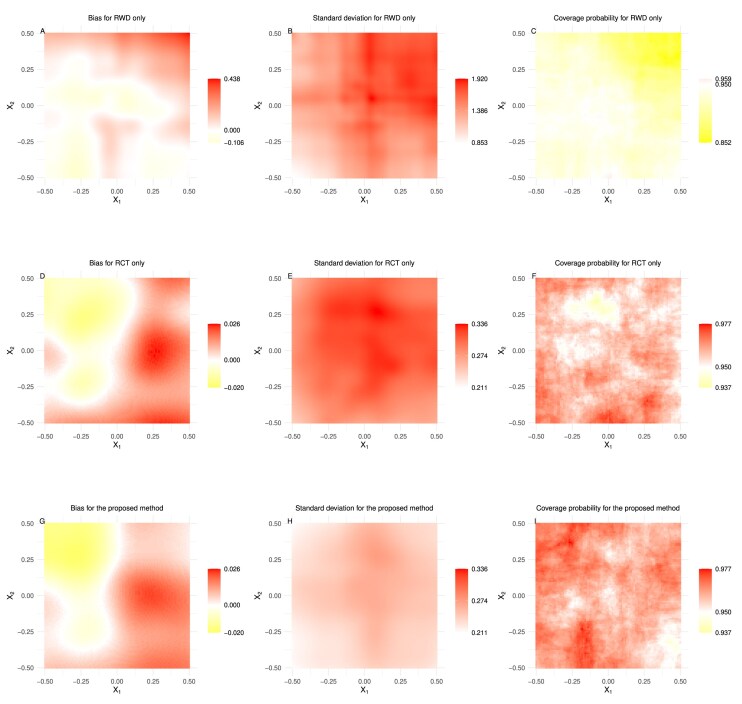
The simulation results of Case 1 with $(n_1, n_0) = (1000, 2000)$.

The simulation results are summarized in Figures 1–2 and S1-S4 in Web Appendix F. When presenting results graphically, we use different scales for the legends to ensure visibility and interpretability. The RWD-based only method has a distinct scale due to its significantly larger bias and standard deviation values. Using the same scale for all methods would make the RCT-based only and proposed methods indistinguishable. Thus, we assign a separate scale to the RWD-based only method and use a shared scale for the RCT-based only and proposed integrative methods to highlight the superiority of the proposed method.

Our expectations are met. The estimator based solely on the RWD performs poorly. Even though its performance improves with increasing sample size, the results remain poor, characterized by large biases, standard deviations, and unfavorable coverage probabilities. Both the proposed estimator and the estimator based solely on the RCT data perform well in estimation accuracy and coverage probability, closely aligning with the nominal values. The empirical standard deviations of both the proposed estimator and the estimator based solely on the RCT data decrease when the sample size $(n_1, n_0)$ is increased from $(500, 1000)$ to $(1000, 2000)$. Notably, in all configurations, the empirical standard deviations of the proposed estimators are smaller than those obtained solely from the RCT data. Considering estimation accuracy, coverage probability, and standard deviation together, it is evident that while both methods perform well in accuracy and coverage probability, the proposed method stands out due to its lower standard deviation. The reduced variability of the proposed estimator enhances its reliability, making it a superior choice for estimating the HTE in real-world scenarios. Overall, these findings confirm that the proposed integrative method performs well in finite-sample settings and significantly improves the HTE estimation compared to using the RCT data solely.

We also consider the case where $p = 4$, which includes 2 continuous covariates and 2 categorical covariates. The details are presented in Web Appendix F.

### 5.2 Real data application

Due to advances in radiologic technology, the detection rate of early-stage non-small-cell lung cancer is rising. The research community on the treatment of early-stage lung cancer with $\le$ 2 cm tumor had a great interest in evaluating the effect of limited resection relative to lobectomy. Lobectomy is a popular surgical resection in which the entire lobe of the lung where the tumor resides is removed. Limited resection, including wedge and segmental resection, only removes a smaller section of the complicated lobe. Limited resection is known for shorter hospital stays, fewer postoperative complications, and better preservation of pulmonary function. CALGB 140503 is a multicenter non-inferiority randomized phase 3 trial in which 697 patients with non-small-cell lung cancer clinically staged as stage 1A with $\le$ 2 cm tumors were randomly assigned to undergo limited resection or lobectomy (Altorki et al., 2023). The results of this trial firmly established that for stage 1A non-small-cell lung cancer patients with a tumor size of 2 cm or less, limited resection was not inferior to lobectomy concerning overall survival with a hazard ratio = 0.95 (90% confidence interval 0.72-1.26) and disease-free survival with a hazard ratio = 1.01 (90% confidence interval 0.83-1.24). Further subgroup analysis reveals that patients older than 70 years tended to have more prolonged disease-free survival and overall survival when receiving limited resection. At the same time, patients with larger tumor size (1.5-2cm) tended to benefit more from lobectomy. This leads to a strong interest in exploring treatment effect heterogeneity over age and tumor size. In CALGB 140503, we removed 2 patients with overall survival times equal to 0, as survival time should logically be greater than 0, and these data points were considered invalid. In the remaining 695 patients, we detected an outlier in tumor size by calculating the $3\sigma$ interval for tumor size, which was found to be $(0.413, 2.551)$. Since the tumor size of 3 exceeded this interval, we concluded it was an outlier. Therefore, we excluded this patient’s data to ensure the robustness of our analysis. After these exclusions, we used data from 694 patients in CALGB 140503.

The National Cancer Database (NCDB) is a clinical oncology database maintained by the American College of Surgeons, and it captured $72\%$ of all newly diagnosed lung cancers. From the NCDB database, we selected a cohort of 17,995 stage 1A NSCLC patients with $\le$ 2 cm tumor and who met all eligibility criteria of CALGB 140503. The NCDB-only analysis based on multivariable Cox proportional hazards model and propensity score-based methods reveals a significant overall benefit of lobectomy over limited resection, which contradicts the findings of CALGB 140503. Unobserved hidden confounders in the NCDB-only analysis could explain the beneﬁt of lobectomy over limited resection. It has been well documented that surgeons and patients tend to choose limited resection over lobectomy if the patient has a bad health status and poor functional respiratory reserve and/or high comorbidity burden (Zhang et al,, 2019; Lee and Altorki, 2023). Unfortunately these confounders were not captured in the NCDB database, and these hidden confounders may have inevitably led to biased estimates of the treatment effects. It is of a great interest to illustrate our proposed method to estimate the HTE. In particular, we want to examine the precision of the proposed HTE estimator for the difference of treatment-specific CRMSTs, conditional on age and tumor size, that can be improved by synthesizing information from CALGB 140503 and NCDB cohorts with the latter subject to possible hidden confounders.

**FIGURE 3:**
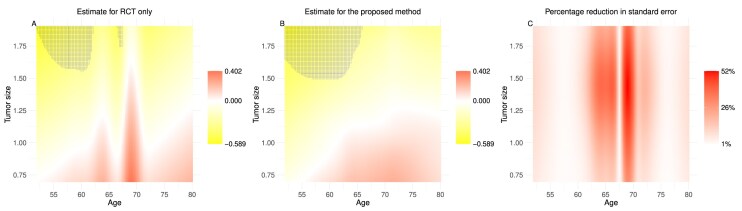
The analysis results of real data.

Our analysis considers the time to death as the survival time and takes age and tumor size as the covariates of interest in a two-dimensional context. The restricted time horizon is set at 3 years. Table 2 displays the descriptive statistics of age and tumor size for the CALGB 140503 and NCDB cohorts. Figure 3 summarizes the results from the trial data-based only and proposed integrative methods. Specifically, the first two panels show the point estimates of the HTE and highlight in blue the regions where the treatment effects are statistically significant, as indicated by $95\%$ confidence intervals that do not include zero.

**TABLE 2:** Distribution of age and tumor size for CALGB 140503 and NCDB cohorts.

	NCDB		CALGB 140503	
	Lobectomy	Limited Resection	Lobectomy	Limited Resection
	(N=14505)	(N=3490)	(N=355)	(N=339)
**age (years)**				
Mean (SD)	65.3 (9.64)	67.6 (9.81)	67.1 (8.67)	67.2 (8.73)
Median [Min, Max]	66.0 [38.0, 89.0]	68.0 [38.0,89.0]	67.5 [43.2,88.9]	68.3 [37.8,89.7]
**tumor size (cm)**				
Mean (SD)	1.52 (0.369)	1.40 (0.389)	1.48 (0.355)	1.48 (0.350)
Median [Min, Max]	1.50 [0.400, 2.00]	1.50 [0.400, 2.00]	1.50 [0.600, 2.50]	1.50 [0.400, 2.30]

In general, there is a trend where patients with large tumor size benefit more from lobectomy compared to limited resection, while patients with small tumor size and older age benefit more from limited resection over lobectomy. The blue regions are more extensive in the second panel, which represents the proposed integrative approach. This indicates that the proposed method identifies a broader range of patient groups where the treatment effects are statistically significant. Specifically, the blue regions highlight that patients with larger tumor sizes ($>$ 1.5 cm) and younger ages ($<$ 65 years) show statistically significant benefits from lobectomy. The third panel illustrates the percentage reduction in standard error achieved by the proposed integrative approach compared to the trial data-based only approach. This reduction demonstrates that the proposed integrative method provides HTE estimates with lower standard errors and narrower confidence intervals. These findings indicate that the proposed integrative method enhances estimation efficiency in real-world applications.

## 6 DISCUSSION

We proposed an integrative estimator of the HTE by combining evidence from the RCT and the RWD in the presence of right censoring. We avoided the assumption of no biases for the RWD and instead defined a bias function to account for various biases in the RWD. The proposed method considered the HTE in a fully nonparametric form, making it flexible, model-free, and data-driven, and hence more practical for use in various applications. Our research aimed to increase the efficiency of the HTE estimation by leveraging supplemental information provided by biased RWD, as verified by our numerical studies.

In the introduction, we emphasized the crucial role that the HTE plays in precision medicine. Individualized treatment and precision medicine are frequently used interchangeably. Since the HTE provides guidance regarding which treatment strategy should be adopted, our proposed framework is closely related to the individualized treatment regime, which involves a decision rule that assigns treatments based on patients’ characteristics. Recent studies, such as Chu et al. (2022) and Zhao et al. (2025), have explored using data from various sources for the individualized treatment regime. Our research sheds light on the potential application of treatment effects for survival outcomes in precision medicine or individualized treatment, making it a valuable contribution to the field.

Our work also has several limitations that warrant discussion. Firstly, the proposed nonparametric estimators may suffer from boundary effects, which is a common issue in nonparametric statistics. Specifically, point estimates of the HTE in boundary regions could be less accurate than those at interior points due to slower convergence rates of nonparametric estimators around the boundary. Secondly, when using the sieve method to approximate the HTE and the bias function, the dimensionality of the covariate $\boldsymbol{X}$ should not be high. Also, we have thus far focused on non-time-varying treatments. Moving forward, marginal structural models (Yang et al., 2018; Yang et al., 2020) offer considerable potential to enhance interpretability in these contexts and constitute a promising direction for future research on data integration.

## Supplementary Material

ujaf131_Supplemental_Files Web Appendices referenced in Sections 1–5 and the R code used for the simulation study are available with this paper at the Biometrics website on Oxford Academic.

## Data Availability

The manuscript uses the data from CALGB 140503 and NCDB to illustrate the proposed method. CALGB 140503 can be requested from https://nctn-data-archive.nci.nih.gov/, and NCDB Participant User Files (PUFs) can be requested from https://www.facs.org/quality-programs/cancer-programs/national-cancer-database/.
