# Supplementary material for: Statistical inference for heterogeneous treatment effect with right-censored data from synthesizing randomized clinical trials and real-world data
Source: Biometrics. 2025 Oct 8;81(4):ujaf131. doi: 10.1093/biomtc/ujaf131 (PMC12505326; doi:10.1093/biomtc/ujaf131)
Supplement: ujaf131_Supplemental_Files — Web Appendices referenced in Sections 1–5 and the R code used for the simulation study are available with this paper at the Biometrics website on Oxford Academic. [file ujaf131_supplemental_files.zip › supp.pdf]

BIOMETRICS 64, 1–58 DOI: 000
December 2008

Supplementary Materials for "Statistical Inference for Heterogeneous Treatment Effect with Right-censored Data from Synthesizing Randomized Clinical Trials and Real-world Data" by

Guangcai Mao

Department of Biostatistics and Bioinformatics, Duke University, Durham, North Carolina, 27710, U.S.A.

email: maoguangcai@ccnu.edu.cn

and

Shu Yang

Department of Statistics, North Carolina State University, Raleigh, North Carolina 27695, U.S.A.

email: syang24@ncsu.edu

and

Xiaofei Wang

Department of Biostatistics and Bioinformatics, Duke University, Durham, North Carolina 27710, U.S.A.

email: xiaofei.wang@duke.edu

This paper has been submitted for consideration for publication in *Biometrics*

The supplementary material is organized as the following. Web Appendix A provides the technical details and definitions of the notations mentioned in the main paper. Web Appendix B presents some additional assumptions for studying the theoretical results of the proposed method. Web Appendix C provides some propositions mentioned in the main paper. Web Appendix D contains some lemmas for proving the theoretical properties. Web Appendix E provides proof of the theoretical properties of the paper. In Web Appendix F, we present the simulation results of Case 2 and Case 3 in Section 5.1 of the main paper, as well as an additional simulation study.

Let $Z_1, \ldots, Z_{n'}$ be the i.i.d. random variables with probability distribution $\mathbb{Q}$, and $\mathbb{Q}_{n'}$ be the empirical measure of these random variables. For a function f, we agree on $\mathbb{Q}_{n'} f = (n')^{-1} \sum_{i=1}^{n'} f(Z_i)$ and $\mathbb{Q} f = \int f(z) \mathrm{d}\mathbb{Q}(z)$. Let $\|f\|_2 = \{\int f^2(z) \mathrm{d}z\}^{1/2}$ and $\|f\|_\infty = \sup_z |f(z)|$ denote the L_2 norm and supremum norm of f, respectively. Furthermore, for a random function $\widehat{f}(z) = \widehat{f}(z; Z_1, \ldots, Z_{n'})$, which is a measurable function concerning z given observations $Z_1, \ldots, Z_{n'}$, we agree on $E_r\{\widehat{f}(Z)\} = \int \widehat{f}(z) \mathrm{d}\mathbb{Q}(z) = E\{f(Z)\}|_{f=\widehat{f}}$ and $\mathrm{Var}_r\{\widehat{f}(Z)\} = \int \widehat{f}^2(z) \mathrm{d}\mathbb{Q}(z) - \{\int \widehat{f}(z) \mathrm{d}\mathbb{Q}(z)\}^2 = \mathrm{Var}\{f(Z)\}|_{f=\widehat{f}}$. Let $\mathbb{P}_1$ and $\mathbb{P}_0$ denote the probability distribution of $(Y, \Delta, \boldsymbol{X}, A, S = 1)$ and $(Y, \Delta, \boldsymbol{X}, A, S = 0)$, respectively. In the following, we use c_j to denote a generic positive constant with an appropriate subscript j for $j = 0, 1, \ldots,$ and c_j may represent different values in different contexts or lines.

Web Appendix A: Technical details and notations

A.1 Sobolev space and penalties

For a multi-index vector $k = (k_1, \ldots, k_p)$ of non-negative integers and a differentiable function $f(\boldsymbol{x})$ with $\boldsymbol{x} = (x_1, \ldots, x_p)^{\mathrm{T}}$, we define $|k| = k_1 + \ldots + k_p$ and the corresponding k-th order derivative of $f(\boldsymbol{x})$ as $f^{(k)}(\boldsymbol{x}) = \partial^k f(\boldsymbol{x}) / (\partial x_1^{k_1} \ldots \partial x_p^{k_p})$. For $m_1' > p/2$, we define the m_1'-th

order Sobolev space $\mathcal{H}_1$ as

$$\mathcal{H}_1 = \Big\{ f:\Omega\to\mathbb{R} \,\Big|\, f^{(j)} \text{ is absolutely continuous for } |j|=0,1,\ldots,(m_1'-1), \text{ and } f^{(k)}\in L_2(\Omega) \text{ for } |k|=m_1' \Big\},$$

where $L_2(\Omega)$ is the collection of all square-integrable functions defined on Ω. Similarly, we define the m_0'-th order Sobolev space $\mathcal{H}_0$ for $m_0' > p/2$. For $p/2 < m_1 \leqslant m_1'$, $p/2 < m_0 \leqslant m_0'$, $k=(k_1,\ldots,k_p)$, $k'=(k_1',\ldots,k_p')$, $f,\widetilde{f}\in\mathcal{H}_1$, and $g,\widetilde{g}\in\mathcal{H}_0$, define

$$J_1(f,\widetilde{f}) = \sum_{|k|=m_1}\frac{m_1!}{\prod_{j=1}^p k_j!}\int_\Omega f^{(k)}(\boldsymbol{x})\widetilde{f}^{(k)}(\boldsymbol{x})\mathrm{d}\boldsymbol{x},$$
$$J_0(g,\widetilde{g}) = \sum_{|k'|=m_0}\frac{m_0!}{\prod_{j=1}^p k_j'!}\int_\Omega g^{(k')}(\boldsymbol{x})\widetilde{g}^{(k')}(\boldsymbol{x})\mathrm{d}\boldsymbol{x}.$$

A.2 Reproducing kernel Hilbert space

Suppose that $n_1/n\to\varrho$ as $n\to\infty$, where $0<\varrho<1$. Let $\sigma_{0s}^2(\boldsymbol{x})$ denote the probability limit of $\widehat{\sigma}_s^2(\boldsymbol{x})$, and $q_1(\boldsymbol{x})$ and $q_0(\boldsymbol{x})$ denote the density functions of $\boldsymbol{X}$ for the RCT and RWD, respectively. For $f,\widetilde{f}\in\mathcal{H}_1$ and $g,\widetilde{g}\in\mathcal{H}_0$, define

$$V_1(f,\widetilde{f}) = \int_\Omega\left[\varrho\left\{\sigma_{01}^2(\boldsymbol{x})\right\}^{-1}q_1(\boldsymbol{x}) + (1-\varrho)\left\{\sigma_{00}^2(\boldsymbol{x})\right\}^{-1}q_0(\boldsymbol{x})\right]f(\boldsymbol{x})\widetilde{f}(\boldsymbol{x})\mathrm{d}\boldsymbol{x},$$
$$V_0(g,\widetilde{g}) = (1-\varrho)\int_\Omega\left\{\sigma_{00}^2(\boldsymbol{x})\right\}^{-1}g(\boldsymbol{x})\widetilde{g}(\boldsymbol{x})q_0(\boldsymbol{x})\mathrm{d}\boldsymbol{x}.$$

Then $\mathcal{H}_1$ is a reproducing kernel Hilbert space (RKHS) endowed with the inner product $\langle f,\widetilde{f}\rangle_{\mathcal{H}_1} = V_1(f,\widetilde{f})+\gamma_1 J_1(f,\widetilde{f})$ and the norm $\|f\|_{\mathcal{H}_1}^2=\langle f,f\rangle_{\mathcal{H}_1}$, and $\mathcal{H}_0$ is a RKHS endowed with the inner product $\langle g,\widetilde{g}\rangle_{\mathcal{H}_0} = V_0(g,\widetilde{g})+\gamma_0 J_0(g,\widetilde{g})$ and the norm $\|g\|_{\mathcal{H}_0}^2=\langle g,g\rangle_{\mathcal{H}_0}$. Define the product space $\mathcal{H}=\mathcal{H}_1\times\mathcal{H}_0$ endowed with the inner product $\langle \boldsymbol{h},\widetilde{\boldsymbol{h}}\rangle_{\mathcal{H}} = \langle f,\widetilde{f}\rangle_{\mathcal{H}_1}+\langle g,\widetilde{g}\rangle_{\mathcal{H}_0}$ and the norm $\|\boldsymbol{h}\|_{\mathcal{H}}^2=\langle\boldsymbol{h},\boldsymbol{h}\rangle_{\mathcal{H}}=\|f\|_{\mathcal{H}_1}^2+\|g\|_{\mathcal{H}_0}^2$ for $\boldsymbol{h}=(f,g)\in\mathcal{H}$ and $\widetilde{\boldsymbol{h}}=(\widetilde{f},\widetilde{g})\in\mathcal{H}$.

Let $K_1(\cdot,\cdot)$ and $K_0(\cdot,\cdot)$ denote the reproducing kernels for $\mathcal{H}_1$ and $\mathcal{H}_0$, respectively, then $K_1(\cdot,\cdot)$ and $K_0(\cdot,\cdot)$ are known to have the properties $K_{1\boldsymbol{x}}(\cdot)\equiv K_1(\boldsymbol{x},\cdot)=K_1(\cdot,\boldsymbol{x})\in\mathcal{H}_1$ and $\langle K_{1\boldsymbol{x}},f\rangle_{\mathcal{H}_1}=f(\boldsymbol{x})$, and $K_{0\boldsymbol{x}}(\cdot)\equiv K_0(\boldsymbol{x},\cdot)=K_0(\cdot,\boldsymbol{x})\in\mathcal{H}_0$ and $\langle K_{0\boldsymbol{x}},g\rangle_{\mathcal{H}_0}=g(\boldsymbol{x})$. We further define two nonnegative definite and self-adjoint linear operators W_{γ_1} and W_{γ_0}

respectively defined on $\mathcal{H}_1$ and $\mathcal{H}_0$ such that $\langle W_{\gamma_1} f, \widetilde{f}\rangle_{\mathcal{H}_1} = \gamma_1 J_1(f, \widetilde{f})$ for $f, \widetilde{f} \in \mathcal{H}_1$, and $\langle W_{\gamma_0} g, \widetilde{g}\rangle_{\mathcal{H}_0} = \gamma_0 J_0(g, \widetilde{g})$ for $g, \widetilde{g} \in \mathcal{H}_0$. Then $\langle f, \widetilde{f}\rangle_{\mathcal{H}_1} = V_1(f, \widetilde{f}) + \langle W_{\gamma_1} f, \widetilde{f}\rangle_{\mathcal{H}_1}$ and $\langle g, \widetilde{g}\rangle_{\mathcal{H}_0} = V_0(g, \widetilde{g}) + \langle W_{\gamma_0} g, \widetilde{g}\rangle_{\mathcal{H}_0}$.

A.3 Notations

Let $\dot{\widehat{\boldsymbol{U}}}_n(\tau, \lambda)$ denote the second order Fréchet derivative operator of $\widehat{\ell}_n(\tau, \lambda)$ with respective to (τ, λ), $\dot{\boldsymbol{U}}(\tau, \lambda)$ denote the probability limit of $\dot{\widehat{\boldsymbol{U}}}_n(\tau, \lambda)$, and $\dot{\boldsymbol{W}}_{\boldsymbol{\gamma}}$ denote the linear operator map $\mathcal{H}$ to $\mathcal{H}$ such that $\dot{\boldsymbol{W}}_{\boldsymbol{\gamma}}[\boldsymbol{h}] = (W_{\gamma_1} f, W_{\gamma_0} g)$ for $\boldsymbol{h} = (f, g) \in \mathcal{H}$. Let $\dot{\boldsymbol{U}}_{\boldsymbol{\gamma}}(\tau, \lambda) = \dot{\boldsymbol{U}}(\tau, \lambda) + \dot{\boldsymbol{W}}_{\boldsymbol{\gamma}}$ and $\dot{\boldsymbol{U}}_{\boldsymbol{\gamma}}^{-1}(\tau, \lambda)$ denote the inverse operator of $\dot{\boldsymbol{U}}_{\boldsymbol{\gamma}}(\tau, \lambda)$. Furthermore, let $\tau_0^* = \tau_0 - b_\tau$, $\lambda_0^* = \lambda_0 - b_\lambda$ with $(b_\tau, b_\lambda) = \dot{\boldsymbol{U}}_{\boldsymbol{\gamma}}^{-1}(\tau_0, \lambda_0)[(W_{\gamma_1}\tau_0, W_{\gamma_0}\lambda_0)]$, and $\boldsymbol{\Sigma}$ be the covariance matrix such that, for every $\boldsymbol{\omega} = (\omega_1, \omega_0)^{\mathrm{T}} \in \mathbb{R}^2$,

$$\boldsymbol{\omega}^{\mathrm{T}}\boldsymbol{\Sigma}\boldsymbol{\omega} = \lim_{n\to\infty}\left\{V_1(K_1^*, K_1^*) + V_0(K_0^*, K_0^*) + 2(1-\varrho)\int_\Omega \left\{\sigma_{00}^2(\boldsymbol{x})\right\}^{-1} K_1^*(\boldsymbol{x})K_0^*(\boldsymbol{x})q_0(\boldsymbol{x})\mathrm{d}\boldsymbol{x}\right\}$$

with $(K_1^*, K_0^*) = \dot{\boldsymbol{U}}_{\boldsymbol{\gamma}}^{-1}(\tau_0, \lambda_0)[(\omega_1\gamma_1^{p/(4m_1)}K_{1\boldsymbol{x}_0}, \omega_0\gamma_0^{p/(4m_0)}K_{0\boldsymbol{x}_0})]$.

Web Appendix B: Conditions

Condition S1: $\phi_1(\cdot), \ldots, \phi_{r_1}(\cdot)$ belong to $\mathcal{H}_1$, and $\psi_1(\cdot), \ldots, \psi_{r_0}(\cdot)$ belong to $\mathcal{H}_0$.

Condition S2: For $f \in \mathcal{H}_1$, $g \in \mathcal{H}_0$, there exist some $f_n(\cdot) \in \Phi_n$, $g_n(\cdot) \in \Psi_n$, $\kappa_1 > \kappa_1' \geqslant 0$, and $\kappa_0 > \kappa_0' \geqslant 0$ such that

$$\begin{aligned} \|f_n - f\|_\infty &= O(n^{-\kappa_1}), \quad \sup_{|k|=m_1}\left\|f_n^{(k)} - f^{(k)}\right\|_\infty = O(n^{-\kappa_1'}), \\ \|g_n - g\|_\infty &= O(n^{-\kappa_0}), \quad \sup_{|k'|=m_0}\left\|g_n^{(k')} - g^{(k')}\right\|_\infty = O(n^{-\kappa_0'}). \end{aligned}$$

Condition S3: $\widehat{D}$ is uniformly bounded and belongs to a class of functions $\mathscr{D}$, and $\{\widehat{\sigma}_s^2(\boldsymbol{x})\}^{-1}$ is uniformly bounded away from zero and belongs to a class of functions $\mathscr{A}$, such that $\max\{\log N(\epsilon, \mathscr{D}, \|\cdot\|_\infty), \log N(\epsilon, \mathscr{A}, \|\cdot\|_\infty)\} < c_0\epsilon^{-\upsilon}$ for some constants $c_0 > 0$ and $0 < \upsilon < 2$, where $N(\epsilon, \mathscr{D}, \|\cdot\|_\infty)$ and $N(\epsilon, \mathscr{A}, \|\cdot\|_\infty)$ are covering numbers of $\mathscr{D}$ and $\mathscr{A}$ under supremum norm, and $s = 0, 1$.

CONDITION S4: There exist some constants $0 < c_1 < 1$ and $c_2 > 0$ such that $c_1 \leqslant \widehat{e}(X) \leqslant 1 - c_1$, $\widehat{G}_T(L \mid \boldsymbol{X}, A, S = 1) \geqslant c_2$, and $\widehat{G}_C(L \mid \boldsymbol{X}, A, S = 1) \geqslant c_2$.

CONDITION S5: $c_3^{-1} \leqslant q_s(\boldsymbol{x}) \leqslant c_3$, where $s = 0, 1$.

CONDITION S6: $\sum_{a=0}^{1} \|\Theta_a\|_2 + \|\widehat{e} - e\|_2 \times \sum_{a=0}^{1} \|\widehat{\mu}_a - \mu_a\|_2 = o_P(1)$, where

$$\begin{aligned}\Theta_A(\boldsymbol{X}) &= \int_0^L \Bigg[G_T(t \mid \boldsymbol{X}, A, S = 1) \int_0^t \frac{\widehat{G}_T(u \mid \boldsymbol{X}, A, S = 1) - G_T(u \mid \boldsymbol{X}, A, S = 1)}{\widehat{G}_T(u \mid \boldsymbol{X}, A, S = 1)} \\ &\quad \times \mathrm{d}\left\{ \frac{G_C(u \mid \boldsymbol{X}, A, S = 1) - \widehat{G}_C(u \mid \boldsymbol{X}, A, S = 1)}{\widehat{G}_C(u \mid \boldsymbol{X}, A, S = 1)} \right\} \Bigg] \mathrm{d}t.\end{aligned}$$

CONDITION S7: $\sum_{a=0}^{1} \|\Theta_a\|_2 + \|\widehat{e} - e\|_2 \times \sum_{a=0}^{1} \|\widehat{\mu}_a - \mu_a\|_2 = O_P(\delta_n)$, where $\delta_n = n^{-1/2}\gamma_1^{-p/(4m_1)} + n^{-1/2}\gamma_0^{-p/(4m_0)} + \gamma_1^{1/2} + \gamma_0^{1/2}$.

CONDITION S8: $n^{-1/2}\{\gamma_s^{(-2m_s v - 4p + pv)/(8m_s)} + \gamma_s^{-(6m_s - p)p/(8m_s^2)}\} = o(1)$, where $s = 0, 1$.

CONDITION S9: $(n^{-\kappa_1} + n^{-\kappa_0}) = O(\delta_n)$.

CONDITION S10: $\sum_{a=0}^{1} \|\Theta_a\|_2 + \|\widehat{e} - e\|_2 \times \sum_{a=0}^{1} \|\widehat{\mu}_a - \mu_a\|_2 = o_P(n^{-1/2})$.

CONDITION S11: $\delta_n(\gamma_1^{-p/(4m_1)} + \gamma_0^{-p/(4m_0)}) = o(1)$.

CONDITION S12: $n^{1/2}(n^{-\kappa_1}\gamma_1^{-1/2} + n^{-\kappa_0}\gamma_0^{-1/2})\delta_n = o(1)$ and $n^{1/2}(n^{-\kappa_1'} + n^{-\kappa_0'})\delta_n = O(1)$.

CONDITION S13: $\|\widehat{\sigma}_s^2 - \sigma_{0s}^2\|_\infty = o_P(1)$, where $s = 0, 1$.

CONDITION S14: $\|\widehat{D} - D_0\|_\infty = o_P(1)$, where D_0 is the probability limit of $\widehat{D}$.

CONDITION S15: $\widehat{\sigma}^2(\boldsymbol{X}, S) - \mathrm{Var}_r(\widehat{D} \mid \boldsymbol{X}, S) = o_P(1)$.

CONDITION S16: There exist some positive constants κ^* and $\kappa^\dagger$ such that $\rho(\boldsymbol{A}^{\mathrm{T}}\boldsymbol{A}/n) \asymp n^{-\kappa^*}$ and $\rho_{\max}(\boldsymbol{P}) = O(n^{\kappa^\dagger})$ almost surely, where $\boldsymbol{A}$ and $\boldsymbol{P}$ are defined in the first paragraph of Subsection E.5, $\rho(\boldsymbol{A}^{\mathrm{T}}\boldsymbol{A}/n)$ is the eigenvalues of $\boldsymbol{A}^{\mathrm{T}}\boldsymbol{A}/n$ and $\rho_{\max}(\boldsymbol{P})$ is the maximum eigenvalue of $\boldsymbol{P}$.

Condition S17: $(n^{-\kappa_1} + n^{-\kappa_0}) = o(n^{-1/2})$ and $n^{\kappa^* + \kappa^\dagger}(\gamma_1 + \gamma_0) = o(n^{-1/2})$.

Condition S1 ensures that the estimators $\widehat{\tau}_n$ and $\widehat{\lambda}_n$ are within the subspaces of $\mathcal{H}_1$ and $\mathcal{H}_0$, respectively. Since the theoretical properties are established within the framework of reproducing kernel Hilbert space, this condition is indispensable. However, this condition is mild and easily satisfied. For example, If B-splines or power series with appropriate orders, or reproducing kernels with appropriate smoothness, are selected as the sieve basis, Condition S1 would be met. Condition S2 requires uniform approximation rates to the function and its derivatives. If one chooses the reproducing kernel approximation with some appropriate kernel such as Gaussian kernel, and chooses all the observation data as the knots, then Condition S2 will satisfied with $\kappa_1 = m_1'/p$, $\kappa_1' = (m_1' - m_1)/p$, $\kappa_0 = m_0'/p$ and $\kappa_0' = (m_0' - m_0)/p$. Additionally, If f and g have continuous m_1'-th and m_0'-th order derivatives, respectively, and further choose B-splines or power series as the sieve basis, and in each dimension, the maximum length between the spline knots is dominated by the inverse of the number of spline knots, then $\|f_n - f\|_\infty = O(r_1^{-m_1'/p})$, $\sup_{|k|=m_1} \|f_n^{(k)} - f^{(k)}\|_\infty = O(r_1^{-(m_1'-m_1)/p})$, $\|g_n - g\|_\infty = O(r_0^{-m_0'/p})$, and $\sup_{|k'|=m_0} \|g_n^{(k')} - g^{(k')}\|_\infty = O(r_0^{-(m_0'-m_0)/p})$. Moreover, if $r_1 \asymp n^{\nu_1}$ and $r_0 \asymp n^{\nu_0}$, then this condition is satisfied with $\kappa_1 = m_1'\nu_1/p$, $\kappa_0 = m_0'\nu_0/p$, $\kappa_1' = (m_1' - m_1)\nu_1/p$, and $\kappa_0' = (m_0' - m_0)\nu_0/p$. Such a condition appears in Newey (1997). Conditions S3–S5 are technical. Condition S3 is a regularization condition and controls the complexities of $\widehat{D}$ and $\widehat{\sigma}_s^2(\boldsymbol{x})$. Especially, Condition S3 implies that $\widehat{D}$ and $\widehat{\sigma}_s^2(\boldsymbol{x})$ belong to Donsker classes. Condition S4 ensures $\widehat{D}$ to be well defined. It can be easily satisfied for commonly used estimation methods such as some survival models for $G_T(t \mid \boldsymbol{X}, A, S = 1)$ and $G_C(t \mid \boldsymbol{X}, A, S = 1)$. Condition S5 constitutes a vital prerequisite for Lemma S4. Similar or equivalent conditions are also imposed in Cox (1984), Cox (1988), O'Sullivan (1993), and Gu (2013). Under Condition S5, the square of L_2 norm is equivalent to V_1 and V_0, that is, $c_4^{-1}\|f\|_2^2 \leqslant V_1(f, f) \leqslant c_4\|f\|_2^2$ and $c_5^{-1}\|g\|_2^2 \leqslant V_0(g, g) \leqslant c_5\|g\|_2^2$ for $f \in \mathcal{H}_1$ and $g \in \mathcal{H}_0$.

The remaining conditions are the specific technical conditions required for the theoretical properties in the main paper. Condition S6 is used to derive the robustness of the proposed estimators, as shown in Theorem 1. Conditions S7–S9 are used for studying the convergence rate of the proposed estimator. The conditions in Remark 1 which presented in the main paper imply that $n^{-1/2}\{\gamma_s^{(-2m_s v-4p+pv)/(8m_s)} + \gamma_s^{-(6m_s-p)p/(8m_s^2)}\} \asymp \{n^{-(2m-p)(2-v)/(8m+4p)} + n^{-(2m-p)^2/(8m^2+4mp)}\} = o(1)$ by $m > p/2$ and $0 < v < 2$. Furthermore, if $r_1 \asymp r_0 \asymp n^{\nu}$, then Condition S9 can be satisfied by taking $\nu = p/(2m+p) < 1/2$. Thus, Conditions S8–S9 remain valid under the conditions described in Remark 1 which presented in the main paper. Conditions S10–S14 are imposed for deriving the asymptotic normality. Condition S10 is imposed to ignore the error caused by the nuisance functions. By Theorem 1, various combinations of the convergence rates of $\widehat{G}_T$ and $\widehat{G}_C$ fulfill this condition. For instance, if parametric models are selected to fit G_T and G_C, and they are correctly specified, then $\sum_{a=0}^{1} \|\Theta_a\|_2 = o_P(n^{-1})$. Alternatively, if fully nonparametric estimation is employed for G_T and G_C, Condition S10 would be satisfied under certain smoothness conditions for G_T and G_C. For the conditions outlined in Remark 1 which is presented in the main paper, Condition S11 is automatically satisfied since $m > p/2$. Furthermore, if $m_1' = m_0' = m'$, $r_1 \asymp r_0 \asymp n^{\nu}$, then Condition S12 is fulfilled when $\max\{p/(2m'), p^2/\{(m'-m)(4m+2p)\}\} < \nu < 1$. Conditions S13–S14 enhance pointwise convergence to uniform convergence, but they are rather mild conditions and often easily satisfied by common estimation methods. Uniform convergence is a technical enhancement. It does not significantly limit the applicability of the estimators, rather, it is a reasonable and common condition in both theoretical and practical contexts, especially in the field of survival analysis. Interestingly, here, $\widehat{\sigma}_s^2(\boldsymbol{X})$ and $\widehat{D}$, as estimators of $\mathrm{Var}(D \mid \boldsymbol{X}, S = s)$ and D, do not need to be consistent. Conditions S15–S17 are used for studying the efficiency of the proposed estimator. Choosing B-splines as the sieve basis, with the maximum length between spline knots in each dimension dominated by

the inverse of the number of knots, ensures that Condition S16 holds with $n^{-\kappa^*} \asymp (r_1+r_0)^{-1}$ and $n^{\kappa^\dagger} \asymp (r_1^3+r_0^3)$. Furthermore, if $r_1 \asymp r_0 \asymp n^{\nu}$, $\gamma_1 \asymp \gamma_0 \asymp n^{-\varsigma}$, and $m_1' = m_0' = m'$, then Condition S17 holds under the condition $p/(2m') < \nu < (2\varsigma - 1)/8$. This condition implies that the true functions τ_0 and λ_0 should exhibit high smoothness, the number of spline knots should be moderate, and the penalized parameters should be sufficiently small.

Web Appendix C: Propositions

Proposition S1: Suppose that Assumptions 1–2 in the main paper hold, then

$$E(R \mid \boldsymbol{X}, S=1) = \mu_1(\boldsymbol{X}) - \mu_0(\boldsymbol{X}) = \tau(\boldsymbol{X}).$$

Proof. It follows that

$$\begin{aligned} E(R_1 \mid A=1, \boldsymbol{X}, S=1) &= E\{T(1) \wedge L \mid A=1, \boldsymbol{X}, S=1\}\frac{1}{e(\boldsymbol{X})} - \frac{1-e(\boldsymbol{X})}{e(\boldsymbol{X})}\mu_1(\boldsymbol{X}) \\ &= \mu_1(\boldsymbol{X}), \\ E(R_1 \mid A=0, \boldsymbol{X}, S=1) &= E\{\mu_1(\boldsymbol{X}) \mid A=0, \boldsymbol{X}, S=1\} = \mu_1(\boldsymbol{X}), \end{aligned}$$

which implies that $E(R_1 \mid \boldsymbol{X}, S=1) = \mu_1(\boldsymbol{X})$. Similarly, we have $E(R_0 \mid \boldsymbol{X}, S=1) = \mu_0(\boldsymbol{X})$. Combining Assumption 1 in the main paper, we conclude that

$$\begin{aligned} E(R \mid \boldsymbol{X}, S=1) &= \mu_1(\boldsymbol{X}) - \mu_0(\boldsymbol{X}) \\ &= E\{T(1) \wedge L \mid A=1, \boldsymbol{X}, S=1\} - E\{T(0) \wedge L \mid A=0, \boldsymbol{X}, S=1\} \\ &= E\{T(1) \wedge L \mid \boldsymbol{X}, S=1\} - E\{T(0) \wedge L \mid \boldsymbol{X}, S=1\} \\ &= E\{T(1) \wedge L - T(0) \wedge L \mid \boldsymbol{X}, S=1\} \\ &= \tau(\boldsymbol{X}). \end{aligned}$$

Hence, we prove Proposition S1.

Proposition S2: Suppose that Assumption 2 in the main paper holds, then

$$E\left(\widetilde{T}_L \mid \boldsymbol{X}, A, S=1\right) = E\left(T_L \mid \boldsymbol{X}, A, S=1\right).$$

Proof. By Assumption 2 in the main paper,

$$
\begin{aligned}
& E\left\{ Y_L \widetilde{\Delta} G_C^{-1}(Y_L \mid \boldsymbol{X}, A, S=1) \,\middle|\, \boldsymbol{X}, A, S=1 \right\} \\
= \;& E\left[E\left\{ Y_L \widetilde{\Delta} G_C^{-1}(Y_L \mid \boldsymbol{X}, A, S=1) \,\middle|\, T_L, \boldsymbol{X}, A, S=1 \right\} \,\middle|\, \boldsymbol{X}, A, S=1 \right] \\
= \;& E\left[E\left\{ T_L \widetilde{\Delta} G_C^{-1}(T_L \mid \boldsymbol{X}, A, S=1) \,\middle|\, T_L, \boldsymbol{X}, A, S=1 \right\} \,\middle|\, \boldsymbol{X}, A, S=1 \right] \\
= \;& E\left\{ T_L G_C^{-1}(T_L \mid \boldsymbol{X}, A, S=1) P\left(\widetilde{\Delta}=1 \mid T_L, \boldsymbol{X}, A, S=1 \right) \,\middle|\, \boldsymbol{X}, A, S=1 \right\} \\
= \;& E\left\{ T_L G_C^{-1}(T_L \mid \boldsymbol{X}, A, S=1) P(C \geqslant T_L \mid T_L, \boldsymbol{X}, A, S=1) \,\middle|\, \boldsymbol{X}, A, S=1 \right\} \\
= \;& E(T_L \mid \boldsymbol{X}, A, S=1). \qquad \text{(S1)}
\end{aligned}
$$

Thus, it is enough to show that

$$
E\left\{ \int_0^L B(t) G_C^{-1}(t \mid \boldsymbol{X}, A, S=1) \mathrm{d}M_C(t) \,\middle|\, \boldsymbol{X}, A, S=1 \right\} = 0. \qquad \text{(S2)}
$$

Following Assumption 2 in the main paper, we have

$$
\begin{aligned}
E\{N_C(t) \mid \boldsymbol{X}, A, S=1\} &= E\big[E\{N_C(t) \mid C, \boldsymbol{X}, A, S=1\} \mid \boldsymbol{X}, A, S=1\big] \\
&= E_C\{I(C \leqslant t) P(C \leqslant T_L \mid C, \boldsymbol{X}, A, S=1) \mid \boldsymbol{X}, A, S=1\} \\
&= -\int_0^t G_T(u \mid \boldsymbol{X}, A, S=1) \mathrm{d}G_C(u \mid \boldsymbol{X}, A, S=1).
\end{aligned}
$$

Thus

$$
\begin{aligned}
& E\left\{ \int_0^L B(t) G_C^{-1}(t \mid \boldsymbol{X}, A, S=1) \mathrm{d}N_C(t) \,\middle|\, \boldsymbol{X}, A, S=1 \right\} \\
= \;& -\int_0^L B(t) G_T(t \mid \boldsymbol{X}, A, S=1) G_C^{-1}(t \mid \boldsymbol{X}, A, S=1) \mathrm{d}G_C(t \mid \boldsymbol{X}, A, S=1).
\end{aligned}
$$

On the other hand,

$$
\begin{aligned}
& E\left\{ \int_0^L B(t) G_C^{-1}(t \mid \boldsymbol{X}, A, S=1) \mathrm{d}Q_C(t) \,\middle|\, \boldsymbol{X}, A, S=1 \right\} \\
= \;& E\left\{ \int_0^L B(t) G_C^{-2}(t \mid \boldsymbol{X}, A, S=1) I(Y_L \geqslant t) \mathrm{d}G_C(t \mid \boldsymbol{X}, A, S=1) \,\middle|\, \boldsymbol{X}, A, S=1 \right\} \\
= \;& \int_0^L B(t) G_C^{-2}(t \mid \boldsymbol{X}, A, S=1) P(Y \geqslant t \mid \boldsymbol{X}, A, S=1) \mathrm{d}G_C(t \mid \boldsymbol{X}, A, S=1) \\
= \;& \int_0^L B(t) G_T(t \mid \boldsymbol{X}, A, S=1) G_C^{-1}(t \mid \boldsymbol{X}, A, S=1) \mathrm{d}G_C(t \mid \boldsymbol{X}, A, S=1),
\end{aligned}
$$

which implies that equation (S2) holds. Thus we prove Proposition S2.

Web Appendix D: Lemmas

Lemma S1: *Suppose that Assumptions 1–2 in the main paper hold, then*

$$E_r\left(\widehat{T}_L - \widetilde{T}_L \mid \boldsymbol{X}, A, S=1\right) = \Theta_A(\boldsymbol{X}),$$

where

$$\widehat{T}_L = Y_L\widetilde{\Delta}\widehat{G}_C^{-1}(Y_L \mid \boldsymbol{X}, A, S=1) - \int_0^L \widehat{B}(t)\widehat{G}_C^{-1}(t \mid \boldsymbol{X}, A, S=1)\mathrm{d}\widehat{M}_C(t),$$

is the corresponding estimator of $\widetilde{T}_L$, $\widehat{B}(t) = t + \int_t^L \widehat{G}_T(u \mid \boldsymbol{X}, A, S=1)\mathrm{d}u/\widehat{G}_T(t \mid \boldsymbol{X}, A, S=1)$ *and* $\widehat{M}_C(t) = N_C(t) + \widehat{Q}_C(t)$ *with* $\widehat{Q}_C(t) = \int_0^t I(Y_L \geqslant u)\widehat{G}_C^{-1}(u \mid \boldsymbol{X}, A, S=1)\mathrm{d}\widehat{G}_C(u \mid \boldsymbol{X}, A, S=1)$, *and*

$$\begin{aligned}\Theta_A(\boldsymbol{X}) &= \int_0^L \Bigg[G_T(t \mid \boldsymbol{X}, A, S=1)\int_0^t \frac{\widehat{G}_T(u \mid \boldsymbol{X}, A, S=1) - G_T(u \mid \boldsymbol{X}, A, S=1)}{\widehat{G}_T(u \mid \boldsymbol{X}, A, S=1)} \\ &\quad \times \mathrm{d}\left\{\frac{G_C(u \mid \boldsymbol{X}, A, S=1) - \widehat{G}_C(u \mid \boldsymbol{X}, A, S=1)}{\widehat{G}_C(u \mid \boldsymbol{X}, A, S=1)}\right\}\Bigg]\mathrm{d}t.\end{aligned}$$

Proof. By the definition of E_r and Proposition S2,

$$\begin{aligned}E_r\left(\widetilde{T}_L \mid \boldsymbol{X}, A, S=1\right) = E\left(\widetilde{T}_L \mid \boldsymbol{X}, A, S=1\right) &= E\left(T_L \mid \boldsymbol{X}, A, S=1\right) \\ &= \int_0^L G_T(t \mid \boldsymbol{X}, A, S=1)\mathrm{d}t. \quad \text{(S3)}\end{aligned}$$

Mimicking the proof of equation (S1), we have

$$\begin{aligned}& E_r\left\{Y_L\widetilde{\Delta}\widehat{G}_C^{-1}(Y_L \mid \boldsymbol{X}, A, S=1)\,\Big|\, \boldsymbol{X}, A, S=1\right\} \\ =\ & E_r\left\{T_L G_C(T_L \mid \boldsymbol{X}, A, S=1)\widehat{G}_C^{-1}(T_L \mid \boldsymbol{X}, A, S=1)\,\Big|\, \boldsymbol{X}, A, S=1\right\} \\ =\ & \int_0^L G_T(t \mid \boldsymbol{X}, A, S=1)G_C(t \mid \boldsymbol{X}, A, S=1)\widehat{G}_C^{-1}(t \mid \boldsymbol{X}, A, S=1)\mathrm{d}t \\ & + \int_0^L tG_T(t \mid \boldsymbol{X}, A, S=1)\mathrm{d}\left\{G_C(t \mid \boldsymbol{X}, A, S=1)\widehat{G}_C^{-1}(t \mid \boldsymbol{X}, A, S=1)\right\}. \quad \text{(S4)}\end{aligned}$$

Furthermore,

$$
\begin{aligned}
& E_r\left\{\int_0^L \widehat{B}(t)\widehat{G}_C^{-1}(t \mid \boldsymbol{X}, A, S=1)\mathrm{d}\widehat{M_C}(t) \,\Big|\, \boldsymbol{X}, A, S=1\right\} \\
= \; & -E_r\left\{\int_0^L \widehat{B}(t)\widehat{G}_C^{-1}(t \mid \boldsymbol{X}, A, S=1)\mathrm{d}Q_C(t) \,\Big|\, \boldsymbol{X}, A, S=1\right\} \\
& +E_r\left\{\int_0^L \widehat{B}(t)\widehat{G}_C^{-1}(t \mid \boldsymbol{X}, A, S=1)\mathrm{d}\widehat{Q}_C(t) \,\Big|\, \boldsymbol{X}, A, S=1\right\} \\
= \; & -E_r\bigg\{\int_0^L \widehat{B}(t)\widehat{G}_C^{-1}(t \mid \boldsymbol{X}, A, S=1)G_C^{-1}(t \mid \boldsymbol{X}, A, S=1) \\
& \times I(Y_L \geqslant t)\mathrm{d}G_C(t \mid \boldsymbol{X}, A, S=1) \,\Big|\, \boldsymbol{X}, A, S=1\bigg\} \\
& +E_r\bigg\{\int_0^L \widehat{B}(t)\widehat{G}_C^{-1}(t \mid \boldsymbol{X}, A, S=1)\widehat{G}_C^{-1}(t \mid \boldsymbol{X}, A, S=1) \\
& \times I(Y_L \geqslant t)\mathrm{d}\widehat{G}_C(t \mid \boldsymbol{X}, A, S=1) \,\Big|\, \boldsymbol{X}, A, S=1\bigg\} \\
= \; & \int_0^L \widehat{B}(t)G_T(t \mid \boldsymbol{X}, A, S=1)\mathrm{d}\left\{G_C(t \mid \boldsymbol{X}, A, S=1)\widehat{G}_C^{-1}(t \mid \boldsymbol{X}, A, S=1)\right\} \\
= \; & \int_0^L tG_T(t \mid \boldsymbol{X}, A, S=1)\mathrm{d}\left\{G_C(t \mid \boldsymbol{X}, A, S=1)\widehat{G}_C^{-1}(t \mid \boldsymbol{X}, A, S=1)\right\} \\
& +\int_0^L G_T(t \mid \boldsymbol{X}, A, S=1)\bigg[\int_0^t G_T(u \mid \boldsymbol{X}, A, S=1) \\
& \times\widehat{G}_T^{-1}(u \mid \boldsymbol{X}, A, S=1)\mathrm{d}\left\{G_C(u \mid \boldsymbol{X}, A, S=1)\widehat{G}_C^{-1}(u \mid \boldsymbol{X}, A, S=1)\right\}\bigg]\mathrm{d}t, \quad \text{(S5)}
\end{aligned}
$$

by using the Fubini Theorem.

Combining equations (S3)–(S5), we have

$$
\begin{aligned}
& E_r\left(\widehat{T}_L - \widetilde{T}_L \mid \boldsymbol{X}, A, S=1\right) \\
= \; & \int_0^L G_T(t \mid \boldsymbol{X}, A, S=1)\Bigg[\frac{G_C(t \mid \boldsymbol{X}, A, S=1) - \widehat{G}_C(t \mid \boldsymbol{X}, A, S=1)}{\widehat{G}_C(t \mid \boldsymbol{X}, A, S=1)} \\
& -\int_0^t \frac{G_T(u \mid \boldsymbol{X}, A, S=1)}{\widehat{G}_T(u \mid \boldsymbol{X}, A, S=1)}\mathrm{d}\left\{\frac{G_C(u \mid \boldsymbol{X}, A, S=1)}{\widehat{G}_C(u \mid \boldsymbol{X}, A, S=1)}\right\}\Bigg]\mathrm{d}t \\
= \; & \int_0^L G_T(t \mid \boldsymbol{X}, A, S=1)\Bigg[\int_0^t \frac{\widehat{G}_T(u \mid \boldsymbol{X}, A, S=1) - G_T(u \mid \boldsymbol{X}, A, S=1)}{\widehat{G}_T(u \mid \boldsymbol{X}, A, S=1)} \\
& \times\mathrm{d}\left\{\frac{G_C(u \mid \boldsymbol{X}, A, S=1) - \widehat{G}_C(u \mid \boldsymbol{X}, A, S=1)}{\widehat{G}_C(u \mid \boldsymbol{X}, A, S=1)}\right\}\Bigg]\mathrm{d}t \\
= \; & \Theta_A(\boldsymbol{X}).
\end{aligned}
$$

Thus, we complete the proof of Lemma S1.

LEMMA S2: *Suppose that Assumptions 1–2 in the main paper hold, then*

$$E_r\left(\widehat{D} - D \mid \boldsymbol{X}, S = 1\right) = \Gamma_1(\boldsymbol{X}) + \Gamma_2(\boldsymbol{X}),$$

where

$$\begin{aligned}
\Gamma_1(\boldsymbol{X}) &= \sum_{a=0}^{1} \frac{\{\widehat{e}(\boldsymbol{X}) - e(\boldsymbol{X})\}\{\widehat{\mu}_a(\boldsymbol{X}) - \mu_a(\boldsymbol{X})\}}{a\widehat{e}(\boldsymbol{X}) + (1-a)\{1 - \widehat{e}(\boldsymbol{X})\}}, \\
\Gamma_2(\boldsymbol{X}) &= \sum_{a=0}^{1} \frac{ae(\boldsymbol{X}) + (a-1)\{1 - e(\boldsymbol{X})\}}{a\widehat{e}(\boldsymbol{X}) + (1-a)\{1 - \widehat{e}(\boldsymbol{X})\}} \Theta_a(\boldsymbol{X}).
\end{aligned}$$

Proof. For $S = 1$, we have $\widehat{D} - D = \widehat{R} - \widetilde{R}$. Rewrite $\widehat{R}$ as

$$\widehat{R} = \frac{A - \widehat{e}(\boldsymbol{X})}{\widehat{e}(\boldsymbol{X})\{1 - \widehat{e}(\boldsymbol{X})\}} \left\{\widehat{T}_L - \widehat{\mu}_A(\boldsymbol{X})\right\} + \widehat{\mu}_1(\boldsymbol{X}) - \widehat{\mu}_0(\boldsymbol{X}),$$

where $\widehat{\mu}_A(\boldsymbol{X}) = A\,\widehat{\mu}_1(\boldsymbol{X}) + (1-A)\,\widehat{\mu}_0(\boldsymbol{X})$. Using Proposition S2 and Lemma S1, we have

$$\begin{aligned}
&E_r\left(\widehat{R} \mid \boldsymbol{X}, S = 1\right) \\
=\;& E_r\left[\frac{A - \widehat{e}(\boldsymbol{X})}{\widehat{e}(\boldsymbol{X})\{1 - \widehat{e}(\boldsymbol{X})\}} \left\{\widetilde{T}_L - \widehat{\mu}_A(\boldsymbol{X})\right\} + \widehat{\mu}_1(\boldsymbol{X}) - \widehat{\mu}_0(\boldsymbol{X}) \,\middle|\, \boldsymbol{X}, S = 1\right] \\
&+ E_r\left[\frac{A - \widehat{e}(\boldsymbol{X})}{\widehat{e}(\boldsymbol{X})\{1 - \widehat{e}(\boldsymbol{X})\}} \left(\widehat{T}_L - \widetilde{T}_L\right) \,\middle|\, \boldsymbol{X}, S = 1\right] \\
=\;& E_r\left[\frac{A - \widehat{e}(\boldsymbol{X})}{\widehat{e}(\boldsymbol{X})\{1 - \widehat{e}(\boldsymbol{X})\}} \left\{\widetilde{T}_L - \widehat{\mu}_A(\boldsymbol{X})\right\} \,\middle|\, \boldsymbol{X}, S = 1\right] + \widehat{\mu}_1(\boldsymbol{X}) - \widehat{\mu}_0(\boldsymbol{X}) \\
&+ E_r\left(E_r\left[\frac{A - \widehat{e}(\boldsymbol{X})}{\widehat{e}(\boldsymbol{X})\{1 - \widehat{e}(\boldsymbol{X})\}} \left(\widehat{T}_L - \widetilde{T}_L\right) \,\middle|\, \boldsymbol{X}, A, S = 1\right] \,\middle|\, \boldsymbol{X}, S = 1\right), \\
=\;& \frac{e(\boldsymbol{X})}{\widehat{e}(\boldsymbol{X})}\{\mu_1(\boldsymbol{X}) - \widehat{\mu}_1(\boldsymbol{X})\} - \frac{1 - e(\boldsymbol{X})}{1 - \widehat{e}(\boldsymbol{X})}\{\mu_0(\boldsymbol{X}) - \widehat{\mu}_0(\boldsymbol{X})\} \\
&+ \widehat{\mu}_1(\boldsymbol{X}) - \widehat{\mu}_0(\boldsymbol{X}) + \Gamma_2(\boldsymbol{X}),
\end{aligned}$$

combined with Proposition S1,

$$\begin{aligned}
&E_r\left(\widehat{R} - \widetilde{R} \mid \boldsymbol{X}, S = 1\right) \\
=\;& \frac{\widehat{e}(\boldsymbol{X}) - e(\boldsymbol{X})}{\widehat{e}(\boldsymbol{X})}\{\widehat{\mu}_1(\boldsymbol{X}) - \mu_1(\boldsymbol{X})\} + \frac{\widehat{e}(\boldsymbol{X}) - e(\boldsymbol{X})}{1 - \widehat{e}(\boldsymbol{X})}\{\widehat{\mu}_0(\boldsymbol{X}) - \mu_0(\boldsymbol{X})\} + \Gamma_2(\boldsymbol{X}) \\
=\;& \Gamma_1(\boldsymbol{X}) + \Gamma_2(\boldsymbol{X}),
\end{aligned}$$

which proves Lemma S2.

Lemma S3: *Let $\pi_1(\boldsymbol{x})$ and $\pi_0(\boldsymbol{x})$ be some functions which are uniformly bounded away from zero, ϖ be some positive constant such that $0 < \varpi < 1$, and*

$$\ell^\dagger(f,g) = \varpi \int_\Omega \pi_1(\boldsymbol{x}) f^2(\boldsymbol{x}) q_1(\boldsymbol{x}) \mathrm{d}\boldsymbol{x} + (1-\varpi) \int_\Omega \pi_0(\boldsymbol{x}) \{f(\boldsymbol{x}) + g(\boldsymbol{x})\}^2 q_0(\boldsymbol{x}) \mathrm{d}\boldsymbol{x},$$

where $(f,g) \in \mathcal{H}$. Suppose that Condition S5 holds, then there exists some positive constant c_0 such that

$$\ell^\dagger(f,g) \geqslant c_0\{V_1(f,f) + V_0(g,g)\},$$

for all $(f,g) \in \mathcal{H}$.

Proof. It follows from Condition S5 and Hölder inequality that

$$\begin{aligned}
\ell^\dagger(f,g) &= \varpi \int_\Omega \pi_1(\boldsymbol{x}) f^2(\boldsymbol{x}) q_1(\boldsymbol{x}) \mathrm{d}\boldsymbol{x} + (1-\varpi) \int_\Omega \pi_0(\boldsymbol{x}) \{f(\boldsymbol{x}) + g(\boldsymbol{x})\}^2 q_0(\boldsymbol{x}) \mathrm{d}\boldsymbol{x} \\
&\geqslant c_1 \left\{ \int_\Omega f^2(\boldsymbol{x}) \mathrm{d}\boldsymbol{x} + \int_\Omega \{f(\boldsymbol{x}) + g(\boldsymbol{x})\}^2 \mathrm{d}\boldsymbol{x} \right\} \\
&\geqslant c_1 \left[2\int_\Omega f^2(\boldsymbol{x}) \mathrm{d}\boldsymbol{x} + \int_\Omega g^2(\boldsymbol{x}) \mathrm{d}\boldsymbol{x} - 2 \left\{ \int_\Omega f^2(\boldsymbol{x}) \mathrm{d}\boldsymbol{x} \right\}^{1/2} \left\{ \int_\Omega g^2(\boldsymbol{x}) \mathrm{d}\boldsymbol{x} \right\}^{1/2} \right],
\end{aligned}$$

and the equality in the last inequality holds if and only if there exists a non-negative function $\varsigma(\boldsymbol{x})$ which is constant almost everywhere in Lebesgue measure such that $g(\boldsymbol{x}) = -\varsigma(\boldsymbol{x}) f(\boldsymbol{x}) = -\varsigma f(\boldsymbol{x})$.

For $\boldsymbol{h} = (f,g) \in \mathcal{H}$ and $\widetilde{\boldsymbol{h}} = (\widetilde{f}, \widetilde{g}) \in \mathcal{H}$, define the L_2 norm on $\mathcal{H}$ as $\|\boldsymbol{h}\|_2 = (\|f\|_2^2 + \|g\|_2^2)^{1/2}$ and a bilinear functional $B^\dagger : \mathcal{H} \times \mathcal{H} \to \mathbb{R}$ as

$$\begin{aligned}
B^\dagger[\boldsymbol{h}][\widetilde{\boldsymbol{h}}] &= \varpi \int_\Omega \pi_1(\boldsymbol{x}) f(\boldsymbol{x}) \widetilde{f}(\boldsymbol{x}) q_1(\boldsymbol{x}) \mathrm{d}\boldsymbol{x} \\
&\quad + (1-\varpi) \int_\Omega \pi_0(\boldsymbol{x}) \{f(\boldsymbol{x}) + g(\boldsymbol{x})\}\{\widetilde{f}(\boldsymbol{x}) + \widetilde{g}(\boldsymbol{x})\} q_0(\boldsymbol{x}) \mathrm{d}\boldsymbol{x}.
\end{aligned}$$

Obviously, $\ell^\dagger(f,g) = B^\dagger[\boldsymbol{h}][\boldsymbol{h}]$. Then

$$\begin{aligned}
\inf_{\|\boldsymbol{h}\|_2=1} B^\dagger[\boldsymbol{h}][\boldsymbol{h}] &= \inf_{\|f\|_2^2+\|g\|_2^2=1} \ell^\dagger(f,g) \\
&\geqslant \inf_{\|f\|_2^2+\|g\|_2^2=1, g=-\varsigma f} c_1 \left\{ \int_\Omega f^2(\boldsymbol{x})\mathrm{d}\boldsymbol{x} + \int_\Omega \{f(\boldsymbol{x})+g(\boldsymbol{x})\}^2 \mathrm{d}\boldsymbol{x} \right\} \\
&= c_1 \inf_{\varsigma>0} \left(1 + \frac{1-2\varsigma}{1+\varsigma^2}\right) \\
&= \frac{\sqrt{5}-1}{\sqrt{5}+1} c_1,
\end{aligned}$$

which implies that

$$\begin{aligned}
\ell^\dagger(f,g) = B^\dagger[\boldsymbol{h}][\boldsymbol{h}] &\geqslant \left(\inf_{\|\boldsymbol{h}\|_2=1} B^\dagger[\boldsymbol{h}][\boldsymbol{h}]\right) \cdot \|\boldsymbol{h}\|_2^2 \\
&\geqslant \frac{\sqrt{5}-1}{\sqrt{5}+1} c_1 \|\boldsymbol{h}\|_2^2 \\
&= \frac{\sqrt{5}-1}{\sqrt{5}+1} c_1 \left(\|f\|_2^2 + \|g\|_2^2\right) \\
&\geqslant \frac{\sqrt{5}-1}{\sqrt{5}+1} c_1 \left\{c_2 V_1(f,f) + c_3 V_0(g,g)\right\} \\
&\geqslant c_4 \left\{V_1(f,f) + V_0(g,g)\right\},
\end{aligned}$$

by using Condition S5. Thus, we proves Lemma S3.

Lemma S4: *Suppose that Condition S5 holds, then*

(1) There exist a sequence of eigenfunctions $\varphi_{1\mu} \in \mathcal{H}_1$ *(*$\mu \in \mathbb{N}$*) satisfying* $\sup_{\mu\in\mathbb{N}} \|\varphi_{1\mu}\|_\infty \leqslant c_1$ *and the corresponding non-decreasing sequence of eigenvalues* $\rho_{1\mu}$ *(*$\mu \in \mathbb{N}$*) such that* $V_1(\varphi_{1\mu}, \varphi_{1\mu'}) = \delta_{\mu\mu'}$ *and* $J_1(\varphi_{1\mu}, \varphi_{1\mu'}) = \rho_{1\mu}\delta_{\mu\mu'}$*, where* $\delta_{\mu\mu'}$ *is the Kronecker's delta and* $\mathbb{N} = \{0,1,2,\ldots\}$*. Furthermore, there exist a sequence of eigenfunctions* $\varphi_{0\mu} \in \mathcal{H}_0$ *(*$\mu \in \mathbb{N}$*) satisfying* $\sup_{\mu\in\mathbb{N}} \|\varphi_{0\mu}\|_\infty \leqslant c_0$ *and the corresponding non-decreasing sequence of eigenvalues* $\rho_{0\mu}$ *(*$\mu \in \mathbb{N}$*) such that* $V_0(\varphi_{0\mu}, \varphi_{0\mu'}) = \delta_{\mu\mu'}$ *and* $J_0(\varphi_{0\mu}, \varphi_{0\mu'}) = \rho_{0\mu}\delta_{\mu\mu'}$*.*

(2) $\rho_{1\mu} \asymp \mu^{2m_1/p}$ *and* $\rho_{0\mu} \asymp \mu^{2m_0/p}$ *for sufficiently large* μ*.*

(3) $f = \sum_\mu V_1(f, \varphi_{1\mu})\varphi_{1\mu}$ *and* $g = \sum_\mu V_0(g, \varphi_{0\mu})\varphi_{0\mu}$ *for* $f \in \mathcal{H}_1$ *and* $g \in \mathcal{H}_0$*, where* $\sum_\mu$ *denotes the sum over* $\mathbb{N} = \{0,1,2,\ldots\}$*.*

The conclusion of Lemma S4 appears in many literature, see Cox (1984) on page 799, Cox (1988) on page 699, O'Sullivan (1993) on page 132, Gu (2013) on page 322, and thus we omit the proof.

LEMMA S5: *Suppose that Condition S5 holds, then for $f \in \mathcal{H}_1, g \in \mathcal{H}_0$, and $\boldsymbol{x} \in \Omega$, we have*

$$\begin{aligned} K_{1\boldsymbol{x}}(\cdot) &= \sum_\mu \frac{\varphi_{1\mu}(\boldsymbol{x})}{1+\gamma_1\rho_{1\mu}}\varphi_{1\mu}(\cdot), \quad W_{\gamma_1}\varphi_{1\mu}(\cdot) = \frac{\gamma_1\rho_{1\mu}}{1+\gamma_1\rho_{1\mu}}\varphi_{1\mu}(\cdot), \\ \|f\|^2_{\mathcal{H}_1} &= \sum_\mu |V_1(f,\varphi_{1\mu})|^2(1+\gamma_1\rho_{1\mu}) = \sum_\mu \frac{|\langle f,\varphi_{1\mu}\rangle_{\mathcal{H}_1}|^2}{1+\gamma_1\rho_{1\mu}}, \end{aligned}$$

and

$$\begin{aligned} K_{0\boldsymbol{x}}(\cdot) &= \sum_\mu \frac{\varphi_{0\mu}(\boldsymbol{x})}{1+\gamma_0\rho_{0\mu}}\varphi_{0\mu}(\cdot), \quad W_{\gamma_0}\varphi_{0\mu}(\cdot) = \frac{\gamma_0\rho_{0\mu}}{1+\gamma_0\rho_{0\mu}}\varphi_{0\mu}(\cdot), \\ \|g\|^2_{\mathcal{H}_0} &= \sum_\mu |V_0(g,\varphi_{0\mu})|^2(1+\gamma_0\rho_{0\mu}) = \sum_\mu \frac{|\langle g,\varphi_{0\mu}\rangle_{\mathcal{H}_0}|^2}{1+\gamma_0\rho_{0\mu}}. \end{aligned}$$

LEMMA S6: *Suppose that Condition S5 holds, then for every $\boldsymbol{x} \in \Omega$, we have*

$$\|K_{1\boldsymbol{x}}\|_{\mathcal{H}_1} \leqslant \widetilde{c}_1\gamma_1^{-p/(4m_1)}, \quad \|K_{0\boldsymbol{x}}\|_{\mathcal{H}_0} \leqslant \widetilde{c}_0\gamma_0^{-p/(4m_0)},$$

where $\widetilde{c}_1$ and $\widetilde{c}_0$ do not depend on the choice of $\boldsymbol{x}$. Furthermore,

$$\|f\|_\infty \leqslant \widetilde{c}_1\gamma_1^{-p/(4m_1)}\|f\|_{\mathcal{H}_1}, \quad \|g\|_\infty \leqslant \widetilde{c}_0\gamma_0^{-p/(4m_0)}\|g\|_{\mathcal{H}_0},$$

for every $f \in \mathcal{H}_1$ and $g \in \mathcal{H}_0$.

Lemmas S5 and S6 are similar to Proposition 2.1 and Lemma 3.1 in Shang and Cheng (2013), and Lemma 0.1 and Lemma 0.2 in the supplementary material of Liu, Mao, and Zhao (2020). The proofs essentially proceed along the lines of these literature and are trivial and omitted for the sake of brevity.

LEMMA S7: *Suppose that Condition S5 holds, then for $f \in \mathcal{H}_1$ and $g \in \mathcal{H}_0$ such that $J_1(f,f) \leqslant c_1$ and $J_0(g,g) \leqslant c_0$, we have*

$$\|W_{\gamma_1}f\|_{\mathcal{H}_1} = o(\gamma_1^{1/2}), \quad \|W_{\gamma_0}g\|_{\mathcal{H}_0} = o(\gamma_0^{1/2}).$$

Proof. It follows from Lemmas S4 and S5 that

$$\begin{aligned}\|W_{\gamma_1}f\|_{\mathcal{H}_1}^2 &= \sum_\mu |V_1(W_{\gamma_1}f,\varphi_{1\mu})|^2(1+\gamma_1\rho_{1\mu})\\ &= \sum_\mu \left|V_1\left(\sum_{\mu'} V_1(f,\varphi_{1\mu'})W_{\gamma_1}\varphi_{1\mu'},\varphi_{1\mu}\right)\right|^2(1+\gamma_1\rho_{1\mu})\\ &= \sum_\mu |V_1(f,\varphi_{1\mu})|^2\left(\frac{\gamma_1\rho_{1\mu}}{1+\gamma_1\rho_{1\mu}}\right)^2(1+\gamma_1\rho_{1\mu})\\ &= \gamma_1\sum_\mu |V_1(f,\varphi_{1\mu})|^2\frac{\gamma_1\rho_{1\mu}^2}{1+\gamma_1\rho_{1\mu}}.\end{aligned}$$

Thus, to prove $\|W_{\gamma_1}f\|_{\mathcal{H}_1}=o(\gamma_1^{1/2})$, it suffices to show

$$\sum_\mu |V_1(f,\varphi_{1\mu})|^2\frac{\gamma_1\rho_{1\mu}^2}{1+\gamma_1\rho_{1\mu}}=o(1). \tag{S6}$$

Let $f_{\gamma_1}^\dagger(\mu)=|V_1(f,\varphi_{1\mu})|^2\frac{\gamma_1\rho_{1\mu}^2}{1+\gamma_1\rho_{1\mu}}$, $f^\dagger(\mu)=|V_1(f,\varphi_{1\mu})|^2\rho_{1\mu}$ and $\mathcal{N}(\cdot)$ denotes the discrete measure over $\mathbb{N}$. Then we have

$$|f_{\gamma_1}^\dagger(\mu)|\leqslant f^\dagger(\mu).$$

Furthermore, by Lemmas S4 and S5,

$$J_1(f,f)=J_1\left(\sum_\mu V_1(f,\varphi_{1\mu})\varphi_{1\mu},\sum_\mu V_1(f,\varphi_{1\mu})\varphi_{1\mu}\right)=\sum_\mu |V_1(f,\varphi_{1\mu})|^2\rho_{1\mu}\leqslant c_1,$$

which implies that

$$\int_{\mathbb{N}} f^\dagger(\mu)\mathrm{d}\mathcal{N}(\mu)=\sum_\mu |V_1(f,\varphi_{1\mu})|^2\rho_{1\mu}\leqslant c_1.$$

Therefore, by dominated convergence theorem,

$$\lim_{\gamma_1\to 0}\sum_\mu |V_1(f,\varphi_{1\mu})|^2\frac{\gamma_1\rho_{1\mu}^2}{1+\gamma_1\rho_{1\mu}}=\lim_{\gamma_1\to 0}\int_{\mathbb{N}} f_{\gamma_1}^\dagger(\mu)\mathrm{d}\mathcal{N}(\mu)=\int_{\mathbb{N}}\lim_{\gamma_1\to 0} f_{\gamma_1}^\dagger(\mu)\mathrm{d}\mathcal{N}(\mu)=0,$$

which proves equation (S6). The other conclusion can be proved in a similar way. Thus, we complete the proof of Lemma S7.

Define the class of functions $\mathscr{G}_1=\{f\in\mathcal{H}_1:\|f\|_{\mathcal{H}_1}\leqslant 1\}$, $\mathscr{G}_0=\{g\in\mathcal{H}_0:\|g\|_{\mathcal{H}_0}\leqslant 1\}$. We state the following lemma.

Lemma S8: *Let $\boldsymbol{X}_i$, $i = 1, \ldots, n'$, be the i.i.d.samples of $\boldsymbol{X}$, and $\mathscr{H}$ be a Donsker class of functions which are uniformly bounded such that $\log N(\epsilon, \mathscr{H}, \|\cdot\|_\infty) \leqslant c_0\epsilon^{-v'}$ for some constants $c_0 > 0$ and $0 < v' < 2$. Suppose that Condition S5 holds, then*

$$\begin{aligned}
&\sup_{h\in\mathscr{H} f,\widetilde{f}\in\mathscr{G}_1} \left| \frac{1}{n'}\sum_{i=1}^{n'} h(\boldsymbol{X}_i)f(\boldsymbol{X}_i)\widetilde{f}(\boldsymbol{X}_i) - E\left\{\frac{1}{n'}\sum_{i=1}^{n'} h(\boldsymbol{X}_i)f(\boldsymbol{X}_i)\widetilde{f}(\boldsymbol{X}_i)\right\}\right| \\
= \;& O_P\left((n')^{-1/2}\left\{\gamma_1^{(-2m_1v'-4p+pv')/(8m_1)} + \gamma_1^{-(6m_1-p)p/(8m_1^2)}\right\}\right), \\
&\sup_{h\in\mathscr{H},g,\widetilde{g}\in\mathscr{G}_0} \left| \frac{1}{n'}\sum_{i=1}^{n'} h(\boldsymbol{X}_i)g(\boldsymbol{X}_i)\widetilde{g}(\boldsymbol{X}_i) - E\left\{\frac{1}{n'}\sum_{i=1}^{n'} h(\boldsymbol{X}_i)g(\boldsymbol{X}_i)\widetilde{g}(\boldsymbol{X}_i)\right\}\right| \\
= \;& O_P\left((n')^{-1/2}\left\{\gamma_0^{(-2m_0v'-4p+pv')/(8m_0)} + \gamma_0^{-(6m_0-p)p/(8m_0^2)}\right\}\right).
\end{aligned}$$

Proof. We first prove the first result. Define the class of functions

$$\mathscr{G}_1^* = \left\{f^* = \gamma_1^{1/2} f : f \in \mathcal{H}_1, \|f\|_\infty \leqslant \widetilde{c}_1\gamma_1^{-p/(4m_1)}, J_1(f,f) \leqslant \gamma_1^{-1}\right\},$$

then we have

$$\log N(\epsilon, \mathscr{G}_1^*, \|\cdot\|_\infty) \leqslant c_1\epsilon^{-p/m_1}.$$

We further define the class of functions

$$\mathscr{F} = \{F = hf^* : h \in \mathscr{H}, f^* \in \mathscr{G}_1^*\},$$

then

$$\log N(\epsilon, \mathscr{F}, \|\cdot\|_\infty) \leqslant c_2\left(\epsilon^{-v'} + \epsilon^{-p/m_1}\right). \tag{S7}$$

For $F \in \mathscr{F}$, define the empirical process $\mathcal{Z}_{n'}(F)$ as

$$\mathcal{Z}_{n'}(F) = \frac{1}{\sqrt{n'}}\sum_{i=1}^{n'}\left[\widetilde{c}_1^{-1}\gamma_1^{p/(4m_1)}F(\boldsymbol{X}_i)K_{1\boldsymbol{X}_i} - E\{\widetilde{c}_1^{-1}\gamma_1^{p/(4m_1)}F(\boldsymbol{X}_i)K_{1\boldsymbol{X}_i}\}\right].$$

For any $F, \widetilde{F} \in \mathscr{F}$, by Lemma S6,

$$\begin{aligned}
&\left\|\widetilde{c}_1^{-1}\,\gamma_1^{p/(4m_1)}F(\boldsymbol{X})K_{1\boldsymbol{X}} - \widetilde{c}_1^{-1}\gamma_1^{p/(4m_1)}\widetilde{F}(\boldsymbol{X})K_{1\boldsymbol{X}}\right\|_{\mathcal{H}_1} \\
\leqslant \;& \widetilde{c}_1^{-1}\gamma_1^{p/(4m_1)}\|F-\widetilde{F}\|_\infty\|K_{1\boldsymbol{X}}\|_{\mathcal{H}_1} \\
\leqslant \;& \widetilde{c}_1^{-1}\gamma_1^{p/(4m_1)}\|F-\widetilde{F}\|_\infty\widetilde{c}_1\gamma_1^{-p/(4m_1)} \\
= \;& \|F-\widetilde{F}\|_\infty,
\end{aligned}$$

coupled with Theorem 2 of Hoeffding (1963), entail that

$$P\left(\left\|\mathcal{Z}_{n'}(F)-\mathcal{Z}_{n'}(\widetilde{F})\right\|_{\mathcal{H}_1}\geqslant t\right)\leqslant 2\exp\left\{-\frac{t^2}{8\|F-\widetilde{F}\|_\infty^2}\right\}.$$

Together with Lemma 2.2.1 of van der Vaart and Wellner (1996),

$$\left\|\left\|\mathcal{Z}_{n'}(F)-\mathcal{Z}_{n'}(\widetilde{F})\right\|_{\mathcal{H}_1}\right\|_{\eta_2}\leqslant 8\left\|F-\widetilde{F}\right\|_\infty,$$

where η_2 is the Orlicz norm associated with $\eta_2(x)=\exp(x^2)-1$. Using Theorem 2.2.4 of van der Vaart and Wellner (1996) and equation (S7), for any $\delta>0$, we have

$$\begin{aligned}
&\left\|\sup_{F,\widetilde{F}\in\mathscr{F},\|F-\widetilde{F}\|_\infty\leqslant\delta}\left\|\mathcal{Z}_{n'}(F)-\mathcal{Z}_{n'}(\widetilde{F})\right\|_{\mathcal{H}_1}\right\|_{\eta_2}\\
\leqslant\ & c_3\left[\int_0^\delta\sqrt{\log\{1+N(\epsilon,\mathscr{F},\|\cdot\|_\infty)\}}\mathrm{d}\epsilon+\delta\sqrt{\log\{1+N(\delta,\mathscr{F},\|\cdot\|_\infty)^2\}}\right]\\
\leqslant\ & c_4\left\{\delta^{1-v'/2}+\delta^{1-p/(2m_1)}\right\}.
\end{aligned}$$

Then by Markov's inequality,

$$P\left(\sup_{F\in\mathscr{F},\|F\|_\infty\leqslant\delta}\|\mathcal{Z}_{n'}(F)\|_{\mathcal{H}_1}\geqslant t\right)\leqslant c_5\exp\left[-c_6\left\{\delta^{1-v'/2}+\delta^{1-p/(2m_1)}\right\}^{-2}t^2\right].$$

Set $\delta=\widetilde{c}_1\gamma_1^{1/2-p/(4m_1)}$, we get

$$P\left(\sup_{F\in\mathscr{F}}\|\mathcal{Z}_{n'}(F)\|_{\mathcal{H}_1}\geqslant t\right)\leqslant c_5\exp\left[-c_7\left\{\gamma_1^{(2m_1-p)(2-v')/(8m_1)}+\gamma_1^{(2m_1-p)^2/(8m_1^2)}\right\}^{-2}t^2\right],$$

which implies that

$$\sup_{F\in\mathscr{F}}\|\mathcal{Z}_{n'}(F)\|_{\mathcal{H}_1}=O_P\left(\gamma_1^{(2m_1-p)(2-v')/(8m_1)}+\gamma_1^{(2m_1-p)^2/(8m_1^2)}\right).$$

Noticing that $\gamma_1^{1/2}\mathscr{G}_1 = \{\gamma_1^{1/2} f : f \in \mathscr{G}_1\} \subset \mathscr{G}_1^*$, we obtain

$$\begin{aligned}
& \sup_{h\in\mathscr{H}, f, \widetilde{f}\in\mathscr{G}_1} \left| \frac{1}{n'}\sum_{i=1}^{n'} h(\boldsymbol{X}_i) f(\boldsymbol{X}_i)\widetilde{f}(\boldsymbol{X}_i) - E\left\{\frac{1}{n'}\sum_{i=1}^{n'} h(\boldsymbol{X}_i) f(\boldsymbol{X}_i)\widetilde{f}(\boldsymbol{X}_i)\right\}\right| \\
= & \sup_{h\in\mathscr{H}, f, \widetilde{f}\in\mathscr{G}_1} \left| \left\langle \frac{1}{n'}\sum_{i=1}^{n'} h(\boldsymbol{X}_i) f(\boldsymbol{X}_i) K_{1\boldsymbol{X}_i} - E\left\{\frac{1}{n'}\sum_{i=1}^{n'} h(\boldsymbol{X}_i) f(\boldsymbol{X}_i) K_{1\boldsymbol{X}_i}\right\}, \widetilde{f} \right\rangle_{\mathcal{H}_1} \right| \\
\leqslant & \sup_{h\in\mathscr{H}, f, \widetilde{f}\in\mathscr{G}_1} \left\| \frac{1}{n'}\sum_{i=1}^{n'} h(\boldsymbol{X}_i) f(\boldsymbol{X}_i) K_{1\boldsymbol{X}_i} - E\left\{\frac{1}{n'}\sum_{i=1}^{n'} h(\boldsymbol{X}_i) f(\boldsymbol{X}_i) K_{1\boldsymbol{X}_i}\right\} \right\|_{\mathcal{H}_1} \cdot \|\widetilde{f}\|_{\mathcal{H}_1} \\
\leqslant & (n')^{-1/2} \widetilde{c}_1 \gamma_1^{-1/2 - p/(4m_1)} \sup_{F\in\mathscr{F}} \|\mathscr{Z}_{n'}(F)\|_{\mathcal{H}_1} \\
= & O_P\left((n')^{-1/2} \left\{ \gamma_1^{(-2m_1 v' - 4p + pv')/(8m_1)} + \gamma_1^{-(6m_1 - p)p/(8m_1^2)} \right\} \right).
\end{aligned}$$

The remainder can be proved through a similar argument. Thus, we prove Lemma S8.

According to Condition S2, there exist some $\tau_n \in \Phi_n$ and $\lambda_n \in \Psi_n$ such that

$$\begin{aligned}
\|\tau_n - \tau_0\|_\infty &= O(n^{-\kappa_1}), \quad \sup_{|k|=m_1} \left\|\tau_n^{(k)} - \tau_0^{(k)}\right\|_\infty = O(n^{-\kappa_1'}), \\
\|\lambda_n - \lambda_0\|_\infty &= O(n^{-\kappa_0}), \quad \sup_{|k'|=m_0} \left\|\lambda_n^{(k')} - \lambda_0^{(k')}\right\|_\infty = O(n^{-\kappa_0'}).
\end{aligned} \tag{S8}$$

Then we assert the following lemma.

Lemma S9: *Suppose that the assumptions in Theorem 1 of the main paper hold, then $(\widehat{\tau}_n, \widehat{\lambda}_n)$, the minimum loss estimator of $\widehat{\ell}_{n,\gamma_1,\gamma_0}(\tau,\lambda)$ over $\Phi_n \times \Psi_n$, satisfies*

$$V_1(\widehat{\tau}_n - \tau_n, \widehat{\tau}_n - \tau_n) = o_P(1), \quad V_0(\widehat{\lambda}_n - \lambda_n, \widehat{\lambda}_n - \lambda_n) = o_P(1),$$

$$J_1(\widehat{\tau}_n - \tau_n, \widehat{\tau}_n - \tau_n) = o_P(1), \quad J_0(\widehat{\lambda}_n - \lambda_n, \widehat{\lambda}_n - \lambda_n) = o_P(1).$$

Proof. Choose $\xi_{1n} \in \Phi_n$ and $\xi_{0n} \in \Psi_n$ such that $(\xi_{1n}, \xi_{0n}) \in \mathcal{G}_1 \times \mathcal{G}_0$ with

$$\mathcal{G}_1 \times \mathcal{G}_0 = \left\{ (f,g) \in \mathcal{H} : c_1^{-1} \leqslant V_1(f,f) + V_0(g,g) \leqslant c_1,\ J_1(f,f) + J_0(g,g) \leqslant c_2 \right\}.$$

Moreover, by Sobolev embedding theorem, we have

$$\|\xi_{1n}\|_\infty + \|\xi_{0n}\|_\infty \leqslant c_3. \tag{S9}$$

For every $t \in \mathbb{R}$, let

$$\begin{aligned} H_n(t) &= \widehat{\ell}_{n,\gamma_1,\gamma_0}(\tau_n + t\xi_{1n}, \lambda_n + t\xi_{0n}) \\ &= \frac{1}{2n}\sum_{i=1}^{n} \left\{\widehat{\sigma}^2(\boldsymbol{X}_i, S_i)\right\}^{-1} \left[\widehat{D}_i - \{\tau_n(\boldsymbol{X}_i) + t\xi_{1n}(\boldsymbol{X}_i)\} - (1-S_i)\{\lambda_n(\boldsymbol{X}_i) + t\xi_{0n}(\boldsymbol{X}_i)\}\right]^2 \\ &\quad + \frac{\gamma_1}{2} J_1(\tau_n + t\xi_{1n}, \tau_n + t\xi_{1n}) + \frac{\gamma_0}{2} J_0(\lambda_n + t\xi_{0n}, \lambda_n + t\xi_{0n}), \end{aligned}$$

then the derivative of $H_n(t)$ with respect to t is

$$\begin{aligned} \dot{H}_n(t) &= -\frac{1}{n}\sum_{i=1}^{n} \left\{\widehat{\sigma}^2(\boldsymbol{X}_i, S_i)\right\}^{-1} \left[\widehat{D}_i - \{\tau_n(\boldsymbol{X}_i) + t\xi_n(\boldsymbol{X}_i)\} - (1-S_i)\{\lambda_n(\boldsymbol{X}_i) + t\eta_n(\boldsymbol{X}_i)\}\right] \\ &\quad \times\{\xi_{1n}(\boldsymbol{X}_i) + (1-S_i)\xi_{0n}(\boldsymbol{X}_i)\} \\ &\quad + t\gamma_1 J_1(\xi_{1n}, \xi_{1n}) + \gamma_1 J_1(\tau_n, \xi_{1n}) + t\gamma_0 J_0(\xi_{0n}, \xi_{0n}) + \gamma_0 J_0(\lambda_n, \xi_{0n}) \\ &= -\frac{1}{n}\sum_{i=1}^{n_1} \left\{\widehat{\sigma}^2(\boldsymbol{X}_i, 1)\right\}^{-1} (\widehat{D}_i - D_i)\xi_{1n}(\boldsymbol{X}_i) - \frac{1}{n}\sum_{i=1}^{n_1} \left\{\widehat{\sigma}^2(\boldsymbol{X}_i, 1)\right\}^{-1} \{D_i - \tau_n(\boldsymbol{X}_i)\}\,\xi_{1n}(\boldsymbol{X}_i) \\ &\quad + \frac{t}{n}\sum_{i=1}^{n_1} \left\{\widehat{\sigma}^2(\boldsymbol{X}_i, 1)\right\}^{-1} \xi_{1n}^2(\boldsymbol{X}_i) \\ &\quad - \frac{1}{n}\sum_{i=n_1+1}^{n} \left\{\widehat{\sigma}^2(\boldsymbol{X}_i, 0)\right\}^{-1} \{D_i - \tau_n(\boldsymbol{X}_i) - \lambda_n(\boldsymbol{X}_i)\}\{\xi_{1n}(\boldsymbol{X}_i) + \xi_{0n}(\boldsymbol{X}_i)\} \\ &\quad + \frac{t}{n}\sum_{i=n_1+1}^{n} \left\{\widehat{\sigma}^2(\boldsymbol{X}_i, 0)\right\}^{-1} \{\xi_{1n}(\boldsymbol{X}_i) + \xi_{0n}(\boldsymbol{X}_i)\}^2 + t\gamma_1 J_1(\xi_{1n}, \xi_{1n}) + \gamma_1 J_1(\tau_n, \xi_{1n}) \\ &\quad + t\gamma_0 J_0(\xi_{0n}, \xi_{0n}) + \gamma_0 J_0(\lambda_n, \xi_{0n}) \\ &= -I_{n1} - I_{n2} + tI_{n3} - I_{n4} + tI_{n5} + tI_{n6} + I_{n7} + tI_{n8} + I_{n9}, \end{aligned} \tag{S10}$$

where $I_{n1}, \ldots, I_{n9}$ are clear from the above equation.

We first consider I_{n2}. It follows that

$$\begin{aligned} |I_{n2}| &= \frac{n_1}{n}\left|\mathbb{P}_{n_1}\left\{(\widehat{\sigma}_1^2)^{-1}(D-\tau_n)\xi_{1n}\right\}\right| \\ &\leqslant \frac{n_1}{n}\left|\mathbb{P}_{n_1}\left\{(\widehat{\sigma}_1^2)^{-1}(D-\tau_0)\xi_{1n}\right\}\right| + \frac{n_1}{n}\left|\mathbb{P}_{n_1}\left\{(\widehat{\sigma}_1^2)^{-1}(\tau_n-\tau_0)\xi_{1n}\right\}\right| \\ &\leqslant \left|\mathbb{P}_{n_1}\left\{(\widehat{\sigma}_1^2)^{-1}(D-\tau_0)\xi_{1n}\right\}\right| + \left|\mathbb{P}_{n_1}\left\{(\widehat{\sigma}_1^2)^{-1}(\tau_n-\tau_0)\xi_{1n}\right\}\right|. \end{aligned}$$

Define the class of functions

$$\mathscr{F}^\dagger = \{(\widehat{\sigma}_1^2)^{-1}(D-\tau_0)\xi_{1n} : (\widehat{\sigma}_1^2)^{-1} \in \mathscr{A}, \xi_{1n} \in \mathcal{G}_1\}.$$

According to the definition of ξ_{1n}, it is easy to see that $\log N(\epsilon, \mathcal{G}_1, \|\cdot\|_\infty) \leqslant c_4 \epsilon^{-p/m_1}$. By Assumptions 1–2 in the main paper, Condition S3, and τ_0 is uniformly bounded,

$$\log N(\epsilon, \mathscr{F}^\dagger, \|\cdot\|_\infty) \leqslant c_5(\epsilon^{-\upsilon} + \epsilon^{-p/m_1}),$$

combined with the fact $m_1 > p/2$, i.e., $0 < p/m_1 < 2$, $0 < \upsilon < 2$ stated in Condition S3, and $\mathbb{P}_1\{(\widehat{\sigma}_1^2)^{-1}(D - \tau_0)\xi_{1n}\} = 0$, lead to

$$\begin{aligned} & \sup_{(\widehat{\sigma}_1^2)^{-1} \in \mathscr{A}, \xi_{1n} \in \mathcal{G}_1} \left| \mathbb{P}_{n_1}\left\{(\widehat{\sigma}_1^2)^{-1}(D - \tau_0)\xi_{1n}\right\}\right| \\ = & \sup_{(\widehat{\sigma}_1^2)^{-1} \in \mathscr{A}, \xi_{1n} \in \mathcal{G}_1} \left|(\mathbb{P}_{n_1} - \mathbb{P}_1)\left\{(\widehat{\sigma}_1^2)^{-1}(D - \tau_0)\xi_{1n}\right\}\right| \\ = & O_P\left(n_1^{-1/2}\right). \end{aligned} \tag{S11}$$

By equation (S8) and Condition S3,

$$\sup_{(\widehat{\sigma}_1^2)^{-1} \in \mathscr{A}, \xi_{1n} \in \mathcal{G}_1} \left|\mathbb{P}_{n_1}\left\{(\widehat{\sigma}_1^2)^{-1}(\tau_n - \tau_0)\xi_{1n}\right\}\right| \leqslant O(1)\|\tau_n - \tau_0\|_\infty \|\xi_{1n}\|_\infty = O\left(n^{-\kappa_1}\right).$$

Therefore

$$\begin{aligned} |I_{n2}| & \leqslant \sup_{(\widehat{\sigma}_1^2)^{-1} \in \mathscr{A}, \xi_{1n} \in \mathcal{G}_1} \left|\mathbb{P}_{n_1}\left\{(\widehat{\sigma}_1^2)^{-1}(D - \tau_0)\xi_{1n}\right\}\right| + \sup_{(\widehat{\sigma}_1^2)^{-1} \in \mathscr{A}, \xi_{1n} \in \mathcal{G}_1} \left|\mathbb{P}_{n_1}\left\{(\widehat{\sigma}_1^2)^{-1}(\tau_n - \tau_0)\xi_{1n}\right\}\right| \\ & = O_P\left(n^{-1/2}\right) + O\left(n^{-\kappa_1}\right) \\ & = o_P(1). \end{aligned} \tag{S12}$$

Mimicking the proof of (S12), we can also get

$$|I_{n4}| = o(1) + O\left(n^{-\kappa_1} + n^{-\kappa_0}\right) = o(1). \tag{S13}$$

Using an argument as we did in equation (S11), we have

$$\sup_{(\widehat{\sigma}_1^2)^{-1} \in \mathscr{A}, \xi_{1n} \in \mathcal{G}_1} \left|(\mathbb{P}_{n_1} - \mathbb{P}_1)(\widehat{\sigma}_1^2)^{-1}\xi_{1n}^2\right| = O_P\left(n_1^{-1/2}\right),$$

which implies that

$$\begin{aligned} I_{n3} & = \frac{n_1}{n}\left\{(\mathbb{P}_{n_1} - \mathbb{P}_1)(\widehat{\sigma}_1^2)^{-1}\xi_{1n}^2 + \mathbb{P}_1(\widehat{\sigma}_1^2)^{-1}\xi_{1n}^2\right\} \\ & = \frac{n_1}{n}\int \left\{\widehat{\sigma}_1^2(\boldsymbol{x})\right\}^{-1}\xi_{1n}^2(\boldsymbol{x})q_1(\boldsymbol{x})\mathrm{d}\boldsymbol{x} + o_P(1). \end{aligned} \tag{S14}$$

Similarly, we can also obtain

$$I_{n5} = \frac{n_2}{n} \int \left\{\widehat{\sigma}_0^2(\boldsymbol{x})\right\}^{-1} \{\xi_{1n}(\boldsymbol{x}) + \xi_{0n}(\boldsymbol{x})\}^2 q_0(\boldsymbol{x}) \mathrm{d}\boldsymbol{x} + o_P(1). \tag{S15}$$

It follows from equation (S8) that

$$\begin{aligned} I_{n6} &= o(1), \quad I_{n7} \leqslant \gamma_1 \{J_1(\tau_n, \tau_n)\}^{1/2} \{J_1(\xi_{1n}, \xi_{1n})\}^{1/2} = o(1), \\ I_{n8} &= o(1), \quad I_{n9} \leqslant \gamma_0 \{J_0(\lambda_n, \lambda_n)\}^{1/2} \{J_0(\xi_{0n}, \xi_{0n})\}^{1/2} = o(1). \end{aligned} \tag{S16}$$

We now deal with I_{n1}. It follows that

$$\begin{aligned} |I_{n1}| &= \left|\frac{n_1}{n} \mathbb{P}_{n_1} (\widehat{\sigma}_1^2)^{-1} (\widehat{D} - D) \xi_{1n}\right| \\ &\leqslant \left|\mathbb{P}_{n_1} (\widehat{\sigma}_1^2)^{-1} (\widehat{D} - D) \xi_{1n}\right| \\ &\leqslant \left|(\mathbb{P}_{n1} - \mathbb{P}_1)(\widehat{\sigma}_1^2)^{-1} (\widehat{D} - D) \xi_{1n}\right| + \left|\mathbb{P}_1 (\widehat{\sigma}_1^2)^{-1} (\widehat{D} - D) \xi_{1n}\right|. \end{aligned}$$

Using arguments similar to that in equation (S11), we get

$$\sup_{(\widehat{\sigma}_1^2)^{-1} \in \mathscr{A}, \widehat{D} \in \mathscr{D}, \xi_{1n} \in \mathcal{G}_1} \left|(\mathbb{P}_{n1} - \mathbb{P}_1)(\widehat{\sigma}_1^2)^{-1} (\widehat{D} - D) \xi_{1n}\right| = O_P\left(n_1^{-1/2}\right).$$

Under Conditions S3–S6, by Lemma S2, Hölder inequality and Minkowski inequality, we have

$$\begin{aligned} & \sup_{(\widehat{\sigma}_1^2)^{-1} \in \mathscr{A}, \widehat{D} \in \mathscr{D}, \xi_{1n} \in \mathcal{G}_1} \left|\mathbb{P}_1 \left(\widehat{\sigma}_1^2\right)^{-1} \left(\widehat{D} - D\right) \xi_{1n}\right| \\ = & \sup_{(\widehat{\sigma}_1^2)^{-1} \in \mathscr{A}, \widehat{D} \in \mathscr{D}, \xi_{1n} \in \mathcal{G}_1} \left|E_r \left[\left\{\widehat{\sigma}_1^2(\boldsymbol{X})\right\}^{-1} \xi_{1n}(\boldsymbol{X}) E_r \left\{\left(\widehat{D} - D\right) \,\middle|\, \boldsymbol{X}, S = 1\right\}\right]\right|. \\ = & \sup_{(\widehat{\sigma}_1^2)^{-1} \in \mathscr{A}, \xi_{1n} \in \mathcal{G}_1} \left|E_r \left[\left\{\widehat{\sigma}_1^2(\boldsymbol{X})\right\}^{-1} \xi_{1n}(\boldsymbol{X}) \left\{\Gamma_1(\boldsymbol{X}) + \Gamma_2(\boldsymbol{X})\right\}\right] \,\middle|\, S = 1\right| \\ \leqslant & O(1) \left(\sum_{a=0}^{1} \|\Theta_a\|_2 + \|\widehat{e} - e\|_2 \times \sum_{a=0}^{1} \|\widehat{\mu}_a - \mu_a\|_2\right) \\ = & o_P(1). \end{aligned}$$

As a consequence,

$$|I_{n1}| = o_P(1). \tag{S17}$$

Plugging (S12)–(S17) into (S10), we have

$$\begin{aligned}
& \dot{H}_n(t) \\
= \;& t\frac{n_1}{n}\int_\Omega \left\{\widehat{\sigma}_1^2(\boldsymbol{x})\right\}^{-1}\xi_{1n}^2(\boldsymbol{x})q_1(\boldsymbol{x})\mathrm{d}\boldsymbol{x} \\
& + t\frac{n_2}{n}\int_\Omega \left\{\widehat{\sigma}_0^2(\boldsymbol{x})\right\}^{-1}\{\xi_{1n}(\boldsymbol{x})+\xi_{0n}(\boldsymbol{x})\}^2 q_0(\boldsymbol{x})\mathrm{d}\boldsymbol{x} + o_P(1) \\
= \;& t\Bigg\{\varrho\int_\Omega \left\{\widehat{\sigma}_1^2(\boldsymbol{x})\right\}^{-1}\xi_{1n}^2(\boldsymbol{x})q_1(\boldsymbol{x})\mathrm{d}\boldsymbol{x} \\
& + (1-\varrho)\int_\Omega \left\{\widehat{\sigma}_0^2(\boldsymbol{x})\right\}^{-1}\{\xi_{1n}(\boldsymbol{x})+\xi_{0n}(\boldsymbol{x})\}^2 q_0(\boldsymbol{x})\mathrm{d}\boldsymbol{x}\Bigg\} + o_P(1).
\end{aligned}$$

By Lemma S3, Conditions S3 and S5,

$$\begin{aligned}
& \varrho\int \left\{\widehat{\sigma}_1^2(\boldsymbol{x})\right\}^{-1}\xi_{1n}^2(\boldsymbol{x})q_1(\boldsymbol{x})\mathrm{d}\boldsymbol{x} + (1-\varrho)\int \left\{\widehat{\sigma}_0^2(\boldsymbol{x})\right\}^{-1}\{\xi_{1n}(\boldsymbol{x})+\xi_{0n}(\boldsymbol{x})\}^2 q_0(\boldsymbol{x})\mathrm{d}\boldsymbol{x} \\
\geqslant \;& c_6\left\{V_1(\xi_{1n},\xi_{1n}) + V_0(\xi_{0n},\xi_{0n})\right\}.
\end{aligned}$$

Immediately, with probability tending to one,

$$\dot{H}_n(t) \geqslant tc_6\left\{V_1(\xi_{1n},\xi_{1n}) + V_0(\xi_{0n},\xi_{0n})\right\} \geqslant c_6c_1^{-1}t > 0$$

for $t > 0$. Similarly, we can also obtain that $\dot{H}_n(t) < 0$ for $t < 0$. In conclusion, $t\dot{H}_n(t) > 0$ for $t \neq 0$ with probability tending to one. By the arbitrariness of t, we conclude that

$$\begin{aligned}
V_1(\widehat{\tau}_n-\tau_n,\widehat{\tau}_n-\tau_n) = o_P(1), \quad V_0(\widehat{\lambda}_n-\lambda_n,\widehat{\lambda}_n-\lambda_n) = o_P(1), \\
J_1(\widehat{\tau}_n-\tau_n,\widehat{\tau}_n-\tau_n) = o_P(1), \quad J_0(\widehat{\lambda}_n-\lambda_n,\widehat{\lambda}_n-\lambda_n) = o_P(1),
\end{aligned}$$

which proves Lemma S9.

Web Appendix E: Proofs

E.1 Proof of Theorem 1

Proof. It follows from Lemma S9 and equation (S8) that

$$\begin{aligned} V_1(\widehat{\tau}_n-\tau_0,\widehat{\tau}_n-\tau_0) &\leqslant 2\big\{V_1(\widehat{\tau}_n-\tau_n,\widehat{\tau}_n-\tau_n)+V_1(\tau_n-\tau_0,\tau_n-\tau_0)\big\} \\ &= o_P(1)+O(n^{-2\kappa_1}) \\ &= o_P(1), \\ J_1(\widehat{\tau}_n-\tau_0,\widehat{\tau}_n-\tau_0) &\leqslant 2\big\{J_1(\widehat{\tau}_n-\tau_n,\widehat{\tau}_n-\tau_n)+J_1(\tau_n-\tau_0,\tau_n-\tau_0)\big\} \\ &= o_P(1)+O(n^{-2\kappa_1'}). \end{aligned}$$

Similarly, we can also get

$$V_0(\widehat{\lambda}_n-\lambda_0,\widehat{\lambda}_n-\lambda_0)=o_P(1),\quad J_0(\widehat{\lambda}_n-\lambda_0,\widehat{\lambda}_n-\lambda_0)=o_P(1)+O(n^{-2\kappa_0'}).$$

Immediately,

$$\begin{aligned} \|\widehat{\tau}_n-\tau_0\|_{\mathcal{H}_1} &= \Big\{V_1(\widehat{\tau}_n-\tau_0,\widehat{\tau}_n-\tau_0)+\gamma_1 J_1(\widehat{\tau}_n-\tau_0,\widehat{\tau}_n-\tau_0)\Big\}^{1/2}=o_P(1), \\ \Big\|\widehat{\lambda}_n-\lambda_0\Big\|_{\mathcal{H}_0} &= \Big\{V_0(\widehat{\lambda}_n-\lambda_0,\widehat{\lambda}_n-\lambda_0)+\gamma_0 J_0(\widehat{\lambda}_n-\lambda_0,\widehat{\lambda}_n-\lambda_0)\Big\}^{1/2}=o_P(1). \end{aligned}$$

Thus, we conclude Theorem 1.

E.2 Proof of Theorem 2

Proof. Taylor's expansion for $\widehat{\ell}_{n,\gamma_1,\gamma_0}(\widehat{\tau}_n,\widehat{\lambda}_n)$ around (τ_n,λ_n) yields

$$\begin{aligned} &\widehat{\ell}_{n,\gamma_1,\gamma_0}(\widehat{\tau}_n,\widehat{\lambda}_n)-\widehat{\ell}_{n,\gamma_1,\gamma_0}(\tau_n,\lambda_n) \\ &=\dot{\widehat{\ell}}_{n,\gamma_1,\gamma_0}(\tau_n,\lambda_n)[(\widehat{\tau}_n-\tau_n,\widehat{\lambda}_n-\lambda_n)] \\ &+\frac{1}{2}\ddot{\widehat{\ell}}_{n,\gamma_1,\gamma_0}(\tau_n,\lambda_n)[(\widehat{\tau}_n-\tau_n,\widehat{\lambda}_n-\lambda_n)][(\widehat{\tau}_n-\tau_n,\widehat{\lambda}_n-\lambda_n)] \\ &=I_{n1}^*+I_{n2}^*, \end{aligned}$$

where

$$\begin{aligned} I_{n1}^* &= \dot{\widehat{\ell}}_{n,\gamma_1,\gamma_0}(\tau_n,\lambda_n)[(\widehat{\tau}_n-\tau_n,\widehat{\lambda}_n-\lambda_n)] \\ &= -\frac{1}{n}\sum_{i=1}^{n}\left\{\widehat{\sigma}^2(\boldsymbol{X}_i,S_i)\right\}^{-1}\left\{\widehat{D}_i-\tau_n(\boldsymbol{X}_i)-(1-S_i)\lambda_n(\boldsymbol{X}_i)\right\} \\ &\quad \times\left[\{\widehat{\tau}_n(\boldsymbol{X}_i)-\tau_n(\boldsymbol{X}_i)\}+(1-S_i)\left\{\widehat{\lambda}_n(\boldsymbol{X}_i)-\lambda_n(\boldsymbol{X}_i)\right\}\right] \\ &\quad +\gamma_1 J_1(\tau_n,\widehat{\tau}_n-\tau_n)+\gamma_0 J_0(\lambda_n,\widehat{\lambda}_n-\lambda_n), \end{aligned}$$

and

$$\begin{aligned} I_{n2}^* &= \frac{1}{2}\ddot{\widehat{\ell}}_{n,\gamma_1,\gamma_0}(\tau_n,\lambda_n)[(\widehat{\tau}_n-\tau_n,\widehat{\lambda}_n-\lambda_n)][(\widehat{\tau}_n-\tau_n,\widehat{\lambda}_n-\lambda_n)] \\ &= \frac{1}{2n}\sum_{i=1}^{n}\left\{\widehat{\sigma}^2(\boldsymbol{X}_i,S_i)\right\}^{-1}\left[\{\widehat{\tau}_n(\boldsymbol{X}_i)-\tau_n(\boldsymbol{X}_i)\}+(1-S_i)\left\{\widehat{\lambda}_n(\boldsymbol{X}_i)-\lambda_n(\boldsymbol{X}_i)\right\}\right]^2 \\ &\quad +\frac{\gamma_1}{2}J_1(\widehat{\tau}_n-\tau_n,\widehat{\tau}_n-\tau_n)+\frac{\gamma_0}{2}J_0(\widehat{\lambda}_n-\lambda_n,\widehat{\lambda}_n-\lambda_n). \end{aligned}$$

We first consider I_{n1}^*. It follows that

$$\begin{aligned} -I_{n1}^* &= \frac{n_1}{n}\mathbb{P}_{n_1}(\widehat{\sigma}_1^2)^{-1}(\widehat{D}-D)(\widehat{\tau}_n-\tau_n)+\frac{n_1}{n}\mathbb{P}_{n_1}(\widehat{\sigma}_1^2)^{-1}(D-\tau_0)(\widehat{\tau}_n-\tau_n) \\ &\quad +\frac{n_0}{n}\mathbb{P}_{n_0}(\widehat{\sigma}_0^2)^{-1}(D-\tau_0-\lambda_0)(\widehat{\tau}_n-\tau_n)+\frac{n_0}{n}\mathbb{P}_{n_0}(\widehat{\sigma}_0^2)^{-1}(D-\tau_0-\lambda_0)(\widehat{\lambda}_n-\lambda_n) \\ &\quad +\frac{n_1}{n}\mathbb{P}_{n_1}(\widehat{\sigma}_1^2)^{-1}(\tau_0-\tau_n)(\widehat{\tau}_n-\tau_n)+\frac{n_0}{n}\mathbb{P}_{n_0}(\widehat{\sigma}_0^2)^{-1}(\tau_0-\tau_n)(\widehat{\tau}_n-\tau_n) \\ &\quad +\frac{n_0}{n}\mathbb{P}_{n_0}(\widehat{\sigma}_0^2)^{-1}(\tau_0-\tau_n)(\widehat{\lambda}_n-\lambda_n)+\frac{n_0}{n}\mathbb{P}_{n_0}(\widehat{\sigma}_0^2)^{-1}(\lambda_0-\lambda_n)(\widehat{\tau}_n-\tau_n) \\ &\quad +\frac{n_0}{n}\mathbb{P}_{n_0}(\widehat{\sigma}_0^2)^{-1}(\lambda_0-\lambda_n)(\widehat{\lambda}_n-\lambda_n)-\gamma_1 J_1(\tau_n,\widehat{\tau}_n-\tau_n)-\gamma_0 J_0(\lambda_n,\widehat{\lambda}_n-\lambda_n) \\ &= I_{n1,1}^*+I_{n1,2}^*+I_{n1,3}^*+I_{n1,4}^*+I_{n1,5}^*+I_{n1,6}^*+I_{n1,7}^*+I_{n1,8}^*+I_{n1,9}^* \\ &\quad -\gamma_1 J_1(\tau_n,\widehat{\tau}_n-\tau_n)-\gamma_0 J_0(\lambda_n,\widehat{\lambda}_n-\lambda_n), \end{aligned} \tag{S18}$$

where $I_{n1,1}^*,\ldots,I_{n1,9}^*$ are clear from the expression. Following equation (S8), it is clear that $J_1(\tau_n,\tau_n)\leqslant O(1)$, combined with Lemma S7, deduce that $\|W_{\gamma_1}\tau_n\|_{\mathcal{H}_1}=o(\gamma_1^{1/2})$. Consequently,

$$\begin{aligned} |\gamma_1 J_1(\tau_n,\widehat{\tau}_n-\tau_n)| = \left|\langle W_{\gamma_1}\tau_n,\widehat{\tau}_n-\tau_n\rangle_{\mathcal{H}_1}\right| &\leqslant \|W_{\gamma_1}\tau_n\|_{\mathcal{H}_1}\|\widehat{\tau}_n-\tau_n\|_{\mathcal{H}_1} \\ &= o(\gamma_1^{1/2})\|\widehat{\tau}_n-\tau_n\|_{\mathcal{H}_1}. \end{aligned} \tag{S19}$$

Similarly,

$$\left|\gamma_0 J_0(\lambda_n, \widehat{\lambda}_n - \lambda_n)\right| = o(\gamma_0^{1/2}) \left\|\widehat{\lambda}_n - \lambda_n\right\|_{\mathcal{H}_0}. \tag{S20}$$

For $I^*_{n1,1}$,

$$\begin{aligned} |I^*_{n1,1}| &\leqslant \left|\mathbb{P}_{n_1}(\widehat{\sigma}_1^2)^{-1}(\widehat{D} - D)(\widehat{\tau}_n - \tau_n)\right| \\ &\leqslant \left|(\mathbb{P}_{n_1} - \mathbb{P}_1)(\widehat{\sigma}_1^2)^{-1}(\widehat{D} - D)(\widehat{\tau}_n - \tau_n)\right| + \left|\mathbb{P}_1(\widehat{\sigma}_1^2)^{-1}(\widehat{D} - D)(\widehat{\tau}_n - \tau_n)\right|. \end{aligned}$$

Using a similar argument as we did in the proof of Lemma S8, we have

$$\begin{aligned} \left|(\mathbb{P}_{n_1} - \mathbb{P}_1)(\widehat{\sigma}_1^2)^{-1}(\widehat{D} - D)(\widehat{\tau}_n - \tau_n)\right| &= \left|\left\langle(\mathbb{P}_{n_1} - \mathbb{P}_1)(\widehat{\sigma}_1^2)^{-1}(\widehat{D} - D)K_1, \widehat{\tau}_n - \tau_n\right\rangle_{\mathcal{H}_1}\right| \\ &\leqslant \left\|(\mathbb{P}_{n_1} - \mathbb{P}_1)(\widehat{\sigma}_1^2)^{-1}(\widehat{D} - D)K_1\right\|_{\mathcal{H}_1} \cdot \|\widehat{\tau}_n - \tau_n\|_{\mathcal{H}_1} \\ &= O_P\left(n_1^{-1/2}\gamma_1^{-p/(4m_1)}\right) \|\widehat{\tau}_n - \tau_n\|_{\mathcal{H}_1} \\ &= O_P\left(n^{-1/2}\gamma_1^{-p/(4m_1)}\right) \|\widehat{\tau}_n - \tau_n\|_{\mathcal{H}_1}. \end{aligned}$$

In the following, we calculate $\mathbb{P}_1(\widehat{\sigma}_1^2)^{-1}(\widehat{D} - D)(\widehat{\tau}_n - \tau_n)$. Under Assumptions 1–2 in the main paper, Conditions S3–S5 ans S7, by Lemma S2, Hölder inequality and Minkowski inequality, we have

$$\begin{aligned} &\left|\mathbb{P}_1(\widehat{\sigma}_1^2)^{-1}(\widehat{D} - D)(\widehat{\tau}_n - \tau_n)\right| \\ = &\left|E_r\left[\{\widehat{\sigma}_1^2(\boldsymbol{X})\}^{-1}\{\widehat{\tau}_n(\boldsymbol{X}) - \tau_n(\boldsymbol{X})\}E_r\left\{(\widehat{D} - D) \mid \boldsymbol{X}, S = 1\right\}\right]\right|. \\ = &\left|E_r\left[\{\widehat{\sigma}_1^2(\boldsymbol{X})\}^{-1}\{\widehat{\tau}_n(\boldsymbol{X}) - \tau_n(\boldsymbol{X})\}\{\Gamma_1(\boldsymbol{X}) + \Gamma_2(\boldsymbol{X})\} \mid S = 1\right]\right| \\ \leqslant\; &O(1)\left(E_r\left[\{\widehat{\tau}_n(\boldsymbol{X}) - \tau_n(\boldsymbol{X})\}^2 \mid S = 1\right]\right)^{1/2}\left(\left[E_r\{\Gamma_1(\boldsymbol{X})\}^2\right]^{1/2} + \left[E_r\{\Gamma_2(\boldsymbol{X})\}^2\right]^{1/2}\right) \\ \leqslant\; &O(1)\|\widehat{\tau}_n - \tau_n\|_{\mathcal{H}_1}\left(\sum_{a=0}^{1}\|\Theta_a\|_2 + \|\widehat{e} - e\|_2 \times \sum_{a=0}^{1}\|\widehat{\mu}_a - \mu_a\|_2\right) \\ = &O_P(\delta_n)\|\widehat{\tau}_n - \tau_n\|_{\mathcal{H}_1}, \end{aligned}$$

where $\delta_n = n^{-1/2}\gamma_1^{-p/(4m_1)} + n^{-1/2}\gamma_0^{-p/(4m_0)} + \gamma_1^{1/2} + \gamma_0^{1/2}$. Therefore,

$$\begin{aligned} I^*_{n1,1} &= \left\{O_P\left(n^{-1/2}\gamma_1^{-p/(4m_1)}\right) + O_P(\delta_n)\right\}\|\widehat{\tau}_n - \tau_n\|_{\mathcal{H}_1} \\ &= O_P(\delta_n)\|\widehat{\tau}_n - \tau_n\|_{\mathcal{H}_1}. \end{aligned} \tag{S21}$$

A similar argument as in (S21) is used for $I^*_{n1,2}$, $I^*_{n1,3}$, and $I^*_{n1,4}$, we can get the similar results as follows.

$$\begin{aligned}
|I^*_{n1,2}| &= O_P\left(\delta_n\right)\|\widehat{\tau}_n-\tau_n\|_{\mathcal{H}_1}, \quad |I^*_{n1,3}| = O_P\left(\delta_n\right)\|\widehat{\tau}_n-\tau_n\|_{\mathcal{H}_1}, \\
|I^*_{n1,4}| &= O_P\left(\delta_n\right)\|\widehat{\lambda}_n-\lambda_n\|_{\mathcal{H}_0}. \qquad \text{(S22)}
\end{aligned}$$

We now consider $I^*_{n1,5}$. Mimicking the proof of Lemma S8, we have

$$\begin{aligned}
\frac{n}{n_1}\left|I^*_{n1,5}-E_r(I^*_{n1,5})\right| &= \left|(\mathbb{P}_{n_1}-\mathbb{P}_1)(\widehat{\sigma}_1^2)^{-1}(\tau_n-\tau_0)(\widehat{\tau}_n-\tau_n)\right| \\
&= |\left\langle(\mathbb{P}_{n_1}-\mathbb{P}_1)(\widehat{\sigma}_1^2)^{-1}(\tau_n-\tau_0)K_1,\widehat{\tau}_n-\tau_n\right\rangle_{\mathcal{H}_1}| \\
&\leqslant \|(\mathbb{P}_{n_1}-\mathbb{P}_1)(\widehat{\sigma}_1^2)^{-1}(\tau_n-\tau_0)K_1\|_{\mathcal{H}_1}\cdot\|\widehat{\tau}_n-\tau_n\|_{\mathcal{H}_1} \\
&\leqslant O_P\left(n_1^{-1/2}\gamma_1^{-p/(4m_1)}\|\tau_n-\tau_0\|_\infty^{1-p/(2m_1)}\right)\|\widehat{\tau}_n-\tau_n\|_{\mathcal{H}_1} \\
&\leqslant O_P\left(n^{-(1/2+\kappa_1(2m_1-p)/(2m_1))}\gamma_1^{-p/(4m_1)}\right)\|\widehat{\tau}_n-\tau_n\|_{\mathcal{H}_1}.
\end{aligned}$$

By equation (S8) and Condition S3,

$$\begin{aligned}
\frac{n}{n_1}|E_r(I^*_{n1,5})| &= \left|\int_\Omega\{\widehat{\sigma}_1^2(\boldsymbol{x})\}^{-1}\{\tau_n(\boldsymbol{x})-\tau_0(\boldsymbol{x})\}\left\{\widehat{\tau}_n(\boldsymbol{x})-\tau_n(\boldsymbol{x})\right\}q_1(\boldsymbol{x})\mathrm{d}\boldsymbol{x}\right| \\
&\leqslant O(1)\|\tau_n-\tau_0\|_\infty\|\widehat{\tau}_n-\tau_n\|_{\mathcal{H}_1} \\
&\leqslant O\left(n^{-\kappa_1}\right)\|\widehat{\tau}_n-\tau_n\|_{\mathcal{H}_1}.
\end{aligned}$$

Therefore,

$$\begin{aligned}
|I^*_{n1,5}| &\leqslant |I^*_{n1,5}-E_r(I^*_{n1,5})|+|E_r(I^*_{n1,5})| \\
&= O_P\left(n^{-(1/2+\kappa_1(2m_1-p)/(2m_1))}\gamma_1^{-p/(4m_1)}+n^{-\kappa_1}\right)\|\widehat{\tau}_n-\tau_n\|_{\mathcal{H}_1} \\
&= \left\{o_P\left(n^{-1/2}\gamma_1^{-p/(4m_1)}\right)+O_P\left(n^{-\kappa_1}\right)\right\}\|\widehat{\tau}_n-\tau_n\|_{\mathcal{H}_1}. \qquad \text{(S23)}
\end{aligned}$$

Using an argument similar to that in (S23) for $I^*_{n1,6}, \ldots, I^*_{n1,9}$, we get similar results as follows.

$$\begin{aligned}
I^*_{n1,6} &= \left\{o_P\left(n^{-1/2}\gamma_0^{-p/(4m_0)}\right) + O_P\left(n^{-\kappa_1}\right)\right\} \|\widehat{\tau}_n - \tau_n\|_{\mathcal{H}_1}, \\
I^*_{n1,7} &= \left\{o_P\left(n^{-1/2}\gamma_0^{-p/(4m_0)}\right) + O_P\left(n^{-\kappa_1}\right)\right\} \left\|\widehat{\lambda}_n - \lambda_n\right\|_{\mathcal{H}_0}, \\
I^*_{n1,8} &= \left\{o_P\left(n^{-1/2}\gamma_0^{-p/(4m_0)}\right) + O_P\left(n^{-\kappa_0}\right)\right\} \|\widehat{\tau}_n - \tau_n\|_{\mathcal{H}_1}, \\
I^*_{n1,9} &= \left\{o_P\left(n^{-1/2}\gamma_0^{-p/(4m_0)}\right) + O_P\left(n^{-\kappa_0}\right)\right\} \left\|\widehat{\lambda}_n - \lambda_n\right\|_{\mathcal{H}_0}. \qquad \text{(S24)}
\end{aligned}$$

Submitting (S21)–(S24) into (S18) and using Condition S9, we have

$$\begin{aligned}
|I^*_{n1}| &\leqslant O_P\left(\delta_n + n^{-\kappa_1} + n^{-\kappa_0}\right)\left(\|\widehat{\tau}_n - \tau_n\|_{\mathcal{H}_1} + \left\|\widehat{\lambda}_n - \lambda_n\right\|_{\mathcal{H}_0}\right) \\
&\leqslant O_P\left(\delta_n\right)\left(\|\widehat{\tau}_n - \tau_n\|_{\mathcal{H}_1} + \left\|\widehat{\lambda}_n - \lambda_n\right\|_{\mathcal{H}_0}\right). \qquad \text{(S25)}
\end{aligned}$$

We now deal with I^*_{n2}.

$$\begin{aligned}
& I^*_{n2} \\
= \ & \frac{n_1}{n}\mathbb{P}_{n_1}(\widehat{\sigma}_1^2)^{-1}(\widehat{\tau}_n - \tau_n)^2 + \frac{n_0}{n}\mathbb{P}_{n_0}(\widehat{\sigma}_0^2)^{-1}(\widehat{\tau}_n - \tau_n)^2 + \frac{n_0}{n}\mathbb{P}_{n_0}(\widehat{\sigma}_0^2)^{-1}(\widehat{\lambda}_n - \lambda_n)^2 \\
& +2\frac{n_0}{n}\mathbb{P}_{n_0}(\widehat{\sigma}_0^2)^{-1}(\widehat{\tau}_n - \tau_n)(\widehat{\lambda}_n - \lambda_n) + \gamma_1 J_1(\widehat{\tau}_n - \tau_n, \widehat{\tau}_n - \tau_n) \\
& +\gamma_0 J_0(\widehat{\lambda}_n - \lambda_n, \widehat{\lambda}_n - \lambda_n). \qquad \text{(S26)}
\end{aligned}$$

Following Lemma S8 and Condition S8, we have

$$\begin{aligned}
& \left|(\mathbb{P}_{n_1} - \mathbb{P}_1)(\widehat{\sigma}_1^2)^{-1}(\widehat{\tau}_n - \tau_n)^2\right| \\
\leqslant \ & \sup_{(\widehat{\sigma}_1^2)^{-1}\in\mathscr{A}, f, \widetilde{f}\in\mathscr{G}_1} \left|(\mathbb{P}_{n_1} - \mathbb{P}_1)(\widehat{\sigma}_1^2)^{-1} f\widetilde{f}\right| \|\widehat{\tau}_n - \tau_n\|^2_{\mathcal{H}_1}. \\
= \ & O_P\left(n^{-1/2}\left\{\gamma_1^{(-2m_1\upsilon - 4p + p\upsilon)/(8m_1)} + \gamma_1^{-(6m_1 - p)p/(8m_1^2)}\right\}\right)\|\widehat{\tau}_n - \tau_n\|^2_{\mathcal{H}_1} \\
= \ & o_P(1)\|\widehat{\tau}_n - \tau_n\|^2_{\mathcal{H}_1},
\end{aligned}$$

which implies that

$$\mathbb{P}_{n_1}(\widehat{\sigma}_1^2)^{-1}(\widehat{\tau}_n - \tau_n)^2 = \mathbb{P}_1(\widehat{\sigma}_1^2)^{-1}(\widehat{\tau}_n - \tau_n)^2 + o_P(1)\|\widehat{\tau}_n - \tau_n\|^2_{\mathcal{H}_1}. \qquad \text{(S27)}$$

Employing arguments analogous to equation (S27), we conclude

$$\begin{aligned}\mathbb{P}_{n_0}(\widehat{\sigma}_0^2)^{-1}(\widehat{\tau}_n-\tau_n)^2 &= \mathbb{P}_0(\widehat{\sigma}_0^2)^{-1}(\widehat{\tau}_n-\tau_n)^2+o_P(1)\|\widehat{\tau}_n-\tau_n\|^2_{\mathcal{H}_1},\\ \mathbb{P}_{n_0}(\widehat{\sigma}_0^2)^{-1}(\widehat{\lambda}_n-\lambda_n)^2 &= \mathbb{P}_0(\widehat{\sigma}_0^2)^{-1}(\widehat{\lambda}_n-\lambda_n)^2+o_P(1)\|\widehat{\lambda}_n-\lambda_n\|^2_{\mathcal{H}_0}. \qquad \text{(S28)}\end{aligned}$$

Now we consider $\mathbb{P}_{n_0}(\widehat{\sigma}_0^2)^{-1}(\widehat{\tau}_n-\tau_n)(\widehat{\lambda}_n-\lambda_n)$. Define the set $\mathcal{A}=\{V_0(\widehat{\lambda}_n-\lambda_n,\widehat{\lambda}_n-\lambda_n)+J_0(\widehat{\lambda}_n-\lambda_n,\widehat{\lambda}_n-\lambda_n)\leqslant 1\}$. For every $\epsilon>0$, according to Lemma S9, there exists some $N(\epsilon)$ such that $P(\mathcal{A})>1-\epsilon/2$ for all $n>N(\epsilon)$. Mimicking the proof of (S23), we further have

$$\begin{aligned}& P\left(\sup_{(\widehat{\sigma}_0^2)^{-1}\in\mathscr{A},V_0(\widehat{\lambda}_n-\lambda_n,\widehat{\lambda}_n-\lambda_n)+J_0(\widehat{\lambda}_n-\lambda_n,\widehat{\lambda}_n-\lambda_n)\leqslant 1}\left\|(\mathbb{P}_{n_0}-\mathbb{P}_0)(\widehat{\sigma}_0^2)^{-1}(\widehat{\lambda}_n-\lambda_n)K_1\right\|_{\mathcal{H}_1}>t\right)\\ \leqslant\ & c_1\exp(-c_2n_0\gamma_1^{p/(2m_1)}t^2),\end{aligned}$$

which implies that

$$\begin{aligned}& P\left(\sup_{(\widehat{\sigma}_0^2)^{-1}\in\mathscr{A},\widehat{\lambda}_n,\lambda_n}\left\|(\mathbb{P}_{n_0}-\mathbb{P}_0)(\widehat{\sigma}_0^2)^{-1}(\widehat{\lambda}_n-\lambda_n)K_1\right\|_{\mathcal{H}_1}>t\right)\\ \leqslant\ & P\left(\sup_{(\widehat{\sigma}_0^2)^{-1}\in\mathscr{A},\widehat{\lambda}_n,\lambda_n}\left\|(\mathbb{P}_{n_0}-\mathbb{P}_0)(\widehat{\sigma}_0^2)^{-1}(\widehat{\lambda}_n-\lambda_n)K_1\right\|_{\mathcal{H}_1}>t\,\middle|\,\mathcal{A}\right)+P(\mathcal{A}^C)\\ \leqslant\ & P\left(\sup_{(\widehat{\sigma}_0^2)^{-1}\in\mathscr{A},V_0(\widehat{\lambda}_n-\lambda_n,\widehat{\lambda}_n-\lambda_n)+J_0(\widehat{\lambda}_n-\lambda_n,\widehat{\lambda}_n-\lambda_n)\leqslant 1}\left\|(\mathbb{P}_{n_0}-\mathbb{P}_0)(\widehat{\sigma}_0^2)^{-1}(\widehat{\lambda}_n-\lambda_n)K_1\right\|_{\mathcal{H}_1}>t\right)\\ & +P(\mathcal{A}^C)\\ \leqslant\ & c_1\exp(-c_2n_0\gamma_1^{p/(2m_1)}t^2)+\epsilon/2\\ \leqslant\ & \epsilon\end{aligned}$$

by taking $t=\{c_2^{-1}\log(2c_1/\epsilon)n_0^{-1}\gamma_1^{-p/(2m_1)}\}^{1/2}$. Therefore,

$$\sup_{(\widehat{\sigma}_0^2)^{-1}\in\mathscr{A},\widehat{\lambda}_n,\lambda_n}\left\|(\mathbb{P}_{n_0}-\mathbb{P}_0)(\widehat{\sigma}_0^2)^{-1}(\widehat{\lambda}_n-\lambda_n)K_1\right\|_{\mathcal{H}_1}=O_P\left(n_0^{-1/2}\gamma_1^{-p/(4m_1)}\right)=O_P\left(n^{-1/2}\gamma_1^{-p/(4m_1)}\right),$$

furthermore,

$$\begin{aligned}\left|(\mathbb{P}_{n_0}-\mathbb{P}_0)(\widehat{\sigma}_0^2)^{-1}(\widehat{\tau}_n-\tau_n)(\widehat{\lambda}_n-\lambda_n)\right| &\leqslant \left\|(\mathbb{P}_{n_0}-\mathbb{P}_0)(\widehat{\sigma}_0^2)^{-1}(\widehat{\lambda}_n-\lambda_n)K_1\right\|_{\mathcal{H}_1}\|\widehat{\tau}_n-\tau_n\|_{\mathcal{H}_1}\\ &= O_P\left(n^{-1/2}\gamma_1^{-p/(4m_1)}\right)\|\widehat{\tau}_n-\tau_n\|_{\mathcal{H}_1}.\end{aligned}$$

Consequently,

$$\begin{aligned}
& \mathbb{P}_{n_0}(\widehat{\sigma}_0^2)^{-1}(\widehat{\tau}_n-\tau_n)(\widehat{\lambda}_n-\lambda_n) \\
= \; & \mathbb{P}_0(\widehat{\sigma}_0^2)^{-1}(\widehat{\tau}_n-\tau_n)(\widehat{\lambda}_n-\lambda_n)+O_P\left(n^{-1/2}\gamma_1^{-p/(4m_1)}\right)\|\widehat{\tau}_n-\tau_n\|_{\mathcal{H}_1}. \qquad \text{(S29)}
\end{aligned}$$

Plugging equations (S27)–(S29) into equation (S26), we conclude that

$$\begin{aligned}
I_{n2}^* = \; & \varrho\mathbb{P}_1(\widehat{\sigma}_1^2)^{-1}(\widehat{\tau}_n-\tau_n)^2+(1-\varrho)\mathbb{P}_0(\widehat{\sigma}_0^2)^{-1}(\widehat{\tau}_n-\tau_n)^2+(1-\varrho)\mathbb{P}_0(\widehat{\sigma}_0^2)^{-1}(\widehat{\lambda}_n-\lambda_n)^2 \\
& +2(1-\varrho)\mathbb{P}_0(\widehat{\sigma}_0^2)^{-1}(\widehat{\tau}_n-\tau_n)(\widehat{\lambda}_n-\lambda_n)+\gamma_1J_1(\widehat{\tau}_n-\tau_n,\widehat{\tau}_n-\tau_n)+\gamma_0J_0(\widehat{\lambda}_n-\lambda_n,\widehat{\lambda}_n-\lambda_n) \\
& +o_P(1)\|\widehat{\tau}_n-\tau_n\|_{\mathcal{H}_1}^2+o_P(1)\|\widehat{\lambda}_n-\lambda_n\|_{\mathcal{H}_0}^2+O_P\left(n^{-1/2}\gamma_1^{-p/(4m_1)}\right)\|\widehat{\tau}_n-\tau_n\|_{\mathcal{H}_1}.
\end{aligned}$$

It follows from Lemma S3, Conditions S3 and S5 that

$$\begin{aligned}
& \varrho\mathbb{P}_1(\widehat{\sigma}_1^2)^{-1}(\widehat{\tau}_n-\tau_n)^2+(1-\varrho)\mathbb{P}_0(\widehat{\sigma}_0^2)^{-1}(\widehat{\tau}_n-\tau_n)^2+(1-\varrho)\mathbb{P}_0(\widehat{\sigma}_0^2)^{-1}(\widehat{\lambda}_n-\lambda_n)^2 \\
& +2(1-\varrho)\mathbb{P}_0(\widehat{\sigma}_0^2)^{-1}(\widehat{\tau}_n-\tau_n)(\widehat{\lambda}_n-\lambda_n)+\gamma_1J_1(\widehat{\tau}_n-\tau_n,\widehat{\tau}_n-\tau_n)+\gamma_0J_0(\widehat{\lambda}_n-\lambda_n,\widehat{\lambda}_n-\lambda_n) \\
= \; & \varrho\mathbb{P}_1(\widehat{\sigma}_1^2)^{-1}(\widehat{\tau}_n-\tau_n)^2+(1-\varrho)\mathbb{P}_0(\widehat{\sigma}_0^2)^{-1}(\widehat{\tau}_n-\tau_n+\widehat{\lambda}_n-\lambda_n)^2 \\
& +\gamma_1J_1(\widehat{\tau}_n-\tau_n,\widehat{\tau}_n-\tau_n)+\gamma_0J_0(\widehat{\lambda}_n-\lambda_n,\widehat{\lambda}_n-\lambda_n) \\
\geqslant \; & c_3\left\{V_1(\widehat{\tau}_n-\tau_n,\widehat{\tau}_n-\tau_n)+V_0(\widehat{\lambda}_n-\lambda_n,\widehat{\lambda}_n-\lambda_n)\right\} \\
& +\gamma_1J_1(\widehat{\tau}_n-\tau_n,\widehat{\tau}_n-\tau_n)+\gamma_0J_0(\widehat{\lambda}_n-\lambda_n,\widehat{\lambda}_n-\lambda_n) \\
\geqslant \; & c_4\Big\{V_1(\widehat{\tau}_n-\tau_n,\widehat{\tau}_n-\tau_n)+V_0(\widehat{\lambda}_n-\lambda_n,\widehat{\lambda}_n-\lambda_n) \\
& +\gamma_1J_1(\widehat{\tau}_n-\tau_n,\widehat{\tau}_n-\tau_n)+\gamma_0J_0(\widehat{\lambda}_n-\lambda_n,\widehat{\lambda}_n-\lambda_n)\Big\} \\
= \; & c_4\left\{\|\widehat{\tau}_n-\tau_n\|_{\mathcal{H}_1}^2+\|\widehat{\lambda}_n-\lambda_n\|_{\mathcal{H}_0}^2\right\}
\end{aligned}$$

for some $0 < c_4 < 1$. Therefore,

$$I_{n2}^* \geqslant c_5\left\{\|\widehat{\tau}_n-\tau_n\|_{\mathcal{H}_1}^2+\|\widehat{\lambda}_n-\lambda_n\|_{\mathcal{H}_0}^2\right\}+O_P\left(n^{-1/2}\gamma_1^{-p/(4m_1)}\right)\|\widehat{\tau}_n-\tau_n\|_{\mathcal{H}_1}.$$

Recall that $(\widehat{\tau}_n,\widehat{\lambda}_n)$ is the minimizer of $\widehat{\ell}_{n,\gamma_1,\gamma_0}(\tau,\lambda)$ over $\Phi_n\times\Psi_n$, thus $\widehat{\ell}_{n,\gamma_1,\gamma_0}(\widehat{\tau}_n,\widehat{\lambda}_n)-$

$\widehat{\ell}_{n,\gamma_1,\gamma_0}(\tau_n, \lambda_n) \leqslant 0$, which implies that $I^*_{n2} \leqslant |I^*_{n1}|$, that is,

$$\begin{aligned} & c_5 \left\{ \|\widehat{\tau}_n - \tau_n\|^2_{\mathcal{H}_1} + \|\widehat{\lambda}_n - \lambda_n\|^2_{\mathcal{H}_0} \right\} + O_P\left(n^{-1/2} \gamma_1^{-p/(4m_1)} \right) \|\widehat{\tau}_n - \tau_n\|_{\mathcal{H}_1} \\ \leqslant \; & O_P\left(\delta_n\right) \left(\|\widehat{\tau}_n - \tau_n\|_{\mathcal{H}_1} + \|\widehat{\lambda}_n - \lambda_n\|_{\mathcal{H}_0} \right), \end{aligned}$$

which leads to

$$\|\widehat{\tau}_n - \tau_n\|_{\mathcal{H}_1} = O_P\left(\delta_n\right), \quad \|\widehat{\lambda}_n - \lambda_n\|_{\mathcal{H}_0} = O_P\left(\delta_n\right).$$

Equation (S8) and some routine calculations entail that

$$\|\tau_n - \tau_0\|_{\mathcal{H}_1} = O(n^{-\kappa_1} + \gamma_1^{1/2} n^{-\kappa_1'}), \quad \|\lambda_n - \lambda_0\|_{\mathcal{H}_0} = O(n^{-\kappa_0} + \gamma_0^{1/2} n^{-\kappa_0'}).$$

Therefore, the conclusion of Theorem 2 is deduced by

$$\begin{aligned} \|\widehat{\tau}_n - \tau_0\|_{\mathcal{H}_1} & \leqslant \|\widehat{\tau}_n - \tau_n\|_{\mathcal{H}_1} + \|\tau_n - \tau_0\|_{\mathcal{H}_1}, \\ \|\widehat{\lambda}_n - \lambda_0\|_{\mathcal{H}_0} & \leqslant \|\widehat{\lambda}_n - \lambda_n\|_{\mathcal{H}_0} + \|\lambda_n - \lambda_0\|_{\mathcal{H}_0}, \end{aligned}$$

and Condition S9.

E.3 Proof of Theorem 3

Proof. For $\boldsymbol{h}, \widetilde{\boldsymbol{h}} \in \mathcal{H}$, define the linear functional $\boldsymbol{h} : \mathcal{H} \to \mathbb{R}$ as $\boldsymbol{h}[\widetilde{\boldsymbol{h}}] = \langle \boldsymbol{h}, \widetilde{\boldsymbol{h}} \rangle_{\mathcal{H}}$. Set $\boldsymbol{\Upsilon} = (\tau, \lambda), \boldsymbol{\Upsilon}_0 = (\tau_0, \lambda_0), \widehat{\boldsymbol{\Upsilon}}_n = (\widehat{\tau}_n, \widehat{\lambda}_n)$. Let $\widehat{\boldsymbol{U}}_n(\boldsymbol{\Upsilon})$ and $\dot{\widehat{\boldsymbol{U}}}_n(\boldsymbol{\Upsilon})$ denote the first and second order Fréchet derivative operates of $\widehat{\ell}_n(\boldsymbol{\Upsilon})$ with respective to $\boldsymbol{\Upsilon}$, $\widehat{\boldsymbol{U}}_{n,\gamma_1,\gamma_0}(\boldsymbol{\Upsilon})$ and $\dot{\widehat{\boldsymbol{U}}}_{n,\gamma_1,\gamma_0}(\boldsymbol{\Upsilon})$ denote the first and second order Fréchet derivative operates of $\widehat{\ell}_{n,\gamma_1,\gamma_0}(\boldsymbol{\Upsilon})$ with respective to $\boldsymbol{\Upsilon}$, and $\boldsymbol{U}(\boldsymbol{\Upsilon})$ and $\dot{\boldsymbol{U}}(\boldsymbol{\Upsilon})$ denote the probability limit of $\widehat{\boldsymbol{U}}_n(\boldsymbol{\Upsilon})$ and $\dot{\widehat{\boldsymbol{U}}}_n(\boldsymbol{\Upsilon})$, respectively.

Then, for $\boldsymbol{h} = (f, g) \in \mathcal{H}, \widetilde{\boldsymbol{h}} = (\widetilde{f}, \widetilde{g}) \in \mathcal{H}$, we have

$$\begin{aligned}
\widehat{\boldsymbol{U}}_n(\boldsymbol{\Upsilon})[\boldsymbol{h}] &= -\frac{1}{n}\sum_{i=1}^{n}\left\{\widehat{\sigma}^2(\boldsymbol{X}_i, S_i)\right\}^{-1}\left\{\widehat{D}_i - \tau(\boldsymbol{X}_i) - (1-S_i)\lambda(\boldsymbol{X}_i)\right\} \\
&\quad \times\{f(\boldsymbol{X}_i) + (1-S_i)g(\boldsymbol{X}_i)\} \\
\dot{\widehat{\boldsymbol{U}}}_n(\boldsymbol{\Upsilon})[\boldsymbol{h}][\widetilde{\boldsymbol{h}}] &= \frac{1}{n}\sum_{i=1}^{n}\left\{\widehat{\sigma}^2(\boldsymbol{X}_i, S_i)\right\}^{-1} \\
&\quad \times\{f(\boldsymbol{X}_i) + (1-S_i)g(\boldsymbol{X}_i)\}\left\{\widetilde{f}(\boldsymbol{X}_i) + (1-S_i)\widetilde{g}(\boldsymbol{X}_i)\right\}, \\
\widehat{\boldsymbol{U}}_{n,\gamma_1,\gamma_0}(\boldsymbol{\Upsilon})[\boldsymbol{h}] &= \widehat{\boldsymbol{U}}_n(\boldsymbol{\Upsilon})[\boldsymbol{h}] + \boldsymbol{W}_{\boldsymbol{\gamma}}(\boldsymbol{\Upsilon})[\boldsymbol{h}], \\
\dot{\widehat{\boldsymbol{U}}}_{n,\gamma_1,\gamma_0}(\boldsymbol{\Upsilon})[\boldsymbol{h}][\widetilde{\boldsymbol{h}}] &= \dot{\widehat{\boldsymbol{U}}}_n(\boldsymbol{\Upsilon})[\boldsymbol{h}][\widetilde{\boldsymbol{h}}] + \dot{\boldsymbol{W}}_{\boldsymbol{\gamma}}(\boldsymbol{\Upsilon})[\boldsymbol{h}][\widetilde{\boldsymbol{h}}],
\end{aligned}$$

where $\boldsymbol{\gamma} = (\gamma_1, \gamma_0)$, $\boldsymbol{W}_{\boldsymbol{\gamma}}(\boldsymbol{\Upsilon}) = (W_{\gamma_1}\tau, W_{\gamma_0}\lambda)$ and $\dot{\boldsymbol{W}}_{\boldsymbol{\gamma}}(\boldsymbol{\Upsilon})[\boldsymbol{h}][\widetilde{\boldsymbol{h}}] = \gamma_1 J_1(f_\vartheta, \widetilde{f}_\vartheta) + \gamma_0 J_0(g_\vartheta, \widetilde{g}_\vartheta)$. Given $\boldsymbol{x}_0 \in \Omega$ and $\vartheta > 0$, define the class of functions

$$\mathscr{H}_\vartheta = \mathscr{H}_{1\vartheta} \times \mathscr{H}_{0\vartheta} = \left\{(f, g) \in \mathcal{H} : \left\|(f, g) - (\gamma_1^{p/(4m_1)} K_{1\boldsymbol{x}_0}, \gamma_0^{p/(4m_0)} K_{0\boldsymbol{x}_0})\right\|_{\mathcal{H}} \leqslant \vartheta\right\}$$

By Lemma S6, $\mathbb{P}_1 f_\vartheta^2 \leqslant \|f_\vartheta\|_{\mathcal{H}_1}^2/\varrho \leqslant (\vartheta + \widetilde{c}_1)^2/\varrho$ for all $f_\vartheta \in \mathscr{H}_{1\vartheta}$, thus $\mathscr{H}_{1\vartheta}$ is $\mathbb{P}_1$-Donsker. Similarly, $\mathscr{H}_{1\vartheta} + \mathscr{H}_{0\vartheta}$ is $\mathbb{P}_0$-Donsker.

The proof mainly consists of six steps.

Step 1. Show that

$$\sqrt{n}\widehat{\boldsymbol{U}}_n(\boldsymbol{\Upsilon}_0) \rightsquigarrow \boldsymbol{G} \quad \text{in } \ell^\infty(\mathscr{H}_\vartheta), \tag{S30}$$

where $\boldsymbol{G}[\boldsymbol{h}_\vartheta]$ is some zero-mean gaussian process with the covariance process

$$\operatorname{Cov}(\boldsymbol{G}[\boldsymbol{h}_\vartheta], \boldsymbol{G}[\boldsymbol{h}_\vartheta^\dagger]) = \varrho\mathbb{P}_1\left(\sigma_{01}^2\right)^{-1} f_\vartheta\widetilde{f}_\vartheta + (1-\varrho)\mathbb{P}_0\left(\sigma_{00}^2\right)^{-1}(f_\vartheta + g_\vartheta)(\widetilde{f}_\vartheta + \widetilde{g}_\vartheta),$$

and $\boldsymbol{h}_\vartheta = (f_\vartheta, g_\vartheta) \in \mathscr{H}_\vartheta, \widetilde{\boldsymbol{h}}_\vartheta = (\widetilde{f}_\vartheta, \widetilde{g}_\vartheta) \in \mathscr{H}_\vartheta$.

Step 2. Show that

$$\sqrt{n}\dot{\widehat{\boldsymbol{U}}}_n(\boldsymbol{\Upsilon}_0)[\widehat{\boldsymbol{\Upsilon}}_n - \boldsymbol{\Upsilon}_0] = \sqrt{n}\dot{\boldsymbol{U}}(\boldsymbol{\Upsilon}_0)[\widehat{\boldsymbol{\Upsilon}}_n - \boldsymbol{\Upsilon}_0] + o_P\left(1 + \sqrt{n}\left\|\widehat{\boldsymbol{\Upsilon}}_n - \boldsymbol{\Upsilon}_0\right\|_{\mathcal{H}}\right). \tag{S31}$$

Step 3. Show the invertibility of $\dot{\boldsymbol{U}}_{\boldsymbol{\gamma}}(\boldsymbol{\Upsilon}_0)$, where $\dot{\boldsymbol{U}}_{\boldsymbol{\gamma}}(\boldsymbol{\Upsilon}_0) = \dot{\boldsymbol{U}}(\boldsymbol{\Upsilon}_0) + \dot{\boldsymbol{W}}_{\boldsymbol{\gamma}}(\boldsymbol{\Upsilon}_0)$.

Step 4. Show that

$$\widehat{\boldsymbol{U}}_{n,\gamma_1,\gamma_0}(\widehat{\boldsymbol{\Upsilon}}_n)[\boldsymbol{h}_\vartheta] = o_P(n^{-1/2}), \tag{S32}$$

holds uniformly for $\boldsymbol{h}_\vartheta = (f_\vartheta, g_\vartheta) \in \mathscr{H}_\vartheta$.

Step 5. Steps 2–4 yield

$$\begin{aligned} &\sqrt{n}\dot{\boldsymbol{U}}_\gamma(\boldsymbol{\Upsilon}_0)\left[\widehat{\boldsymbol{\Upsilon}}_n - \boldsymbol{\Upsilon}_0 + \{\boldsymbol{U}'_\gamma(\boldsymbol{\Upsilon}_0)\}^{-1}[\boldsymbol{W}_\gamma(\boldsymbol{\Upsilon}_0)]\right] \\ = \;& -\sqrt{n}\widehat{\boldsymbol{U}}_n(\boldsymbol{\Upsilon}_0) + o_P\left(1 + \sqrt{n}\left\|\widehat{\boldsymbol{\Upsilon}}_n - \boldsymbol{\Upsilon}_0\right\|_{\mathcal{H}}\right), \end{aligned}$$

and further by Step 1,

$$\sqrt{n}\left(\widehat{\boldsymbol{\Upsilon}}_n - \boldsymbol{\Upsilon}_0 + \dot{\boldsymbol{U}}_\gamma^{-1}(\boldsymbol{\Upsilon}_0)[\boldsymbol{W}_\gamma(\boldsymbol{\Upsilon}_0)]\right) \rightsquigarrow \dot{\boldsymbol{U}}_\gamma^{-1}(\boldsymbol{\Upsilon}_0)[\boldsymbol{G}] \quad \text{in } \ell^\infty(\mathscr{H}_\vartheta). \tag{S33}$$

Step 6. For $\omega_1 \in \mathbb{R}$ and $\omega_0 \in \mathbb{R}$, let $\boldsymbol{h}_{\boldsymbol{\omega}} = (\omega_1\gamma_1^{p/(4m_1)}K_{1\boldsymbol{x}_0}, \omega_0\gamma_0^{p/(4m_0)}K_{0\boldsymbol{x}_0})$ and plug it into Step 5, we finally conclude Theorem 4.3.

We first prove Step 1. According to Condition S10, we have

$$E_r\left(\widehat{D} \mid \boldsymbol{X}, S=1\right) - E(D \mid \boldsymbol{X}, S=1) = o_P(n^{-1/2}) = o_P(1).$$

Additionally, by Condition S14, $E_r(\widehat{D} \mid \boldsymbol{X}, S=1) - E(D_0 \mid \boldsymbol{X}, S=1) = o_P(1)$. Thus, with probability one,

$$E(D_0 \mid \boldsymbol{X}, S=1) = E(D \mid \boldsymbol{X}, S=1) = \tau_0(\boldsymbol{X}). \tag{S34}$$

It follows that

$$\begin{aligned} & -\sqrt{n}\widehat{\boldsymbol{U}}_n(\boldsymbol{\Upsilon}_0)[\boldsymbol{h}_\vartheta] \\ = \;& \frac{n_1}{n}\sqrt{n}\mathbb{P}_{n_1}(\sigma_{01}^2)^{-1}(D_0 - \tau_0)f_\vartheta + \frac{n_0}{n}\sqrt{n}\mathbb{P}_{n_0}(\sigma_{00}^2)^{-1}(D - \tau_0 - \lambda_0)(f_\vartheta + g_\vartheta) \\ & + \frac{n_1}{n}\sqrt{n}\mathbb{P}_{n_1}(\widehat{\sigma}_1^2)^{-1}(\widehat{D} - D_0)f_\vartheta + \frac{n_1}{n}\sqrt{n}\mathbb{P}_{n_1}\left\{(\widehat{\sigma}_1^2)^{-1} - (\sigma_{01}^2)^{-1}\right\}(D - \tau_0)f_\vartheta \\ & + \frac{n_0}{n}\sqrt{n}\mathbb{P}_{n_0}\left\{(\widehat{\sigma}_0^2)^{-1} - (\sigma_{00}^2)^{-1}\right\}(D - \tau_0 - \lambda_0)(f_\vartheta + g_\vartheta). \end{aligned} \tag{S35}$$

By Conditions S3 and S13,

$$\mathbb{P}_1\left[\left\{(\widehat{\sigma}_1^2)^{-1} - (\sigma_{01}^2)^{-1}\right\}(D_0 - \tau_0)f_\vartheta\right]^2 \leqslant O(1)\left\|(\widehat{\sigma}_1^2)^{-1} - (\sigma_{01}^2)^{-1}\right\|_\infty^2 \mathbb{P}_1 f_\vartheta^2 = o_P(1),$$

combined with lemma 19.24 in van der Vaart (1998),

$$\sqrt{n_1}(\mathbb{P}_{n_1} - \mathbb{P}_1)\left\{(\widehat{\sigma}_1^2)^{-1} - (\sigma_{01}^2)^{-1}\right\}(D_0 - \tau_0)f_\vartheta = o_P(1).$$

Obviously, $\mathbb{P}_1\left\{(\widehat{\sigma}_1^2)^{-1} - (\sigma_{01}^2)^{-1}\right\}(D_0 - \tau_0)f_\vartheta = 0$ by equation (S34), then

$$\begin{aligned}
& \frac{n_1}{n}\sqrt{n}\mathbb{P}_{n_1}\left\{(\widehat{\sigma}_1^2)^{-1} - (\sigma_{01}^2)^{-1}\right\}(D_0 - \tau_0)f_\vartheta \\
= & \sqrt{\frac{n_1}{n}}\sqrt{n_1}(\mathbb{P}_{n_1} - \mathbb{P}_1)\left\{(\widehat{\sigma}_1^2)^{-1} - (\sigma_{01}^2)^{-1}\right\}(D - \tau_0)f_\vartheta \\
= & o_P(1). \qquad \text{(S36)}
\end{aligned}$$

Using an argument as we did in equation (S36), we can also get

$$\begin{aligned}
& \frac{n_0}{n}\sqrt{n}\mathbb{P}_{n_0}\left\{(\widehat{\sigma}_0^2)^{-1} - (\sigma_{00}^2)^{-1}\right\}(D - \tau_0 - \lambda_0)(f_\vartheta + g_\vartheta) \\
= & \sqrt{\frac{n_0}{n}}\sqrt{n_0}(\mathbb{P}_{n_0} - \mathbb{P}_0)\left\{(\widehat{\sigma}_0^2)^{-1} - (\sigma_{00}^2)^{-1}\right\}(D - \tau_0 - \lambda_0)(f_\vartheta + g_\vartheta) \\
= & o_P(1). \qquad \text{(S37)}
\end{aligned}$$

Now, we deal with the term $\sqrt{n}\mathbb{P}_{n_1}(\widehat{\sigma}_1^2)^{-1}(\widehat{D} - D_0)f_\vartheta$. Following Conditions S3 and S14, we have

$$\mathbb{P}_1\left\{(\widehat{\sigma}_1^2)^{-1}(\widehat{D} - D_0)f_\vartheta\right\}^2 \leqslant O(1)\|\widehat{D} - D_0\|_\infty^2\mathbb{P}_1 f_\vartheta^2 \leqslant o_P(1)\mathbb{P}_1 f_\vartheta^2 = o_P(1),$$

coupled with lemma 19.24 in van der Vaart (1998), lead to

$$\sqrt{n_1}(\mathbb{P}_{n_1} - \mathbb{P}_1)(\widehat{\sigma}_1^2)^{-1}(\widehat{D} - D_0)f_\vartheta = o_P(1). \qquad \text{(S38)}$$

Furthermore, by equation (S34) and Lemma S2,

$$E_r\left(\widehat{D} - D_0 \mid \boldsymbol{X}, S = 1\right) = E_r\left(\widehat{D} - D \mid \boldsymbol{X}, S = 1\right) = \Gamma_1(\boldsymbol{X}) + \Gamma_2(\boldsymbol{X}),$$

which implies that

$$\begin{aligned}\left|\mathbb{P}_1(\widehat{\sigma}_1^2)^{-1}(\widehat{D}-D_0)f_\vartheta\right| &= \left|\int_\Omega \left\{\widehat{\sigma}_1^2(\boldsymbol{x})\right\}^{-1}\left\{\Gamma_1(\boldsymbol{x})+\Gamma_2(\boldsymbol{x})\right\}f_\vartheta(\boldsymbol{x})q_1(\boldsymbol{x})\mathrm{d}\boldsymbol{x}\right| \\ &\leqslant O(1)\left[\int_\Omega\{\Gamma_1(\boldsymbol{x})+\Gamma_2(\boldsymbol{x})\}^2 q_1(\boldsymbol{x})\mathrm{d}x\right]^{1/2}\left[\int_\Omega\{f_\vartheta(\boldsymbol{x})\}^2 q_1(\boldsymbol{x})\mathrm{d}x\right]^{1/2} \\ &\leqslant O(1)\left(\sum_{a=0}^{1}\|\Theta_a\|_2+\|\widehat{e}-e\|_2\times\sum_{a=0}^{1}\|\widehat{\mu}_a-\mu_a\|_2\right) \\ &= o_P(n^{-1/2}),\end{aligned}$$

by using Conditions S3 and S10. Therefore,

$$\begin{aligned}\sqrt{n}\mathbb{P}_{n_1}(\widehat{\sigma}_1^2)^{-1}(\widehat{D}-D_0)f_\vartheta &= \sqrt{n}\mathbb{P}_1(\widehat{\sigma}_1^2)^{-1}(\widehat{D}-D_0)f_\vartheta+o_P(1) \\ &= \sqrt{n}o_P(n^{-1/2})+o_P(1) \\ &= o_P(1). \qquad \text{(S39)}\end{aligned}$$

Plugging equations (S36)–(S39) into equation (S35), we obtain

$$\begin{aligned}&-\sqrt{n}\widehat{\boldsymbol{U}}_n(\boldsymbol{\Upsilon}_0)[\boldsymbol{h}_\vartheta] \\ =\ & \frac{n_1}{n}\sqrt{n}\mathbb{P}_{n_1}(\sigma_{01}^2)^{-1}(D_0-\tau_0)f_\vartheta+\frac{n_0}{n}\sqrt{n}\mathbb{P}_{n_0}(\sigma_{00}^2)^{-1}(D-\tau_0-\lambda_0)(f_\vartheta+g_\vartheta)+o_P(1) \\ =\ & \sqrt{\frac{n_1}{n}}\sqrt{n_1}(\mathbb{P}_{n_1}-\mathbb{P}_1)(\sigma_{01}^2)^{-1}(D_0-\tau_0)f_\vartheta \\ & +\sqrt{\frac{n_0}{n}}\sqrt{n_0}(\mathbb{P}_{n_0}-\mathbb{P}_0)(\sigma_{00}^2)^{-1}(D-\tau_0-\lambda_0)(f_\vartheta+g_\vartheta)+o_P(1).\end{aligned}$$

According to Assumptions 1–2 in the main paper and Condition S3, the class of functions $\{(\sigma_{01}^2)^{-1}(D_0-\tau_0)f_\vartheta : f_\vartheta\in\mathscr{H}_{1\vartheta}\}$ is $\mathbb{P}_1$-Donsker, and $\{(\sigma_{00}^2)^{-1}(D-\tau_0-\lambda_0)(f_\vartheta+g_\vartheta) : \boldsymbol{h}_\vartheta=(f_\vartheta,g_\vartheta)\in\mathscr{H}_\vartheta\}$ is $\mathbb{P}_0$-Donsker. Consequently, $\sqrt{n}\widehat{\boldsymbol{U}}_n(\boldsymbol{\Upsilon}_0)[\boldsymbol{h}_\vartheta]$ converges to $\boldsymbol{G}[\boldsymbol{h}_\vartheta]$ in distribution. Therefore,

$$\sqrt{n}\widehat{\boldsymbol{U}}_n(\boldsymbol{\Upsilon}_0)\rightsquigarrow\boldsymbol{G}\quad\text{in } \ell^\infty(\mathscr{H}_\vartheta),$$

which proves Step 1.

We next prove Step 2. For $\boldsymbol{h}_\vartheta = (f_\vartheta, g_\vartheta) \in \mathscr{H}_\vartheta$,

$$\begin{aligned}
\dot{\widehat{\boldsymbol{U}}}_n(\boldsymbol{\Upsilon}_0)[\widehat{\boldsymbol{\Upsilon}}_n - \boldsymbol{\Upsilon}_0][\boldsymbol{h}_\vartheta] &= \frac{n_1}{n}\mathbb{P}_{n_1}\left(\widehat{\sigma}_1^2\right)^{-1}(\widehat{\tau}_n - \tau_0)f_\vartheta \\
&\quad + \frac{n_0}{n}\mathbb{P}_{n_0}\left(\widehat{\sigma}_0^2\right)^{-1}(\widehat{\tau}_n - \tau_0 + \widehat{\lambda}_n - \lambda_0)(f_\vartheta + g_\vartheta), \\
\dot{\boldsymbol{U}}(\boldsymbol{\Upsilon}_0)[\widehat{\boldsymbol{\Upsilon}}_n - \boldsymbol{\Upsilon}_0][\boldsymbol{h}_\vartheta] &= \varrho\mathbb{P}_1\left(\sigma_{01}^2\right)^{-1}(\widehat{\tau}_n - \tau_0)f_\vartheta \\
&\quad + (1-\varrho)\mathbb{P}_0\left(\sigma_{00}^2\right)^{-1}(\widehat{\tau}_n - \tau_0 + \widehat{\lambda}_n - \lambda_0)(f_\vartheta + g_\vartheta). \quad \text{(S40)}
\end{aligned}$$

For every $\epsilon_1 > 0$, define the set $\mathcal{B}_1 = \{\|\widehat{\tau}_n - \tau_0\|_\infty \leqslant \epsilon_1^{1/2}\}$ and $\mathcal{B}_2 = \{V_1(\tau_n - \tau_0, \tau_n - \tau_0) + J_1(\tau_n - \tau_0, \tau_n - \tau_0) \leqslant 1\}$. According to Theorem 4.1, Lemma S6 and Condition S11, we have $\|\widehat{\tau}_n - \tau_0\|_\infty \leqslant \widetilde{c}_1\gamma_1^{-p/(4m_1)}\|\widehat{\tau}_n - \tau_0\|_{\mathcal{H}_1} = O_P(\gamma_1^{-p/(4m_1)}\delta_n) = o_P(1)$, which implies that, for every $\epsilon_2 > 0$, there exists some $N_1(\epsilon_2)$ such that $P(\mathcal{B}_1) > 1 - \epsilon_2/3$ for all $n > N_1(\epsilon_2)$. According to Lemma S9, equation (S8) and Condition S12, there exists some $N_2(\epsilon_1, \epsilon_2)$ such that $P(\mathcal{B}_2) > 1 - \epsilon_2/3$ for all $n > N_2(\epsilon_1, \epsilon_2)$. Thus, for every $\epsilon_1 > 0$ and $\epsilon_2 > 0$, there exists some $N_3(\epsilon_1, \epsilon_2) = \max\{N_1(\epsilon_2), N_2(\epsilon_1, \epsilon_2)\}$ such that

$$P\left((\mathcal{B}_1\mathcal{B}_2)^C\right) \leqslant P(\mathcal{B}_1^C) + P(\mathcal{B}_2^C) \leqslant 2\epsilon_2/3$$

for all $n > N_3(\epsilon_1, \epsilon_2)$. Additionally, on $\mathcal{B}_1\mathcal{B}_2$, by Condition S3,

$$\mathbb{P}_1\left\{\left(\widehat{\sigma}_1^2\right)^{-1}(\widehat{\tau}_n - \tau_0)f_\vartheta\right\}^2 \leqslant c_1\|\widehat{\tau}_n - \tau_0\|_\infty^2\mathbb{P}_1 f_\vartheta^2 \leqslant c_2\|\widehat{\tau}_n - \tau_0\|_\infty^2 \leqslant c_2\epsilon_1,$$

combined with lemma 19.24 in van der Vaart (1998), entails that, there exists some $N_4(\epsilon_1, \epsilon_2)$ such that

$$P\left(\sqrt{n_1}\left|(\mathbb{P}_{n_1} - \mathbb{P}_1)\left(\widehat{\sigma}_1^2\right)^{-1}(\widehat{\tau}_n - \tau_0)f_\vartheta\right| > \epsilon_1\right) \leqslant \epsilon_2/3.$$

for all $n > N_4(\epsilon_1, \epsilon_2)$. Consequently, for every $\epsilon_1 > 0$ and $\epsilon_2 > 0$, there exists some $N(\epsilon_1, \epsilon_2) = \max\{N_3(\epsilon_1, \epsilon_2), N_4(\epsilon_1, \epsilon_2)\}$ such that

$$\begin{aligned}
& P\left(\sqrt{n_1}\left|(\mathbb{P}_{n_1} - \mathbb{P}_1)\left(\widehat{\sigma}_1^2\right)^{-1}(\widehat{\tau}_n - \tau_0)f_\vartheta\right| > \epsilon_1\right) \\
\leqslant\ & P\left(\sqrt{n_1}\left|(\mathbb{P}_{n_1} - \mathbb{P}_1)\left(\widehat{\sigma}_1^2\right)^{-1}(\widehat{\tau}_n - \tau_0)f_\vartheta\right| > \epsilon_1\middle|\mathcal{B}_1\mathcal{B}_2\right) + P\left((\mathcal{B}_1\mathcal{B}_2)^C\right) \\
\leqslant\ & \epsilon_2.
\end{aligned}$$

Immediately,

$$\sqrt{n_1}\left|(\mathbb{P}_{n_1}-\mathbb{P}_1)\left(\widehat{\sigma}_1^2\right)^{-1}(\widehat{\tau}_n-\tau_0)f_\vartheta\right| = o_P(1). \tag{S41}$$

Next, by Condition S13 and Hölder inequality,

$$\begin{aligned}\left|\mathbb{P}_1\left\{\left(\widehat{\sigma}_1^2\right)^{-1}-\left(\sigma_{01}^2\right)^{-1}\right\}(\widehat{\tau}_n-\tau_0)f_\vartheta\right| &\leqslant \left\|\left(\widehat{\sigma}_1^2\right)^{-1}-\left(\sigma_{01}^2\right)^{-1}\right\|_\infty \mathbb{P}_1\left|(\widehat{\tau}_n-\tau_0)f_\vartheta\right| \\ &\leqslant o_P(1)\left\{\mathbb{P}_1(\widehat{\tau}_n-\tau_0)^2\right\}^{1/2}\left(\mathbb{P}_1 f_\vartheta^2\right)^{1/2} \\ &\leqslant o_P(1)\|\widehat{\tau}_n-\tau_0\|_{\mathcal{H}_1} \\ &\leqslant o_P(1)\left\|\widehat{\boldsymbol{\Upsilon}}_n-\boldsymbol{\Upsilon}_0\right\|_{\mathcal{H}}, \\ \left|\mathbb{P}_1\left(\sigma_{01}^2\right)^{-1}(\widehat{\tau}_n-\tau_0)f_\vartheta\right| &\leqslant O(1)\left\|\widehat{\boldsymbol{\Upsilon}}_n-\boldsymbol{\Upsilon}_0\right\|_{\mathcal{H}}. \end{aligned} \tag{S42}$$

Thus

$$\begin{aligned} & \sqrt{n}\mathbb{P}_{n_1}\left(\widehat{\sigma}_1^2\right)^{-1}(\widehat{\tau}_n-\tau_0)f_\vartheta \\ = {} & \sqrt{n}\mathbb{P}_1\left(\widehat{\sigma}_1^2\right)^{-1}(\widehat{\tau}_n-\tau_0)f_\vartheta + o_P(1) \\ = {} & \sqrt{n}\mathbb{P}_1\left(\sigma_{01}^2\right)^{-1}(\widehat{\tau}_n-\tau_0)f_\vartheta + \sqrt{n}\mathbb{P}_1\left\{\left(\widehat{\sigma}_1^2\right)^{-1}-\left(\sigma_{01}^2\right)^{-1}\right\}(\widehat{\tau}_n-\tau_0)f_\vartheta + o_P(1) \\ = {} & \sqrt{n}\mathbb{P}_1\left(\sigma_{01}^2\right)^{-1}(\widehat{\tau}_n-\tau_0)f_\vartheta + \sqrt{n}o_P(1)\left\|\widehat{\boldsymbol{\Upsilon}}_n-\boldsymbol{\Upsilon}_0\right\|_{\mathcal{H}} + o_P(1) \\ = {} & \sqrt{n}\mathbb{P}_1\left(\sigma_{01}^2\right)^{-1}(\widehat{\tau}_n-\tau_0)f_\vartheta + o_P\left(1+\sqrt{n}\left\|\widehat{\boldsymbol{\Upsilon}}_n-\boldsymbol{\Upsilon}_0\right\|_{\mathcal{H}}\right). \end{aligned} \tag{S43}$$

By similar arguments that used in equation (S43),

$$\begin{aligned} & \sqrt{n}\mathbb{P}_{n_0}\left(\widehat{\sigma}_0^2\right)^{-1}(\widehat{\tau}_n-\tau_0+\widehat{\lambda}_n-\lambda_0)(f_\vartheta+g_\vartheta) \\ = {} & \sqrt{n}\mathbb{P}_0\left(\sigma_{00}^2\right)^{-1}(\widehat{\tau}_n-\tau_0+\widehat{\lambda}_n-\lambda_0)f_\vartheta + o_P\left(1+\sqrt{n}\left\|\widehat{\boldsymbol{\Upsilon}}_n-\boldsymbol{\Upsilon}_0\right\|_{\mathcal{H}}\right). \end{aligned} \tag{S44}$$

Combining equations (S40)–(S44), we have

$$\begin{aligned}
& \left|\sqrt{n}\dot{\widehat{\boldsymbol{U}}}_n(\boldsymbol{\Upsilon}_0)[\widehat{\boldsymbol{\Upsilon}}_n-\boldsymbol{\Upsilon}_0][\boldsymbol{h}_\vartheta]-\sqrt{n}\dot{\boldsymbol{U}}(\boldsymbol{\Upsilon}_0)[\widehat{\boldsymbol{\Upsilon}}_n-\boldsymbol{\Upsilon}_0][\boldsymbol{h}_\vartheta]\right| \\
\leqslant\ & \frac{n_1}{n}\left|\sqrt{n}\mathbb{P}_{n_1}\left(\widehat{\sigma}_1^2\right)^{-1}(\widehat{\tau}_n-\tau_0)f_\vartheta-\sqrt{n}\mathbb{P}_1\left(\sigma_{01}^2\right)^{-1}(\widehat{\tau}_n-\tau_0)f_\vartheta\right| \\
& +\frac{n_0}{n}\left|\sqrt{n}\mathbb{P}_{n_0}\left(\widehat{\sigma}_0^2\right)^{-1}(\widehat{\tau}_n-\tau_0+\widehat{\lambda}_n-\lambda_0)(f_\vartheta+g_\vartheta)-\sqrt{n}\mathbb{P}_0\left(\sigma_{00}^2\right)^{-1}(\widehat{\tau}_n-\tau_0+\widehat{\lambda}_n-\lambda_0)f_\vartheta\right| \\
& +\left|\frac{n_1}{n}-\rho\right|\left|\sqrt{n}\mathbb{P}_1\left(\sigma_{01}^2\right)^{-1}(\widehat{\tau}_n-\tau_0)f_\vartheta\right| \\
& +\left|\frac{n_0}{n}-(1-\rho)\right|\left|\sqrt{n}\mathbb{P}_0\left(\sigma_{00}^2\right)^{-1}(\widehat{\tau}_n-\tau_0+\widehat{\lambda}-\lambda_0)(f_\vartheta+g_\vartheta)\right| \\
\leqslant\ & o_P\left(1+\sqrt{n}\left\|\widehat{\boldsymbol{\Upsilon}}_n-\boldsymbol{\Upsilon}_0\right\|_{\mathcal{H}}\right),
\end{aligned}$$

holds uniformly for $\boldsymbol{h}_\vartheta\in\mathscr{H}_\vartheta$. Immediately,

$$\sqrt{n}\dot{\widehat{\boldsymbol{U}}}_n(\boldsymbol{\Upsilon}_0)[\widehat{\boldsymbol{\Upsilon}}_n-\boldsymbol{\Upsilon}_0]=\sqrt{n}\dot{\boldsymbol{U}}(\boldsymbol{\Upsilon}_0)[\widehat{\boldsymbol{\Upsilon}}_n-\boldsymbol{\Upsilon}_0]+o_P\left(1+\sqrt{n}\left\|\widehat{\boldsymbol{\Upsilon}}_n-\boldsymbol{\Upsilon}_0\right\|_{\mathcal{H}}\right),$$

which proves Step 2.

We now show Step 3. It follows from Lemma S3 that

$$\begin{aligned}
& \dot{\boldsymbol{U}}_{\boldsymbol{\gamma}}(\boldsymbol{\Upsilon}_0)[\boldsymbol{h}_\vartheta][\boldsymbol{h}_\vartheta] \\
=\ & \varrho\mathbb{P}_1\left(\sigma_{01}^2\right)^{-1}f_\vartheta^2+(1-\varrho)\mathbb{P}_0\left(\sigma_{00}^2\right)^{-1}(f_\vartheta+g_\vartheta)^2+\gamma_1J_1(f_\vartheta,f_\vartheta)+\gamma_0J_0(g_\vartheta,g_\vartheta) \\
\geqslant\ & c_0\{V_1(f_\vartheta,f_\vartheta)+V_0(g_\vartheta,g_\vartheta)\}+\gamma_1J_1(f_\vartheta,f_\vartheta)+\gamma_0J_0(g_\vartheta,g_\vartheta) \\
\geqslant\ & c_1\left\{\|f_\vartheta\|_{\mathcal{H}_1}^2+\|g_\vartheta\|_{\mathcal{H}_0}^2\right\} \\
=\ & c_1\|\boldsymbol{h}_\vartheta\|_{\mathcal{H}}^2,
\end{aligned}$$

for every $\boldsymbol{h}_\vartheta\in\mathscr{H}_\vartheta$ and $\boldsymbol{h}_\vartheta\neq(0,0)$, which shows the invertibility of $\dot{\boldsymbol{U}}_{\boldsymbol{\gamma}}(\boldsymbol{\Upsilon}_0)$.

We further prove Step 4. According to Lemma S6, we have $J_1(\gamma_1^{1/2}f_\vartheta,\gamma_1^{1/2}f_\vartheta)\leqslant O(1)$, $\|\gamma_1^{1/2}f_\vartheta\|_\infty\leqslant O(1)$, $J_0(\gamma_0^{1/2}g_\vartheta,\gamma_0^{1/2}g_\vartheta)\leqslant O(1)$, and $\|\gamma_0^{1/2}g_\vartheta\|_\infty\leqslant O(1)$, combined with Condition S2, there exists some $\boldsymbol{h}_{n\vartheta}=(f_{n\vartheta},g_{n\vartheta})\in\Phi_n\times\Psi_n$ such that

$$\begin{aligned}
&\left\|\gamma_1^{1/2}f_{n\vartheta}-\gamma_1^{1/2}f_\vartheta\right\|_\infty=O(n^{-\kappa_1}),\quad \sup_{|k|=m_1}\left\|\gamma_1^{1/2}f_{n\vartheta}^{(k)}-\gamma_1^{1/2}f_\vartheta^{(k)}\right\|_\infty=O(n^{-\kappa_1'}),\\
&\left\|\gamma_0^{1/2}g_{n\vartheta}-\gamma_0^{1/2}g_\vartheta\right\|_\infty=O(n^{-\kappa_0}),\quad \sup_{|k'|=m_0}\left\|\gamma_0^{1/2}g_{n\vartheta}^{(k')}-\gamma_0^{1/2}g_\vartheta^{(k')}\right\|_\infty=O(n^{-\kappa_0'}).
\end{aligned}\tag{S45}$$

Note that $\widehat{\boldsymbol{\Upsilon}}_n$ is the minimizer of $\widehat{\ell}_{n,\gamma_1,\gamma_0}(\boldsymbol{\Upsilon})$ over $\Psi_n\times\Phi_n$. Thus, for any $\boldsymbol{h}_n=(f_n,g_n)\in$

$\Phi_n \times \Psi_n$, $\widehat{\boldsymbol{U}}_{n,\gamma_1,\gamma_0}(\widehat{\boldsymbol{\Upsilon}}_n)[\boldsymbol{h}_n] = 0$, which implies that

$$\widehat{\boldsymbol{U}}_{n,\gamma_1,\gamma_0}(\widehat{\boldsymbol{\Upsilon}}_n)[\boldsymbol{h}_{n\vartheta}] = 0.$$

Thus

$$\begin{aligned}
& \widehat{\boldsymbol{U}}_{n,\gamma_1,\gamma_0}(\widehat{\boldsymbol{\Upsilon}}_n)[\boldsymbol{h}_\vartheta] \\
= \; & \widehat{\boldsymbol{U}}_{n,\gamma_1,\gamma_0}(\widehat{\boldsymbol{\Upsilon}}_n)[\boldsymbol{h}_\vartheta] - \widehat{\boldsymbol{U}}_{n,\gamma_1,\gamma_0}(\widehat{\boldsymbol{\Upsilon}}_n)[\boldsymbol{h}_{n\vartheta}] \\
= \; & \frac{n_1}{n}\mathbb{P}_{n_1}\left(\widehat{\sigma}_1^2\right)^{-1}(\widehat{D} - \widehat{\tau}_n)(f_{n\vartheta} - f_\vartheta) + \frac{n_0}{n}\mathbb{P}_{n_0}\left(\widehat{\sigma}_0^2\right)^{-1}(D - \widehat{\tau}_n - \widehat{\lambda}_n)(f_{n\vartheta} - f_\vartheta + g_{n\vartheta} - g_\vartheta) \\
& + \gamma_1 J_1(\widehat{\tau}_n, f_\vartheta - f_{n\vartheta}) + \gamma_0 J_0(\widehat{\lambda}_n, g_\vartheta - g_{n\vartheta}) \\
= \; & \frac{n_1}{n}\mathbb{P}_{n_1}\left(\widehat{\sigma}_1^2\right)^{-1}(\widehat{D} - D_0)(f_{n\vartheta} - f_\vartheta) + \frac{n_1}{n}\mathbb{P}_{n_1}\left(\widehat{\sigma}_1^2\right)^{-1}(D_0 - \widehat{\tau}_n)(f_{n\vartheta} - f_\vartheta) \\
& + \frac{n_0}{n}\mathbb{P}_{n_0}\left(\widehat{\sigma}_0^2\right)^{-1}(D - \widehat{\tau}_n - \widehat{\lambda}_n)(f_{n\vartheta} - f_\vartheta + g_{n\vartheta} - g_\vartheta) \\
& + \gamma_1 J_1(\widehat{\tau}_n, f_\vartheta - f_{n\vartheta}) + \gamma_0 J_0(\widehat{\lambda}_n, g_\vartheta - g_{n\vartheta}) \\
= \; & \frac{n_1}{n} I_{n1}^\dagger + \frac{n_1}{n} I_{n2}^\dagger + \frac{n_0}{n} I_{n3}^\dagger + I_{n4}^\dagger + I_{n5}^\dagger, \qquad \text{(S46)}
\end{aligned}$$

where $I_{n1}^\dagger, \ldots, I_{n5}^\dagger$ are self-explained from the above equation. By Conditions S3 and S14, and equation (S45),

$$\mathbb{P}_1\left\{\left(\widehat{\sigma}_1^2\right)^{-1}(\widehat{D} - D_0)(f_{n\vartheta} - f_\vartheta)\right\}^2 \leqslant O(1)\|\widehat{D} - D_0\|_\infty^2 \|f_{n\vartheta} - f_\vartheta\|_\infty^2 = o(1),$$

combined with lemma 19.24 in van der Vaart (1998), yield

$$\sqrt{n_1}(\mathbb{P}_{n_1} - \mathbb{P}_1)\left(\widehat{\sigma}_1^2\right)^{-1}(\widehat{D} - D_0)(f_{n\vartheta} - f_\vartheta) = o_P(1). \qquad \text{(S47)}$$

Using analogous arguments that used in equations (S47) and (S41), we have

$$\begin{aligned}
\sqrt{n_1}(\mathbb{P}_{n_1} - \mathbb{P}_1)\left(\widehat{\sigma}_1^2\right)^{-1}(D_0 - \tau_0)(f_{n\vartheta} - f_\vartheta) &= o_P(1), \\
\sqrt{n_1}(\mathbb{P}_{n_1} - \mathbb{P}_1)\left(\widehat{\sigma}_1^2\right)^{-1}(\widehat{\tau}_n - \tau_0)(f_{n\vartheta} - f_\vartheta) &= o_P(1).
\end{aligned}$$

Therefore,

$$
\begin{aligned}
& \sqrt{n_1}\left|(\mathbb{P}_{n_1}-\mathbb{P}_1)\left(\widehat{\sigma}_1^2\right)^{-1}(D_0-\widehat{\tau}_n)(f_{n\vartheta}-f_\vartheta)\right| \\
\leqslant\ & \sqrt{n_1}\left|(\mathbb{P}_{n_1}-\mathbb{P}_1)\left(\widehat{\sigma}_1^2\right)^{-1}(D_0-\tau_0)(f_{n\vartheta}-f_\vartheta)\right| \\
& +\sqrt{n_1}\left|(\mathbb{P}_{n_1}-\mathbb{P}_1)\left(\widehat{\sigma}_1^2\right)^{-1}(\widehat{\tau}_n-\tau_0)(f_{n\vartheta}-f_\vartheta)\right| \\
=\ & o_P(1). \qquad \text{(S48)}
\end{aligned}
$$

Similarly, we can also derive

$$
\sqrt{n_0}(\mathbb{P}_{n_0}-\mathbb{P}_0)\left(\widehat{\sigma}_0^2\right)^{-1}(D-\widehat{\tau}_n-\widehat{\lambda}_n)(f_{n\vartheta}-f_\vartheta+g_{n\vartheta}-g_\vartheta)=o_P(1). \qquad \text{(S49)}
$$

Under Conditions S3 and S12, combining equations (S47)– (S49) and (S45), we have

$$
\begin{aligned}
|I_{n1}^{\dagger}| &= \left|(\mathbb{P}_{n_1}-\mathbb{P}_1)\left(\widehat{\sigma}_1^2\right)^{-1}(\widehat{D}-D_0)(f_{n\vartheta}-f_\vartheta)+\mathbb{P}_1\left(\widehat{\sigma}_1^2\right)^{-1}(\widehat{D}-D_0)(f_{n\vartheta}-f_\vartheta)\right| \\
&\leqslant \left|\mathbb{P}_1\left(\widehat{\sigma}_1^2\right)^{-1}(\widehat{D}-D_0)(f_{n\vartheta}-f_\vartheta)\right|+o_P(n^{-1/2}) \\
&\leqslant \|f_{n\vartheta}-f_\vartheta\|_\infty\mathbb{P}_1|\Gamma_1+\Gamma_2|+o_P(n^{-1/2}) \\
&= o_P(n^{-1/2})+O\left(n^{-\kappa_1}\gamma_1^{-1/2}\right)\left(\sum_{a=0}^{1}\|\Theta_a\|_2+\|\widehat{e}-e\|_2\times\sum_{a=0}^{1}\|\widehat{\mu}_a-\mu_a\|_2\right) \\
&= o_P(n^{-1/2})+O_P(n^{-\kappa_1}\gamma_1^{-1/2}n^{-1/2}) \\
&= o_P(n^{-1/2}) \qquad \text{(S50)}
\end{aligned}
$$

by Condition S10, and

$$
\begin{aligned}
|I_{n2}^{\dagger}| &= \left|(\mathbb{P}_{n_1} - \mathbb{P}_1)\left(\widehat{\sigma}_1^2\right)^{-1}(D_0 - \widehat{\tau}_n)(f_{n\vartheta} - f_{\vartheta}) + \mathbb{P}_1\left(\widehat{\sigma}_1^2\right)^{-1}(D_0 - \widehat{\tau}_n)(f_{n\vartheta} - f_{\vartheta})\right| \\
&\leqslant \left|\mathbb{P}_1\left(\widehat{\sigma}_1^2\right)^{-1}(D_0 - \widehat{\tau}_n)(f_{n\vartheta} - f_{\vartheta})\right| + o_P(n^{-1/2}) \\
&= \left|E_r\left[\left\{\widehat{\sigma}_1^2(\boldsymbol{X})\right\}^{-1} E_r\{(D_0 - \widehat{\tau}_n) \mid \boldsymbol{X}, S = 1\}\{f_{n\vartheta}(\boldsymbol{X}) - f_{\vartheta}(\boldsymbol{X})\}\right]\right| + o_P(n^{-1/2}) \\
&\leqslant O(1)\{\mathbb{P}_1(\tau_0 - \widehat{\tau}_n)^2\}^{1/2}\{\mathbb{P}_1(f_{n\vartheta} - f_{\vartheta})^2\}^{1/2} + o_P(n^{-1/2}) \\
&\leqslant O\left(n^{-\kappa_1}\gamma_1^{-1/2}\right)\|\widehat{\tau}_n - \tau_0\|_{\mathcal{H}_1} + o_P(n^{-1/2}) \\
&= O_P\left(n^{-\kappa_1}\gamma_1^{-1/2}\delta_n\right) + o_P(n^{-1/2}) \\
&= o_P(n^{-1/2}), \\
|I_{n3}^{\dagger}| &= \Big|(\mathbb{P}_{n_0} - \mathbb{P}_0)\left(\widehat{\sigma}_0^2\right)^{-1}(D - \widehat{\tau}_n - \widehat{\lambda}_n)(f_{n\vartheta} - f_{\vartheta} + g_{n\vartheta} - g_{\vartheta}) \\
&\quad + \mathbb{P}_0\left(\widehat{\sigma}_0^2\right)^{-1}(D - \widehat{\tau}_n - \widehat{\lambda}_n)(f_{n\vartheta} - f_{\vartheta} + g_{n\vartheta} - g_{\vartheta})\Big| \\
&\leqslant \left|\mathbb{P}_0\left(\widehat{\sigma}_0^2\right)^{-1}(D - \widehat{\tau}_n - \widehat{\lambda}_n)(f_{n\vartheta} - f_{\vartheta} + g_{n\vartheta} - g_{\vartheta})\right| + o_P(n^{-1/2}) \\
&\leqslant O(1)\left\{\mathbb{P}_0(\tau_0 - \widehat{\tau}_n)^2 + \mathbb{P}_0(\lambda_0 - \widehat{\lambda}_n)^2\right\}^{1/2}\{\mathbb{P}_0(f_{n\vartheta} - f_{\vartheta})^2 + \mathbb{P}_0(g_{n\vartheta} - g_{\vartheta})^2\}^{1/2} \\
&\quad + o_P(n^{-1/2}) \\
&= O_P\left((n^{-\kappa_1}\gamma_1^{-1/2} + n^{-\kappa_0}\gamma_0^{-1/2})\delta_n\right) + o_P(n^{-1/2}) \\
&= o_P(n^{-1/2}), \qquad \text{(S51)}
\end{aligned}
$$

by Theorem 4.2.

Next, we deal with the terms $I_{n4}^{\dagger}$ and $I_{n5}^{\dagger}$. By Lemma S7, equation (S45), Theorem 4.2 and

Condition S12,

$$
\begin{aligned}
|I_{n4}^{\dagger}| &= |\langle W_{\gamma_1}\widehat{\tau}_n, f_\vartheta - f_{n\vartheta}\rangle_{\mathcal{H}_1}| \\
&\leqslant |\langle W_{\gamma_1}(\widehat{\tau}_n - \tau_0), f_\vartheta - f_{n\vartheta}\rangle_{\mathcal{H}_1}| + |\langle W_{\gamma_1}\tau_0, f_\vartheta - f_{n\vartheta}\rangle_{\mathcal{H}_1}| \\
&\leqslant |\langle W_{\gamma_1}(f_\vartheta - f_{n\vartheta}), \widehat{\tau}_n - \tau_0\rangle_{\mathcal{H}_1}| + \|W_{\gamma_1}\tau_0\|_{\mathcal{H}_1}\|f_\vartheta - f_{n\vartheta}\|_{\mathcal{H}_1} \\
&\leqslant n^{-\kappa_1'}\gamma_1^{-1/2}\|W_{\gamma_1}n^{\kappa_1'}\gamma_1^{1/2}(f_\vartheta - f_{n\vartheta})\|_{\mathcal{H}_1}\|\widehat{\tau}_n - \tau_0\|_{\mathcal{H}_1} + o\left(n^{-\kappa_1} + n^{-\kappa_1'}\gamma_1^{1/2}\right) \\
&\leqslant n^{-\kappa_1'}\gamma_1^{-1/2}o(\gamma_1^{1/2})\delta_n + o\left(n^{-\kappa_1} + n^{-\kappa_1'}\gamma_1^{1/2}\right) \\
&= o\left(n^{-\kappa_1'}\delta_n + n^{-\kappa_1}\right) \\
&= o_P(n^{-1/2}). \qquad \text{(S52)}
\end{aligned}
$$

In a manner similar to equation (S52), we can also conclude

$$|I_{n5}^{\dagger}| = o_P(n^{-1/2}). \qquad \text{(S53)}$$

Plugging equations (S50)–(S53) into equation (S46), we conclude

$$\widehat{\boldsymbol{U}}_{n,\gamma_1,\gamma_0}(\widehat{\boldsymbol{\Upsilon}}_n)[\boldsymbol{h}_\vartheta] = o_P(n^{-1/2}),$$

which proves Step 4.

Next, we move on to Step 5. It follows that

$$
\begin{aligned}
&\widehat{\boldsymbol{U}}_{n,\gamma_1,\gamma_0}(\widehat{\boldsymbol{\Upsilon}}_n)[\boldsymbol{h}_\vartheta] - \widehat{\boldsymbol{U}}_{n,\gamma_1,\gamma_0}(\boldsymbol{\Upsilon}_0)[\boldsymbol{h}_\vartheta] \\
= \; &\dot{\widehat{\boldsymbol{U}}}_{n,\gamma_1,\gamma_0}(\boldsymbol{\Upsilon}_0)[\widehat{\boldsymbol{\Upsilon}}_n - \boldsymbol{\Upsilon}_0][\boldsymbol{h}_\vartheta] \\
= \; &\dot{\widehat{\boldsymbol{U}}}_n(\boldsymbol{\Upsilon}_0)[\widehat{\boldsymbol{\Upsilon}}_n - \boldsymbol{\Upsilon}_0][\boldsymbol{h}_\vartheta] + \gamma_1 J_1(\widehat{\tau}_n - \tau_0, f_\vartheta) + \gamma_0 J_0(\widehat{\lambda}_n - \lambda_0, g_\vartheta),
\end{aligned}
$$

combined with equations (S31) and (S32), we have

$$
\begin{aligned}
\sqrt{n}\dot{\boldsymbol{U}}_\gamma(\boldsymbol{\Upsilon}_0)[\widehat{\boldsymbol{\Upsilon}}_n - \boldsymbol{\Upsilon}_0] &= -\sqrt{n}\widehat{\boldsymbol{U}}_n(\boldsymbol{\Upsilon}_0) - \sqrt{n}\boldsymbol{W}_\gamma(\boldsymbol{\Upsilon}_0) \\
&\quad + o_P\left(1 + \sqrt{n}\left\|\widehat{\boldsymbol{\Upsilon}}_n - \boldsymbol{\Upsilon}_0\right\|_{\mathcal{H}}\right)
\end{aligned}
$$

Noting that the operate $\dot{\boldsymbol{U}}_{\gamma}(\boldsymbol{\Upsilon}_0)$ is reversible as stated in Step 3, we obtain

$$\begin{aligned} & \sqrt{n}\dot{\boldsymbol{U}}_{\gamma}(\boldsymbol{\Upsilon}_0)\left[\widehat{\boldsymbol{\Upsilon}}_n - \boldsymbol{\Upsilon}_0 + \dot{\boldsymbol{U}}_{\gamma}^{-1}(\boldsymbol{\Upsilon}_0)[\boldsymbol{W}_{\gamma}(\boldsymbol{\Upsilon}_0)]\right] \\ = \ & -\sqrt{n}\widehat{\boldsymbol{U}}_n(\boldsymbol{\Upsilon}_0) + o_P\left(1+\sqrt{n}\left\|\widehat{\boldsymbol{\Upsilon}}_n - \boldsymbol{\Upsilon}_0\right\|_{\mathcal{H}}\right). \end{aligned}$$

By equation (S30) and theorem 3.1 in van der Vaart and Wellner (1996), we finally conclude that

$$\sqrt{n}\left(\widehat{\boldsymbol{\Upsilon}}_n - \boldsymbol{\Upsilon}_0 + \dot{\boldsymbol{U}}_{\gamma}^{-1}(\boldsymbol{\Upsilon}_0)[\boldsymbol{W}_{\gamma}(\boldsymbol{\Upsilon}_0)]\right) \rightsquigarrow \dot{\boldsymbol{U}}_{\gamma}^{-1}(\boldsymbol{\Upsilon}_0)[\boldsymbol{G}] \quad \text{in } \ell^{\infty}(\mathscr{H}_{\vartheta}).$$

Thus, we prove Step 5.

Finally, we proceed to the last step. For $\omega_1 \in \mathbb{R}$ and $\omega_0 \in \mathbb{R}$, choose appropriate ϑ such that $\boldsymbol{h}_{\boldsymbol{\omega}} = (\omega_1\gamma_1^{p/(4m_1)}K_{1\boldsymbol{x}_0}, \omega_0\gamma_0^{p/(4m_0)}K_{0\boldsymbol{x}_0}) \in \mathscr{H}_{\vartheta}$ and $\dot{\boldsymbol{U}}_{\gamma}^{-1}(\boldsymbol{\Upsilon}_0)[\boldsymbol{h}_{\boldsymbol{\omega}}] \in \mathscr{H}_{\vartheta}$. Of note, for $\boldsymbol{h} \in \mathcal{H}$, we have $\dot{\boldsymbol{U}}_{\gamma}(\boldsymbol{\Upsilon}_0)[\boldsymbol{h}] \geqslant c_1\|\boldsymbol{h}\|_{\mathcal{H}}$ as shown in Step 3, thus $\dot{\boldsymbol{U}}_{\gamma}^{-1}(\boldsymbol{\Upsilon}_0)[\boldsymbol{h}] \leqslant c_2\|\boldsymbol{h}\|_{\mathcal{H}}$, and further such an ϑ satisfies $\dot{\boldsymbol{U}}_{\gamma}^{-1}(\boldsymbol{\Upsilon}_0)[\boldsymbol{h}_{\boldsymbol{\omega}}] \in \mathscr{H}_{\vartheta}$ always exists. Let $(b_{\tau}, b_{\lambda}) = \dot{\boldsymbol{U}}_{\gamma}^{-1}(\boldsymbol{\Upsilon}_0)[\boldsymbol{W}_{\gamma}(\boldsymbol{\Upsilon}_0)] \in \mathcal{H}$, $\tau_0^*(\boldsymbol{x}_0) = \tau_0(\boldsymbol{x}_0) - b_{\tau}(\boldsymbol{x}_0)$, and $\lambda_0^*(\boldsymbol{x}_0) = \lambda_0(\boldsymbol{x}_0) - b_{\lambda}(\boldsymbol{x}_0)$. Then, following equation (S33), we have

$$\begin{aligned} & \omega_1\left[n^{1/2}\gamma_1^{p/(4m_1)}\{\widehat{\tau}_n(\boldsymbol{x}_0) - \tau_0^*(\boldsymbol{x}_0)\}\right] + \omega_0\left[n^{1/2}\gamma_0^{p/(4m_0)}\{\widehat{\lambda}_n(\boldsymbol{x}_0) - \lambda_0^*(\boldsymbol{x}_0)\}\right] \\ = \ & \sqrt{n}\left(\widehat{\boldsymbol{\Upsilon}}_n - \boldsymbol{\Upsilon}_0 + \dot{\boldsymbol{U}}_{\gamma}^{-1}(\boldsymbol{\Upsilon}_0)[\boldsymbol{W}_{\gamma}(\boldsymbol{\Upsilon}_0)]\right)[\boldsymbol{h}_{\boldsymbol{\omega}}]. \\ \rightsquigarrow \ & \dot{\boldsymbol{U}}_{\gamma}^{-1}(\boldsymbol{\Upsilon}_0)[\boldsymbol{G}][\boldsymbol{h}_{\omega}] \\ = \ & \boldsymbol{G}[\{\dot{\boldsymbol{U}}_{\gamma}^{-1}(\boldsymbol{\Upsilon}_0)\}^*[\boldsymbol{h}_{\omega}]] \\ = \ & \boldsymbol{G}[\dot{\boldsymbol{U}}_{\gamma}^{-1}(\boldsymbol{\Upsilon}_0)[\boldsymbol{h}_{\omega}]] \end{aligned}$$

by noting that $\dot{\boldsymbol{U}}_{\gamma}(\boldsymbol{\Upsilon}_0)$ is the self-adjoint operate, where $\{\dot{\boldsymbol{U}}_{\gamma}^{-1}(\boldsymbol{\Upsilon}_0)\}^*$ denote the adjoint operate of $\dot{\boldsymbol{U}}_{\gamma}^{-1}(\boldsymbol{\Upsilon}_0)$. That is, for every $\omega_1 \in \mathbb{R}$ and $\omega_0 \in \mathbb{R}$, $\omega_1[n^{1/2}\gamma_1^{p/(4m_1)}\{\widehat{\tau}_n(\boldsymbol{x}_0) - \tau_0^*(\boldsymbol{x}_0)\}] + \omega_0[n^{1/2}\gamma_0^{p/(4m_0)}\{\widehat{\lambda}_n(\boldsymbol{x}_0) - \lambda_0^*(\boldsymbol{x}_0)\}]$ converges to a zero-mean Gaussian distribution with the

variance

$$
\begin{aligned}
& \operatorname{Cov}\left(\boldsymbol{G}[\dot{\boldsymbol{U}}_{\boldsymbol{\gamma}}^{-1}(\boldsymbol{\Upsilon}_0)[\boldsymbol{h}_\omega]], \boldsymbol{G}[\dot{\boldsymbol{U}}_{\boldsymbol{\gamma}}^{-1}(\boldsymbol{\Upsilon}_0)[\boldsymbol{h}_\omega]]\right) \\
= \; & \varrho \mathbb{P}_1\left(\sigma_{01}^2\right)^{-1}(K_1^*)^2 + (1-\varrho)\mathbb{P}_0\left(\sigma_{00}^2\right)^{-1}(K_1^* + K_0^*)^2 \\
= \; & \varrho \mathbb{P}_1\left(\sigma_{01}^2\right)^{-1}(K_1^*)^2 + (1-\varrho)\mathbb{P}_0\left(\sigma_{00}^2\right)^{-1}(K_1^*)^2 + 2(1-\varrho)\mathbb{P}_0\left(\sigma_{00}^2\right)^{-1} K_1^* K_0^* \\
& + (1-\varrho)\mathbb{P}_0\left(\sigma_{00}^2\right)^{-1}(K_0^*)^2 \\
= \; & V_1(K_1^*, K_1^*) + V_0(K_0^*, K_0^*) + 2(1-\varrho)\mathbb{P}_0\left(\sigma_{00}^2\right)^{-1} K_1^* K_0^*,
\end{aligned}
$$

where $(K_1^*, K_0^*) = \dot{\boldsymbol{U}}_{\boldsymbol{\gamma}}^{-1}(\boldsymbol{\Upsilon}_0)[(\omega_1\gamma_1^{p/(4m_1)}K_{1\boldsymbol{x}_0}, \omega_0\gamma_0^{p/(4m_0)}K_{0\boldsymbol{x}_0})]$. Therefore,

$$
\left(n^{1/2}\gamma_1^{p/(4m_1)}\{\widehat{\tau}_n(\boldsymbol{x}_0) - \tau_0^*(\boldsymbol{x}_0)\}, n^{1/2}\gamma_0^{p/(4m_0)}\{\widehat{\lambda}_n(\boldsymbol{x}_0) - \lambda_0^*(\boldsymbol{x}_0)\}\right)^{\mathrm{T}}
$$

converges to a zero-mean bivariate Gaussian distribution. Thus, we complete the proof of Theorem 3.

E.4 Proof of Corollary 1

Proof. Here, we follow the notations that used in the proof of Theorem 3. Obviously, it is enough to show that

$$
\boldsymbol{W}_{\boldsymbol{\gamma}}(\boldsymbol{\Upsilon}_0)[\boldsymbol{h}_\vartheta] = o_P(n^{-1/2}). \tag{S54}
$$

By Lemma S7 and $n(\gamma_1+\gamma_0) = O(1)$ presented in Corollary 4.1,

$$
\begin{aligned}
n^{1/2}\left|\boldsymbol{W}_{\boldsymbol{\gamma}}(\boldsymbol{\Upsilon}_0)[\boldsymbol{h}_\vartheta]\right| &= n^{1/2}\left|\langle W_{\gamma_1}\tau_0, h_\vartheta\rangle_{\mathcal{H}_1} + \langle W_{\gamma_0}\lambda_0, g_\vartheta\rangle_{\mathcal{H}_0}\right| \\
&\leqslant n^{1/2}\left(\|W_{\gamma_1}\tau_0\|_{\mathcal{H}_1}\|f_\vartheta\|_{\mathcal{H}_1} + \|W_{\gamma_0}\lambda_0\|_{\mathcal{H}_0}\|g_\vartheta\|_{\mathcal{H}_0}\right) \\
&\leqslant n^{1/2}\left(o(\gamma_1^{1/2})O(1) + o(\gamma_0^{1/2})O(1)\right) \\
&= o\left(\{n(\gamma_1+\gamma_0)\}^{1/2}\right) \\
&= o(1),
\end{aligned}
$$

which implies equation (S54). Thus, we complete the proof of Corollary 1.

E.5 Proof of Theorem 4

Proof. For two symmetric matrixes $\boldsymbol{B}_1$ and $\boldsymbol{B}_2$, we denote $\boldsymbol{B}_1 \geqslant \boldsymbol{B}_2$ or $\boldsymbol{B}_2 \leqslant \boldsymbol{B}_1$ if $\boldsymbol{B}_1 - \boldsymbol{B}_2$ is positive semi-definite. Let $\rho(\boldsymbol{B}_1)$, $\rho_{\min}(\boldsymbol{B}_1)$, and $\rho_{\max}(\boldsymbol{B}_1)$ be the eigenvalues, minimum, and maximum eigenvalues of $\boldsymbol{B}_1$, respectively. Let $\boldsymbol{H}$ be an $n \times n$ diagonal matrix with the main diagonal elements being $(\{\widehat{\sigma}^2(\boldsymbol{X}_1, S_1)\}^{-1}, \ldots, \{\widehat{\sigma}^2(\boldsymbol{X}_n, S_n)\}^{-1})^{\mathrm{T}}$, $\boldsymbol{H}_1$ be the first $n_1 \times n_1$ matrix of $\boldsymbol{H}$, and $\boldsymbol{H}_2$ be the last $n_0 \times n_0$ matrix of $\boldsymbol{H}$. Let $\boldsymbol{X}^* = (\boldsymbol{X}_1, \ldots, \boldsymbol{X}_n)^{\mathrm{T}}$, $\boldsymbol{S} = (S_1, \ldots, S_n)^{\mathrm{T}}$, $\boldsymbol{\phi}(\boldsymbol{x}) = (\phi_1(\boldsymbol{x}), \ldots, \phi_{r_1}(\boldsymbol{x}))^{\mathrm{T}}$, $\boldsymbol{\psi}(\boldsymbol{x}) = (\psi_1(\boldsymbol{x}), \ldots, \psi_{r_0}(\boldsymbol{x}))^{\mathrm{T}}$, $\boldsymbol{\Phi}(\boldsymbol{X}^*) = (\boldsymbol{\phi}(\boldsymbol{X}_1), \ldots, \boldsymbol{\phi}(\boldsymbol{X}_n))^{\mathrm{T}}$, $\boldsymbol{\Psi}(\boldsymbol{X}^*) = (\boldsymbol{\psi}(\boldsymbol{X}_1), \ldots, \boldsymbol{\psi}(\boldsymbol{X}_n))^{\mathrm{T}}$, and $\boldsymbol{A} = (\boldsymbol{\Phi}(\boldsymbol{X}^*), (1 - \boldsymbol{S})\boldsymbol{\Psi}(\boldsymbol{X}^*))$. Decompose $\boldsymbol{A}$ into an 2×2 block matrix form with the $(1,1)$-th elements being $\boldsymbol{A}_1$, $(1,2)$-th element being $\boldsymbol{0}$, $(2,1)$-th element being $\boldsymbol{A}_{21}$, and $(2,2)$-th element being $\boldsymbol{A}_2$, where $\boldsymbol{A}_1$ is an $n_1 \times r_1$ matrix, $\boldsymbol{A}_{21}$is an $n_0 \times r_1$ matrix, and $\boldsymbol{A}_2$ is an $n_0 \times r_0$ matrix. Let $\boldsymbol{P}_1$ be an $r_1 \times r_1$ matrix with (i,j)-th element being $J_1(\phi_i, \phi_j)$, where $1 \leqslant i, j \leqslant r_1$, and $\boldsymbol{P}_2$ be an $r_0 \times r_0$ matrix with (i', j')-th element being $J_0(\psi_{i'}, \psi_{j'})$, where $1 \leqslant i', j' \leqslant r_0$, $\boldsymbol{P}$ be an 2×2 diagonal block matrix with $(1,1)$-th element being $\boldsymbol{P}_1$ and $(2,2)$-th element being $\boldsymbol{P}_2$, and $\boldsymbol{P}_{\boldsymbol{\gamma}}$ be an 2×2 diagonal block matrix with $(1,1)$-th element being $\gamma_1 \boldsymbol{P}_1$ and $(2,2)$-th element being $\gamma_0 \boldsymbol{P}_2$. Let $\widehat{\boldsymbol{D}}_1 = (\widehat{D}_1, \ldots, \widehat{D}_{n_1})^{\mathrm{T}}$, $\boldsymbol{D}_2 = (D_{n_1+1}, \ldots, D_n)^{\mathrm{T}}$, $\widehat{\boldsymbol{D}} = (\widehat{\boldsymbol{D}}_1^{\mathrm{T}}, \boldsymbol{D}_2^{\mathrm{T}})^{\mathrm{T}}$.

Note that $\tau(\boldsymbol{X})$ and $\lambda(\boldsymbol{X})$ can be approximated by the corresponding parameterized functions $\boldsymbol{\phi}(\boldsymbol{X})^{\mathrm{T}}\boldsymbol{\alpha}$ and $\boldsymbol{\psi}(\boldsymbol{X})^{\mathrm{T}}\boldsymbol{\beta}$, respectively, where $\boldsymbol{\alpha} = (\alpha_1, \ldots, \alpha_{r_1})^{\mathrm{T}}$ and $\boldsymbol{\beta} = (\beta_1, \ldots, \beta_{r_0})^{\mathrm{T}}$. Then minimizing $\widehat{\ell}_{n,\gamma_1,\gamma_0}(\tau, \lambda)$ over $\Phi_n \times \Psi_n$ is equivalent to minimizing

$$\begin{aligned} & \widehat{l}_{n,\gamma_1,\gamma_0}(\boldsymbol{\alpha}, \boldsymbol{\beta}) \\ = \ & \left\{\widehat{\boldsymbol{D}} - \boldsymbol{\Phi}(\boldsymbol{X}^*)\boldsymbol{\alpha} - (1 - \boldsymbol{S})\boldsymbol{\Psi}(\boldsymbol{X}^*)\boldsymbol{\beta}\right\}^{\mathrm{T}} \boldsymbol{H} \left\{\widehat{\boldsymbol{D}} - \boldsymbol{\Phi}(\boldsymbol{X}^*)\boldsymbol{\alpha} - (1 - \boldsymbol{S})\boldsymbol{\Psi}(\boldsymbol{X}^*)\boldsymbol{\beta}\right\} \\ & + n\gamma_1 \boldsymbol{\alpha}^{\mathrm{T}} \boldsymbol{P}_1 \boldsymbol{\alpha} + n\gamma_0 \boldsymbol{\beta}^{\mathrm{T}} \boldsymbol{P}_2 \boldsymbol{\beta}. \end{aligned}$$

Let $\boldsymbol{\theta} = (\boldsymbol{\alpha}^{\mathrm{T}}, \boldsymbol{\beta}^{\mathrm{T}})^{\mathrm{T}}$, then $\widehat{l}_{n,\gamma_1,\gamma_0}(\boldsymbol{\alpha}, \boldsymbol{\beta})$ can be rewritten as

$$\widehat{l}_{n,\gamma_1,\gamma_0}(\boldsymbol{\theta}) = \left(\widehat{\boldsymbol{D}} - \boldsymbol{A}\boldsymbol{\theta}\right)^{\mathrm{T}} \boldsymbol{H} \left(\widehat{\boldsymbol{D}} - \boldsymbol{A}\boldsymbol{\theta}\right) + n\boldsymbol{\theta}^{\mathrm{T}} \boldsymbol{P}_{\boldsymbol{\gamma}} \boldsymbol{\theta},$$

and the corresponding minimizer can be deduced as

$$\widehat{\boldsymbol{\theta}}_n = \left(\widehat{\boldsymbol{\alpha}}_n^{\mathrm{T}}, \widehat{\boldsymbol{\beta}}_n^{\mathrm{T}}\right)^{\mathrm{T}} = (\boldsymbol{A}^{\mathrm{T}}\boldsymbol{H}\boldsymbol{A} + n\boldsymbol{P}_{\gamma})^{-1}\boldsymbol{A}^{\mathrm{T}}\boldsymbol{H}\widehat{\boldsymbol{D}}.$$

Therefore, for a given point $\boldsymbol{x}_0 \in \Omega$, the estimator of $\tau_0(\boldsymbol{x}_0)$ can be expressed by

$$\widehat{\tau}_n(\boldsymbol{x}_0) = \boldsymbol{\phi}(\boldsymbol{x}_0)^{\mathrm{T}}\widehat{\boldsymbol{\alpha}}_n.$$

Using an similar argument, $\widehat{\tau}_{\mathrm{rct}}(\boldsymbol{x}_0)$ can be expressed by

$$\widehat{\tau}_{\mathrm{rct}}(\boldsymbol{x}_0) = \boldsymbol{\phi}(\boldsymbol{x}_0)^{\mathrm{T}}\widehat{\boldsymbol{\alpha}}_{\mathrm{rct}},$$

where $\widehat{\boldsymbol{\alpha}}_{\mathrm{rct}} = (\boldsymbol{A}_1^{\mathrm{T}}\boldsymbol{H}_1\boldsymbol{A}_1 + n\gamma_1\boldsymbol{P}_1)^{-1}\boldsymbol{A}_1^{\mathrm{T}}\boldsymbol{H}_1\widehat{\boldsymbol{D}}_1$. It follows that

$$\mathrm{Var}_r(\widehat{\tau}_{\mathrm{rct}}(\boldsymbol{x}_0)) = \boldsymbol{\phi}(\boldsymbol{x}_0)^{\mathrm{T}}\mathrm{Var}_r(\widehat{\boldsymbol{\alpha}}_{\mathrm{rct}})\boldsymbol{\phi}(\boldsymbol{x}_0),\ \mathrm{Var}_r(\widehat{\tau}_n(\boldsymbol{x}_0)) = \boldsymbol{\phi}(\boldsymbol{x}_0)^{\mathrm{T}}\mathrm{Var}_r(\widehat{\boldsymbol{\alpha}}_n)\boldsymbol{\phi}(\boldsymbol{x}_0).$$

We first consider $\mathrm{Var}_r(\widehat{\tau}_{\mathrm{rct}}(\boldsymbol{x}_0))$.

$$\begin{aligned}\mathrm{Var}_r(\widehat{\boldsymbol{\alpha}}_{\mathrm{rct}}) &= E_r\left\{(\boldsymbol{A}_1^{\mathrm{T}}\boldsymbol{H}_1\boldsymbol{A}_1 + n\gamma_1\boldsymbol{P}_1)^{-1}\boldsymbol{A}_1^{\mathrm{T}}\boldsymbol{H}_1\mathrm{Var}_r(\widehat{\boldsymbol{D}}_1 \mid \boldsymbol{X}^*)\boldsymbol{H}_1\boldsymbol{A}_1(\boldsymbol{A}_1^{\mathrm{T}}\boldsymbol{H}_1\boldsymbol{A}_1 + n\gamma_1\boldsymbol{P}_1)^{-1}\right\}\\ &\quad +\mathrm{Var}_r\left\{(\boldsymbol{A}_1^{\mathrm{T}}\boldsymbol{H}_1\boldsymbol{A}_1 + n\gamma_1\boldsymbol{P}_1)^{-1}\boldsymbol{A}_1^{\mathrm{T}}\boldsymbol{H}_1E_r(\widehat{\boldsymbol{D}}_1 \mid \boldsymbol{X}^*)\right\}\\ &= \Xi_1 + \Xi_2,\end{aligned}$$

where Ξ and Ξ_2 are clear from the above equation, furthermore,

$$\mathrm{Var}_r\{\widehat{\tau}_{\mathrm{rct}}(\boldsymbol{x}_0)\} = \boldsymbol{\phi}(\boldsymbol{x}_0)^{\mathrm{T}}\Xi_1\boldsymbol{\phi}(\boldsymbol{x}_0) + \boldsymbol{\phi}(\boldsymbol{x}_0)^{\mathrm{T}}\Xi_2\boldsymbol{\phi}(\boldsymbol{x}_0).$$

Next, we show that $\boldsymbol{\phi}(\boldsymbol{x}_0)^{\mathrm{T}}\Xi_2\boldsymbol{\phi}(\boldsymbol{x}_0)$ is dominated by $\boldsymbol{\phi}(\boldsymbol{x}_0)^{\mathrm{T}}\Xi_1\boldsymbol{\phi}(\boldsymbol{x}_0)$. Let $\boldsymbol{\zeta}^{\dagger}(\boldsymbol{X}^*) = (\boldsymbol{A}_1^{\mathrm{T}}\boldsymbol{H}_1\boldsymbol{A}_1 + n\gamma_1\boldsymbol{P}_1)^{-1}\boldsymbol{A}_1^{\mathrm{T}}\boldsymbol{H}_1\boldsymbol{A}_1(\boldsymbol{A}_1^{\mathrm{T}}\boldsymbol{H}_1\boldsymbol{A}_1 + n\gamma_1\boldsymbol{P}_1)^{-1}$. By Condition S15,

$$\boldsymbol{\phi}(\boldsymbol{x}_0)^{\mathrm{T}}\Xi_1\boldsymbol{\phi}(\boldsymbol{x}_0) = E_r\left\{\boldsymbol{\phi}(\boldsymbol{x}_0)^{\mathrm{T}}\boldsymbol{\zeta}^{\dagger}(\boldsymbol{X}^*)\boldsymbol{\phi}(\boldsymbol{x}_0)\right\} + o_P(1)E_r\left\{\boldsymbol{\phi}(\boldsymbol{x}_0)^{\mathrm{T}}\boldsymbol{\zeta}^{\dagger}(\boldsymbol{X}^*)\boldsymbol{\phi}(\boldsymbol{x}_0)\right\}. \quad \text{(S55)}$$

Under Conditions S3 and S16, we have

$$\begin{aligned}\rho_{\min}(\boldsymbol{A}_1^{\mathrm{T}}\boldsymbol{H}_1\boldsymbol{A}_1) \geqslant \rho_{\min}(\boldsymbol{H}_1)\rho_{\min}(\boldsymbol{A}_1^{\mathrm{T}}\boldsymbol{A}_1) &\geqslant c_1\rho_{\min}(\boldsymbol{A}^{\mathrm{T}}\boldsymbol{A})\\ &\geqslant c_2 n^{1-\kappa^*},\\ \rho_{\max}(\boldsymbol{A}_1^{\mathrm{T}}\boldsymbol{H}_1\boldsymbol{A}_1) \leqslant \rho_{\max}(\boldsymbol{H}_1)\rho_{\max}(\boldsymbol{A}_1^{\mathrm{T}}\boldsymbol{A}_1) &\leqslant c_3\rho_{\max}(\boldsymbol{A}^{\mathrm{T}}\boldsymbol{A})\\ &\leqslant c_4 n^{1-\kappa^*},\end{aligned}$$

that is

$$\rho(\boldsymbol{A}_1^{\mathrm{T}}\boldsymbol{H}_1\boldsymbol{A}_1) \asymp n^{1-\kappa^*}. \tag{S56}$$

Under Conditions S3, S16, and S17, it follow from equation (S56) that

$$\begin{aligned}
\rho_{\min}\left\{(\boldsymbol{A}_1^{\mathrm{T}}\boldsymbol{H}_1\boldsymbol{A}_1 + n\gamma_1\boldsymbol{P}_1)^{-1}\right\} &= \left\{\rho_{\max}(\boldsymbol{A}_1^{\mathrm{T}}\boldsymbol{H}_1\boldsymbol{A}_1 + n\gamma_1\boldsymbol{P}_1)\right\}^{-1} \\
&\geqslant \left\{\rho_{\max}(\boldsymbol{A}_1^{\mathrm{T}}\boldsymbol{H}_1\boldsymbol{A}_1) + n\gamma_1\rho_{\max}(\boldsymbol{P}_1)\right\}^{-1} \\
&\geqslant \left\{c_5 n^{1-\kappa^*} + c_6\gamma_1 n^{1+\kappa_1^{\dagger}}\right\}^{-1} \\
&\geqslant c_7 n^{\kappa^*-1} \\
\rho_{\max}\left\{(\boldsymbol{A}_1^{\mathrm{T}}\boldsymbol{H}_1\boldsymbol{A}_1 + n\gamma_1\boldsymbol{P}_1)^{-1}\right\} &= \left\{\rho_{\min}(\boldsymbol{A}_1^{\mathrm{T}}\boldsymbol{H}_1\boldsymbol{A}_1 + n\gamma_1\boldsymbol{P}_1)\right\}^{-1} \\
&\leqslant \left\{c_8 n^{1-\kappa^*} + n\gamma_1\rho_{\min}(\boldsymbol{P}_1)\right\}^{-1} \\
&\leqslant c_9 n^{\kappa^*-1},
\end{aligned}$$

that is,

$$\rho\left\{(\boldsymbol{A}_1^{\mathrm{T}}\boldsymbol{H}_1\boldsymbol{A}_1 + n\gamma_1\boldsymbol{P}_1)^{-1}\right\} \asymp n^{\kappa^*-1}. \tag{S57}$$

Thus

$$\begin{aligned}
\boldsymbol{\phi}(\boldsymbol{x}_0)^{\mathrm{T}}\boldsymbol{\zeta}^{\dagger}(\boldsymbol{X}^*)\boldsymbol{\phi}(\boldsymbol{x}_0) &\leqslant \boldsymbol{\phi}(\boldsymbol{x}_0)^{\mathrm{T}}(\boldsymbol{A}_1^{\mathrm{T}}\boldsymbol{H}_1\boldsymbol{A}_1)^{-1}\boldsymbol{\phi}(\boldsymbol{x}_0) \\
&\leqslant \rho_{\max}\left\{(\boldsymbol{A}_1^{\mathrm{T}}\boldsymbol{H}_1\boldsymbol{A}_1)^{-1}\right\}\boldsymbol{\phi}(\boldsymbol{x}_0)^{\mathrm{T}}\boldsymbol{\phi}(\boldsymbol{x}_0) \\
&= \left\{\rho_{\min}(\boldsymbol{A}_1^{\mathrm{T}}\boldsymbol{H}_1\boldsymbol{A}_1)\right\}^{-1}\boldsymbol{\phi}(\boldsymbol{x}_0)^{\mathrm{T}}\boldsymbol{\phi}(\boldsymbol{x}_0) \\
&\leqslant c_{10} n^{\kappa^*-1}\boldsymbol{\phi}(\boldsymbol{x}_0)^{\mathrm{T}}\boldsymbol{\phi}(\boldsymbol{x}_0), \\
\boldsymbol{\phi}(\boldsymbol{x}_0)^{\mathrm{T}}\boldsymbol{\zeta}^{\dagger}(\boldsymbol{X}^*)\boldsymbol{\phi}(\boldsymbol{x}_0) &\geqslant \boldsymbol{\phi}(\boldsymbol{x}_0)^{\mathrm{T}}(\boldsymbol{A}_1^{\mathrm{T}}\boldsymbol{H}_1\boldsymbol{A}_1 + n\gamma_1\boldsymbol{P}_1)^{-1}\boldsymbol{\phi}(\boldsymbol{x}_0) \\
&\geqslant \rho_{\min}\left\{(\boldsymbol{A}_1^{\mathrm{T}}\boldsymbol{H}_1\boldsymbol{A}_1 + n\gamma_1\boldsymbol{P}_1)^{-1}\right\}\boldsymbol{\phi}(\boldsymbol{x}_0)^{\mathrm{T}}\boldsymbol{\phi}(\boldsymbol{x}_0) \\
&\geqslant c_{11} n^{\kappa^*-1}\boldsymbol{\phi}(\boldsymbol{x}_0)^{\mathrm{T}}\boldsymbol{\phi}(\boldsymbol{x}_0).
\end{aligned}$$

As a consequence,

$$\boldsymbol{\phi}(\boldsymbol{x}_0)^{\mathrm{T}}\boldsymbol{\Xi}_1\boldsymbol{\phi}(\boldsymbol{x}_0) \asymp n^{\kappa^*-1}\boldsymbol{\phi}(\boldsymbol{x}_0)^{\mathrm{T}}\boldsymbol{\phi}(\boldsymbol{x}_0). \tag{S58}$$

We next consider $\boldsymbol{\phi}(\boldsymbol{x}_0)^{\mathrm{T}}\boldsymbol{\Xi}_2\boldsymbol{\phi}(\boldsymbol{x}_0)$. Let $\Gamma(\boldsymbol{X}_i) = \Gamma_1(\boldsymbol{X}_i) + \Gamma_2(\boldsymbol{X}_i)$, $i = 1, \ldots, n_1$, $\boldsymbol{\Gamma}(\boldsymbol{X}_1^*) = (\Gamma(\boldsymbol{X}_1), \ldots, \Gamma(\boldsymbol{X}_{n_1}))^{\mathrm{T}}$, and $\boldsymbol{\tau}_0(\boldsymbol{X}_1^*) = (\tau_0(\boldsymbol{X}_1), \ldots, \tau_0(\boldsymbol{X}_{n_1}))$, then by Lemma S2,

$$
\begin{aligned}
& \boldsymbol{\phi}(\boldsymbol{x}_0)^{\mathrm{T}}\boldsymbol{\Xi}_2\boldsymbol{\phi}(\boldsymbol{x}_0) \\
= \; & \boldsymbol{\phi}(\boldsymbol{x}_0)^{\mathrm{T}}\mathrm{Var}_r \left[(\boldsymbol{A}_1^{\mathrm{T}}\boldsymbol{H}_1\boldsymbol{A}_1 + n\gamma_1\boldsymbol{P}_1)^{-1}\boldsymbol{A}_1^{\mathrm{T}}\boldsymbol{H}_1 \left\{E_r(\widehat{\boldsymbol{D}}_1 - \boldsymbol{D}_1 \mid \boldsymbol{X}^*) + E_r(\boldsymbol{D}_1 \mid \boldsymbol{X}^*)\right\}\right] \boldsymbol{\phi}(\boldsymbol{x}_0) \\
= \; & \boldsymbol{\phi}(\boldsymbol{x}_0)^{\mathrm{T}}\mathrm{Var}_r \left[(\boldsymbol{A}_1^{\mathrm{T}}\boldsymbol{H}_1\boldsymbol{A}_1 + n\gamma_1\boldsymbol{P}_1)^{-1}\boldsymbol{A}_1^{\mathrm{T}}\boldsymbol{H}_1 \left\{\boldsymbol{\Gamma}(\boldsymbol{X}_1^*) + \boldsymbol{\tau}_0(\boldsymbol{X}_1^*)\right\}\right] \boldsymbol{\phi}(\boldsymbol{x}_0) \\
\leqslant \; & 2\boldsymbol{\phi}(\boldsymbol{x}_0)^{\mathrm{T}}\mathrm{Var}_r \left[(\boldsymbol{A}_1^{\mathrm{T}}\boldsymbol{H}_1\boldsymbol{A}_1 + n\gamma_1\boldsymbol{P}_1)^{-1}\boldsymbol{A}_1^{\mathrm{T}}\boldsymbol{H}_1\boldsymbol{\Gamma}(\boldsymbol{X}_1^*)\right] \boldsymbol{\phi}(\boldsymbol{x}_0) \\
& +2\boldsymbol{\phi}(\boldsymbol{x}_0)^{\mathrm{T}}\mathrm{Var}_r \left\{(\boldsymbol{A}_1^{\mathrm{T}}\boldsymbol{H}_1\boldsymbol{A}_1 + n\gamma_1\boldsymbol{P}_1)^{-1}\boldsymbol{A}_1^{\mathrm{T}}\boldsymbol{H}_1\boldsymbol{\tau}_0(\boldsymbol{X}_1^*)\right\} \boldsymbol{\phi}(\boldsymbol{x}_0) \\
= \; & 2I_{n1}^{\ddagger} + 2I_{n2}^{\ddagger}, \qquad \text{(S59)}
\end{aligned}
$$

where $I_{n1}^{\ddagger}$ and $I_{n2}^{\ddagger}$ are clear from the above equation. Let $\boldsymbol{w}_j$ denote the j-th column of $(\boldsymbol{A}_1^{\mathrm{T}}\boldsymbol{H}_1\boldsymbol{A}_1 + n\gamma_1\boldsymbol{P}_1)^{-1}\boldsymbol{A}_1^{\mathrm{T}}\boldsymbol{H}_1$, then $(\boldsymbol{w}_1, \ldots, \boldsymbol{w}_{n_1}) = (\boldsymbol{A}_1^{\mathrm{T}}\boldsymbol{H}_1\boldsymbol{A}_1 + n\gamma_1\boldsymbol{P}_1)^{-1}\boldsymbol{A}_1^{\mathrm{T}}\boldsymbol{H}_1$, then by

equation (S57), Conditions S3, S10, and S16,

$$\begin{aligned}
I_{n1}^{\ddagger} &= \mathrm{Var}_r\left\{\boldsymbol{\phi}(\boldsymbol{x}_0)^{\mathrm{T}}\sum_{j=1}^{n_1}\boldsymbol{w}_j\Gamma(\boldsymbol{X}_j)\right\} \\
&= \mathrm{Var}_r\left\{\sum_{j=1}^{n_1}\Gamma(\boldsymbol{X}_j)\boldsymbol{\phi}(\boldsymbol{x}_0)^{\mathrm{T}}\boldsymbol{w}_j\right\} \\
&\leqslant E_r\left\{\sum_{j=1}^{n_1}\Gamma(\boldsymbol{X}_j)\boldsymbol{\phi}(\boldsymbol{x}_0)^{\mathrm{T}}\boldsymbol{w}_j\right\}^2 \\
&\leqslant E_r\left[\sum_{j=1}^{n_1}(\Gamma(\boldsymbol{X}_j))^2\sum_{j=1}^{n_1}\{\boldsymbol{\phi}(\boldsymbol{x}_0)^{\mathrm{T}}\boldsymbol{w}_j\}^2\right] \\
&= E_r\left[\sum_{j=1}^{n_1}(\Gamma(\boldsymbol{X}_j))^2\sum_{j=1}^{n_1}\{\boldsymbol{\phi}(\boldsymbol{x}_0)^{\mathrm{T}}\boldsymbol{w}_j\boldsymbol{w}_j^{\mathrm{T}}\boldsymbol{\phi}(\boldsymbol{x}_0)\right] \\
&= E_r\Bigg[\left\{\sum_{j=1}^{n_1}(\Gamma(\boldsymbol{X}_j))^2\right\}\boldsymbol{\phi}(\boldsymbol{x}_0)^{\mathrm{T}}(\boldsymbol{A}_1^{\mathrm{T}}\boldsymbol{H}_1\boldsymbol{A}_1+n\gamma_1\boldsymbol{P}_1)^{-1}\boldsymbol{A}_1^{\mathrm{T}}\boldsymbol{H}_1^2\boldsymbol{A}_1 \\
&\quad\times(\boldsymbol{A}_1^{\mathrm{T}}\boldsymbol{H}_1\boldsymbol{A}_1+n\gamma_1\boldsymbol{P}_1)^{-1}\boldsymbol{\phi}(\boldsymbol{x}_0)\Bigg] \\
&\leqslant \left[\rho_{\max}\left\{(\boldsymbol{A}_1^{\mathrm{T}}\boldsymbol{H}_1\boldsymbol{A}_1+n\gamma_1\boldsymbol{P}_1)^{-1}\right\}\right]^2\rho_{\max}(\boldsymbol{A}_1^{\mathrm{T}}\boldsymbol{A}_1)\rho_{\max}(\boldsymbol{H}_1^2)\boldsymbol{\phi}(\boldsymbol{x}_0)^{\mathrm{T}}\boldsymbol{\phi}(\boldsymbol{x}_0) \\
&\quad\times n_1E_r\{\Gamma_1(\boldsymbol{X})+\Gamma_2(\boldsymbol{X})\}^2 \\
&\leqslant O(1)n^{2(\kappa^*-1)}n^{1-\kappa^*}\boldsymbol{\phi}(\boldsymbol{x}_0)^{\mathrm{T}}\boldsymbol{\phi}(\boldsymbol{x}_0)n_1o_P(n^{-1}) \\
&= o_P\left(n^{\kappa^*-1}\right)\boldsymbol{\phi}(\boldsymbol{x}_0)^{\mathrm{T}}\boldsymbol{\phi}(\boldsymbol{x}_0). \qquad \text{(S60)}
\end{aligned}$$

For $I_{n2}^{\ddagger}$,

$$\begin{aligned}
I_{n2}^{\ddagger} &= \boldsymbol{\phi}(\boldsymbol{x}_0)^{\mathrm{T}}\mathrm{Var}_r\left[(\boldsymbol{A}_1^{\mathrm{T}}\boldsymbol{H}_1\boldsymbol{A}_1+n\gamma_1\boldsymbol{P}_1)^{-1}\boldsymbol{A}_1^{\mathrm{T}}\boldsymbol{H}_1\{\boldsymbol{\tau}_0(\boldsymbol{X}_1^*)-\boldsymbol{\tau}_n(\boldsymbol{X}_1^*)+\boldsymbol{\tau}_n(\boldsymbol{X}_1^*)\}\right]\boldsymbol{\phi}(\boldsymbol{x}_0) \\
&\leqslant 2\boldsymbol{\phi}(\boldsymbol{x}_0)^{\mathrm{T}}\mathrm{Var}_r\left[(\boldsymbol{A}_1^{\mathrm{T}}\boldsymbol{H}_1\boldsymbol{A}_1+n\gamma_1\boldsymbol{P}_1)^{-1}\boldsymbol{A}_1^{\mathrm{T}}\boldsymbol{H}_1\{\boldsymbol{\tau}_0(\boldsymbol{X}_1^*)-\boldsymbol{\tau}_n(\boldsymbol{X}_1^*)\}\right]\boldsymbol{\phi}(\boldsymbol{x}_0) \\
&\quad+2\boldsymbol{\phi}(\boldsymbol{x}_0)^{\mathrm{T}}\mathrm{Var}_r\left\{(\boldsymbol{A}_1^{\mathrm{T}}\boldsymbol{H}_1\boldsymbol{A}_1+n\gamma_1\boldsymbol{P}_1)^{-1}\boldsymbol{A}_1^{\mathrm{T}}\boldsymbol{H}_1\boldsymbol{\tau}_n(\boldsymbol{X}_1^*)\right\}\boldsymbol{\phi}(\boldsymbol{x}_0) \\
&= 2I_{n2,1}^{\ddagger}+2I_{n2,2}^{\ddagger}, \qquad \text{(S61)}
\end{aligned}$$

where $I_{n2,1}^{\ddagger}$ and $I_{n2,2}^{\ddagger}$ are self-explained from the above equation and

$$\boldsymbol{\tau}_n(\boldsymbol{X}_1^*) = (\tau_n(\boldsymbol{X}_1),\ldots,\tau_n(\boldsymbol{X}_{n_1}))^{\mathrm{T}}.$$

Employing equations (S8), (S57), and Conditions S3, S16–S17, and analogous arguments as

we did in equation (S60), we conclude

$$I^{\ddagger}_{n2,1} = O_P\left(n^{1-2\kappa_1}\right) n^{\kappa^*-1}\boldsymbol{\phi}(\boldsymbol{x}_0)^{\mathrm{T}}\boldsymbol{\phi}(\boldsymbol{x}_0) = o_P\left(n^{\kappa^*-1}\right)\boldsymbol{\phi}(\boldsymbol{x}_0)^{\mathrm{T}}\boldsymbol{\phi}(\boldsymbol{x}_0). \tag{S62}$$

We now deal with $I^{\ddagger}_{n2,2}$. Noting that $\tau_n \in \Phi_n$, then $\tau_n(\boldsymbol{x})$ can be written as $\tau_n(\boldsymbol{x}) = \boldsymbol{\phi}(\boldsymbol{x})^{\mathrm{T}}\boldsymbol{\alpha}_n$ for some $\boldsymbol{\alpha}_n = (\alpha_{n1}, \ldots, \alpha_{nr_1})^{\mathrm{T}}$. Thus $\boldsymbol{\tau}_n(\boldsymbol{X}^*_1) = \boldsymbol{A}_1\boldsymbol{\alpha}_n$, and further by equation (S8) and (S57), Conditions S16–S17,

$$\begin{aligned}
I^{\ddagger}_{n2,2} &= \boldsymbol{\phi}(\boldsymbol{x}_0)^{\mathrm{T}}\mathrm{Var}_r\left\{(\boldsymbol{A}_1^{\mathrm{T}}\boldsymbol{H}_1\boldsymbol{A}_1 + n\gamma_1\boldsymbol{P}_1)^{-1}\boldsymbol{A}_1^{\mathrm{T}}\boldsymbol{H}_1\boldsymbol{A}_1\boldsymbol{\alpha}_n\right\}\boldsymbol{\phi}(\boldsymbol{x}_0) \\
&= \boldsymbol{\phi}(\boldsymbol{x}_0)^{\mathrm{T}}\mathrm{Var}_r\big[\big\{(\boldsymbol{A}_1^{\mathrm{T}}\boldsymbol{H}_1\boldsymbol{A}_1)^{-1} - (\boldsymbol{A}_1^{\mathrm{T}}\boldsymbol{H}_1\boldsymbol{A}_1)^{-1}n\gamma_1\boldsymbol{P}_1(\boldsymbol{A}_1^{\mathrm{T}}\boldsymbol{H}_1\boldsymbol{A}_1 + n\gamma_1\boldsymbol{P}_1)^{-1}\big\} \\
&\quad \times\boldsymbol{A}_1^{\mathrm{T}}\boldsymbol{H}_1\boldsymbol{A}_1\boldsymbol{\alpha}_n\big]\boldsymbol{\phi}(\boldsymbol{x}_0) \\
&= \boldsymbol{\phi}(\boldsymbol{x}_0)^{\mathrm{T}}\mathrm{Var}_r\left\{\boldsymbol{\alpha}_n - n\gamma_1(\boldsymbol{A}_1^{\mathrm{T}}\boldsymbol{H}_1\boldsymbol{A}_1)^{-1}\boldsymbol{P}_1(\boldsymbol{A}_1^{\mathrm{T}}\boldsymbol{H}_1\boldsymbol{A}_1 + n\gamma_1\boldsymbol{P}_1)^{-1}\boldsymbol{A}_1^{\mathrm{T}}\boldsymbol{H}_1\boldsymbol{A}_1\boldsymbol{\alpha}_n\right\}\boldsymbol{\phi}(\boldsymbol{x}_0) \\
&= n^2\gamma_1^2\boldsymbol{\phi}(\boldsymbol{x}_0)^{\mathrm{T}}\mathrm{Var}_r\left\{(\boldsymbol{A}_1^{\mathrm{T}}\boldsymbol{H}_1\boldsymbol{A}_1)^{-1}\boldsymbol{P}_1(\boldsymbol{A}_1^{\mathrm{T}}\boldsymbol{H}_1\boldsymbol{A}_1 + n\gamma_1\boldsymbol{P}_1)^{-1}\boldsymbol{A}_1^{\mathrm{T}}\boldsymbol{H}_1\boldsymbol{A}_1\boldsymbol{\alpha}_n\right\}\boldsymbol{\phi}(\boldsymbol{x}_0) \\
&\leqslant n^2\gamma_1^2\boldsymbol{\phi}(\boldsymbol{x}_0)^{\mathrm{T}}E_r\Big\{(\boldsymbol{A}_1^{\mathrm{T}}\boldsymbol{H}_1\boldsymbol{A}_1)^{-1}\boldsymbol{P}_1(\boldsymbol{A}_1^{\mathrm{T}}\boldsymbol{H}_1\boldsymbol{A}_1 + n\gamma_1\boldsymbol{P}_1)^{-1}\boldsymbol{A}_1^{\mathrm{T}}\boldsymbol{H}_1(\boldsymbol{A}_1\boldsymbol{\alpha}_n) \\
&\quad \times(\boldsymbol{\alpha}^{\mathrm{T}}\boldsymbol{A}_1^{\mathrm{T}})\boldsymbol{H}_1\boldsymbol{A}_1(\boldsymbol{A}_1^{\mathrm{T}}\boldsymbol{H}_1\boldsymbol{A}_1 + n\gamma_1\boldsymbol{P}_1)^{-1}\boldsymbol{P}_1(\boldsymbol{A}_1^{\mathrm{T}}\boldsymbol{H}_1\boldsymbol{A}_1)^{-1}\Big\}\boldsymbol{\phi}(\boldsymbol{x}_0) \\
&\leqslant O(1)n^2\gamma_1^2\rho_{\max}\left\{\boldsymbol{\tau}_n(\boldsymbol{X}^*)\boldsymbol{\tau}_n(\boldsymbol{X}^*)^{\mathrm{T}}\right\}\left[\rho_{\max}\left\{(\boldsymbol{A}_1^{\mathrm{T}}\boldsymbol{H}_1\boldsymbol{A}_1)^{-1}\right\}\right]^2\left\{\rho_{\max}(\boldsymbol{P}_1)\right\}^2 \\
&\quad \times\left[\rho_{\max}\left\{(\boldsymbol{A}_1^{\mathrm{T}}\boldsymbol{H}_1\boldsymbol{A}_1 + n\gamma_1\boldsymbol{P}_1)^{-1}\right\}\right]^2\rho_{\max}\left(\boldsymbol{A}_1^{\mathrm{T}}\boldsymbol{H}_1\boldsymbol{A}_1\right)\boldsymbol{\phi}(\boldsymbol{x}_0)^{\mathrm{T}}\boldsymbol{\phi}(\boldsymbol{x}_0) \\
&\leqslant O(1)n^2\gamma_1^2 n n^{2(\kappa^*-1)}n^{2\kappa^{\dagger}}n^{2(\kappa^*-1)}n^{1-\kappa^*}\boldsymbol{\phi}(\boldsymbol{x}_0)^{\mathrm{T}}\boldsymbol{\phi}(\boldsymbol{x}_0) \\
&= O\left(n^{1+2(\kappa^*+\kappa^{\dagger})}\gamma_1^2\right)n^{\kappa^*-1}\boldsymbol{\phi}(\boldsymbol{x}_0)^{\mathrm{T}}\boldsymbol{\phi}(\boldsymbol{x}_0) \\
&= o\left(n^{\kappa^*-1}\right)\boldsymbol{\phi}(\boldsymbol{x}_0)^{\mathrm{T}}\boldsymbol{\phi}(\boldsymbol{x}_0).
\end{aligned} \tag{S63}$$

Plugging equations (S62) and (S63) into equation (S61), we have

$$I^{\ddagger}_{n2} = o_P\left(n^{\kappa^*-1}\right)\boldsymbol{\phi}(\boldsymbol{x}_0)^{\mathrm{T}}\boldsymbol{\phi}(\boldsymbol{x}_0). \tag{S64}$$

By equations (S59), (S60), and (S64),

$$\boldsymbol{\phi}(\boldsymbol{x}_0)^{\mathrm{T}}\boldsymbol{\Xi}_2\boldsymbol{\phi}(\boldsymbol{x}_0) = o_P\left(n^{\kappa^*-1}\right)\boldsymbol{\phi}(\boldsymbol{x}_0)^{\mathrm{T}}\boldsymbol{\phi}(\boldsymbol{x}_0),$$

combined with equation (S58), show that $\boldsymbol{\phi}(\boldsymbol{x}_0)^{\mathrm{T}}\boldsymbol{\Xi}_2\boldsymbol{\phi}(\boldsymbol{x}_0)$ is dominated by $\boldsymbol{\phi}(\boldsymbol{x}_0)^{\mathrm{T}}\boldsymbol{\Xi}_1\boldsymbol{\phi}(\boldsymbol{x}_0)$,

moreover, $\mathrm{Var}_r\{\widehat{\tau}_{\mathrm{rct}}(\boldsymbol{x}_0)\} \asymp n^{\kappa^*-1}\boldsymbol{\phi}(\boldsymbol{x}_0)^{\mathrm{T}}\boldsymbol{\phi}(\boldsymbol{x}_0)$ and

$$\begin{aligned}
& \mathrm{Var}_r\left\{\widehat{\tau}_{\mathrm{rct}}(\boldsymbol{x}_0)\right\} \\
= \;& \boldsymbol{\phi}(\boldsymbol{x}_0)^{\mathrm{T}} E_r\left\{(\boldsymbol{A}_1^{\mathrm{T}}\boldsymbol{H}_1\boldsymbol{A}_1 + n\gamma_1\boldsymbol{P}_1)^{-1}\boldsymbol{A}_1^{\mathrm{T}}\boldsymbol{H}_1\boldsymbol{A}_1(\boldsymbol{A}_1^{\mathrm{T}}\boldsymbol{H}_1\boldsymbol{A}_1 + n\gamma_1\boldsymbol{P}_1)^{-1}\right\}\boldsymbol{\phi}(\boldsymbol{x}_0) \\
& + o_P\left(n^{\kappa^*-1}\right)\boldsymbol{\phi}(\boldsymbol{x}_0)^{\mathrm{T}}\boldsymbol{\phi}(\boldsymbol{x}_0). \qquad \text{(S65)}
\end{aligned}$$

By Conditions S15–S16, and equation (S57),

$$\begin{aligned}
& n\gamma_1\boldsymbol{\phi}(\boldsymbol{x}_0)^{\mathrm{T}}(\boldsymbol{A}_1^{\mathrm{T}}\boldsymbol{H}_1\boldsymbol{A}_1 + n\gamma_1\boldsymbol{P}_1)^{-1}\boldsymbol{P}_1(\boldsymbol{A}_1^{\mathrm{T}}\boldsymbol{H}_1\boldsymbol{A}_1 + n\gamma_1\boldsymbol{P}_1)^{-1}\boldsymbol{\phi}(\boldsymbol{x}_0) \\
\leqslant \;& n\gamma_1\rho_{\max}(\boldsymbol{P}_1)\rho_{\max}\left\{(\boldsymbol{A}_1^{\mathrm{T}}\boldsymbol{H}_1\boldsymbol{A}_1 + n\gamma_1\boldsymbol{P}_1)^{-1}\right\}^2\boldsymbol{\phi}(\boldsymbol{x}_0)^{\mathrm{T}}\boldsymbol{\phi}(\boldsymbol{x}_0) \\
\leqslant \;& O(1)n\gamma_1 n^{\kappa^\dagger}n^{2(\kappa^*-1)}\boldsymbol{\phi}(\boldsymbol{x}_0)^{\mathrm{T}}\boldsymbol{\phi}(\boldsymbol{x}_0) \\
= \;& o(n^{\kappa^*-1})\boldsymbol{\phi}(\boldsymbol{x}_0)^{\mathrm{T}}\boldsymbol{\phi}(\boldsymbol{x}_0), \\
& n\gamma_1\boldsymbol{\phi}(\boldsymbol{x}_0)^{\mathrm{T}}(\boldsymbol{A}_1^{\mathrm{T}}\boldsymbol{H}_1\boldsymbol{A}_1)^{-1}\boldsymbol{P}_1(\boldsymbol{A}_1^{\mathrm{T}}\boldsymbol{H}_1\boldsymbol{A}_1 + n\gamma_1\boldsymbol{P}_1)^{-1}\boldsymbol{\phi}(\boldsymbol{x}_0) \\
\leqslant \;& n\gamma_1\rho_{\max}(\boldsymbol{P}_1)\rho_{\max}\left\{(\boldsymbol{A}_1^{\mathrm{T}}\boldsymbol{H}_1\boldsymbol{A}_1)^{-1}\right\}\rho_{\max}\left\{(\boldsymbol{A}_1^{\mathrm{T}}\boldsymbol{H}_1\boldsymbol{A}_1 + n\gamma_1\boldsymbol{P}_1)^{-1}\right\}\boldsymbol{\phi}(\boldsymbol{x}_0)^{\mathrm{T}}\boldsymbol{\phi}(\boldsymbol{x}_0) \\
= \;& o(n^{\kappa^*-1})\boldsymbol{\phi}(\boldsymbol{x}_0)^{\mathrm{T}}\boldsymbol{\phi}(\boldsymbol{x}_0),
\end{aligned}$$

then equation (S65) is deduced by

$$\begin{aligned}
& \mathrm{Var}_r\left\{\widehat{\tau}_{\mathrm{rct}}(\boldsymbol{x}_0)\right\} \\
= \; & \boldsymbol{\phi}(\boldsymbol{x}_0)^{\mathrm{T}} E_r\big\{(\boldsymbol{A}_1^{\mathrm{T}}\boldsymbol{H}_1\boldsymbol{A}_1+n\gamma_1\boldsymbol{P}_1)^{-1}\boldsymbol{A}_1^{\mathrm{T}}\boldsymbol{H}_1(\boldsymbol{A}_1+n\gamma_1\boldsymbol{P}_1-n\gamma_1\boldsymbol{P}_1) \\
& \times(\boldsymbol{A}_1^{\mathrm{T}}\boldsymbol{H}_1\boldsymbol{A}_1+n\gamma_1\boldsymbol{P}_1)^{-1}\big\}\boldsymbol{\phi}(\boldsymbol{x}_0)+o_P\left(n^{\kappa^*-1}\right)\boldsymbol{\phi}(\boldsymbol{x}_0)^{\mathrm{T}}\boldsymbol{\phi}(\boldsymbol{x}_0) \\
= \; & \boldsymbol{\phi}(\boldsymbol{x}_0)^{\mathrm{T}} E_r\big\{(\boldsymbol{A}_1^{\mathrm{T}}\boldsymbol{H}_1\boldsymbol{A}_1+n\gamma_1\boldsymbol{P}_1)^{-1}\big\}\boldsymbol{\phi}(\boldsymbol{x}_0) \\
& -n\gamma_1\boldsymbol{\phi}(\boldsymbol{x}_0)^{\mathrm{T}} E_r\big\{(\boldsymbol{A}_1^{\mathrm{T}}\boldsymbol{H}_1\boldsymbol{A}_1+n\gamma_1\boldsymbol{P}_1)^{-1}\boldsymbol{P}_1(\boldsymbol{A}_1^{\mathrm{T}}\boldsymbol{H}_1\boldsymbol{A}_1+n\gamma_1\boldsymbol{P}_1)^{-1}\big\}\boldsymbol{\phi}(\boldsymbol{x}_0) \\
& +o_P\left(n^{\kappa^*-1}\right)\boldsymbol{\phi}(\boldsymbol{x}_0)^{\mathrm{T}}\boldsymbol{\phi}(\boldsymbol{x}_0) \\
= \; & \boldsymbol{\phi}(\boldsymbol{x}_0)^{\mathrm{T}} E_r\big\{(\boldsymbol{A}_1^{\mathrm{T}}\boldsymbol{H}_1\boldsymbol{A}_1+n\gamma_1\boldsymbol{P}_1)^{-1}\big\}\boldsymbol{\phi}(\boldsymbol{x}_0)+o_P\left(n^{\kappa^*-1}\right)\boldsymbol{\phi}(\boldsymbol{x}_0)^{\mathrm{T}}\boldsymbol{\phi}(\boldsymbol{x}_0) \\
= \; & \boldsymbol{\phi}(\boldsymbol{x}_0)^{\mathrm{T}} E_r\big\{(\boldsymbol{A}_1^{\mathrm{T}}\boldsymbol{H}_1\boldsymbol{A}_1)^{-1}\big\}\boldsymbol{\phi}(\boldsymbol{x}_0)+o_P\left(n^{\kappa^*-1}\right)\boldsymbol{\phi}(\boldsymbol{x}_0)^{\mathrm{T}}\boldsymbol{\phi}(\boldsymbol{x}_0) \\
& -n\gamma_1\boldsymbol{\phi}(\boldsymbol{x}_0)^{\mathrm{T}} E_r\big\{(\boldsymbol{A}_1^{\mathrm{T}}\boldsymbol{H}_1\boldsymbol{A}_1)^{-1}\boldsymbol{P}_1(\boldsymbol{A}_1^{\mathrm{T}}\boldsymbol{H}_1\boldsymbol{A}_1+n\gamma_1\boldsymbol{P}_1)^{-1}\big\}\boldsymbol{\phi}(\boldsymbol{x}_0) \\
= \; & \boldsymbol{\phi}(\boldsymbol{x}_0)^{\mathrm{T}} E_r\big\{(\boldsymbol{A}_1^{\mathrm{T}}\boldsymbol{H}_1\boldsymbol{A}_1)^{-1}\big\}\boldsymbol{\phi}(\boldsymbol{x}_0)+o_P\left(n^{\kappa^*-1}\right)\boldsymbol{\phi}(\boldsymbol{x}_0)^{\mathrm{T}}\boldsymbol{\phi}(\boldsymbol{x}_0). \qquad \text{(S66)}
\end{aligned}$$

Mimicking the discussion of equation (S66), we can also conclude that

$$\begin{aligned}
& \mathrm{Var}_r\left\{\omega_1\widehat{\tau}_n(\boldsymbol{x}_0)+\omega_2\widehat{\lambda}_n(\boldsymbol{x}_0)\right\} \\
= \; & (\omega_1\boldsymbol{\phi}(\boldsymbol{x}_0)^{\mathrm{T}},\omega_2\boldsymbol{\psi}(\boldsymbol{x}_0)^{\mathrm{T}})E_r\left\{(\boldsymbol{A}^{\mathrm{T}}\boldsymbol{H}\boldsymbol{A})^{-1}\right\}(\omega_1\boldsymbol{\phi}(\boldsymbol{x}_0)^{\mathrm{T}},\omega_2\boldsymbol{\psi}(\boldsymbol{x}_0)^{\mathrm{T}})^{\mathrm{T}} \\
& +o_P\left(n^{\kappa^*-1}\right)(\omega_1\boldsymbol{\phi}(\boldsymbol{x}_0)^{\mathrm{T}},\omega_2\boldsymbol{\psi}(\boldsymbol{x}_0)^{\mathrm{T}})(\omega_1\boldsymbol{\phi}(\boldsymbol{x}_0)^{\mathrm{T}},\omega_2\boldsymbol{\psi}(\boldsymbol{x}_0)^{\mathrm{T}})^{\mathrm{T}}
\end{aligned}$$

for every $\omega_1,\omega_2\in\mathbb{R}$. Let

$$\begin{pmatrix} \boldsymbol{\Pi}_1 & \boldsymbol{\Pi}_{12} \\ \boldsymbol{\Pi}_{12}^{\mathrm{T}} & \boldsymbol{\Pi}_2 \end{pmatrix} = (\boldsymbol{A}^{\mathrm{T}}\boldsymbol{H}\boldsymbol{A})^{-1} = \begin{pmatrix} \boldsymbol{A}_1^{\mathrm{T}}\boldsymbol{H}_1\boldsymbol{A}_1+\boldsymbol{A}_{21}^{\mathrm{T}}\boldsymbol{H}_2\boldsymbol{A}_{21} & \boldsymbol{A}_{21}^{\mathrm{T}}\boldsymbol{H}_2\boldsymbol{A}_2 \\ \boldsymbol{A}_2^{\mathrm{T}}\boldsymbol{H}_2\boldsymbol{A}_{21} & \boldsymbol{A}_2^{\mathrm{T}}\boldsymbol{H}_2\boldsymbol{A}_2 \end{pmatrix}^{-1}. \qquad \text{(S67)}$$

Taking $\omega_1=1$ and $\omega_2=0$, we have

$$\mathrm{Var}_r\{\widehat{\tau}_n(\boldsymbol{x}_0)\}=\boldsymbol{\phi}(\boldsymbol{x}_0)^{\mathrm{T}}E_r\left(\boldsymbol{\Pi}_1\right)\boldsymbol{\phi}(\boldsymbol{x}_0)+o_P\left(n^{\kappa^*-1}\right)\boldsymbol{\phi}(\boldsymbol{x}_0)^{\mathrm{T}}\boldsymbol{\phi}(\boldsymbol{x}_0). \qquad \text{(S68)}$$

By equation (S67) and some routine calculation,

$$\boldsymbol{\Pi}_1=\left\{\boldsymbol{A}_1^{\mathrm{T}}\boldsymbol{H}_1\boldsymbol{A}_1+\boldsymbol{A}_{21}^{\mathrm{T}}\boldsymbol{H}_2\boldsymbol{A}_{21}-\boldsymbol{A}_{21}^{\mathrm{T}}\boldsymbol{H}_2\boldsymbol{A}_2(\boldsymbol{A}_2^{\mathrm{T}}\boldsymbol{H}_2\boldsymbol{A}_2)^{-1}\boldsymbol{A}_2^{\mathrm{T}}\boldsymbol{H}_2\boldsymbol{A}_{21}\right\}^{-1}.$$

To prove Theorem 4.4, it is enough to show that $\mathbf{\Pi}_1 \leqslant (\boldsymbol{A}_1^{\mathrm{T}}\boldsymbol{H}_1\boldsymbol{A}_1)^{-1}$, that is

$$\begin{aligned} & \mathbf{\Pi}_1^{-1} - \boldsymbol{A}_1^{\mathrm{T}}\boldsymbol{H}_1\boldsymbol{A}_1 \\ = \ & \boldsymbol{A}_{21}^{\mathrm{T}}\boldsymbol{H}_2\boldsymbol{A}_{21} - \boldsymbol{A}_{21}^{\mathrm{T}}\boldsymbol{H}_2\boldsymbol{A}_2(\boldsymbol{A}_2^{\mathrm{T}}\boldsymbol{H}_2\boldsymbol{A}_2)^{-1}\boldsymbol{A}_2^{\mathrm{T}}\boldsymbol{H}_2\boldsymbol{A}_{21} \\ \geqslant \ & \mathbf{0}. \end{aligned} \tag{S69}$$

Let $\boldsymbol{A}_2^* = \boldsymbol{H}_2^{1/2}\boldsymbol{A}_2(\boldsymbol{A}_2^{\mathrm{T}}\boldsymbol{H}_2\boldsymbol{A}_2)^{-1}\boldsymbol{A}_2^{\mathrm{T}}\boldsymbol{H}_2^{1/2}$, then we have $(\boldsymbol{A}_2^*)^2 = \boldsymbol{A}_2^*$, which implies that $\rho_{\max}(\boldsymbol{A}_2^*) = 1$. Therefore, for every $\boldsymbol{\omega} = (\omega_1, \ldots, \omega_{r_1})^{\mathrm{T}}$,

$$\begin{aligned} & \boldsymbol{\omega}^{\mathrm{T}}\boldsymbol{A}_{21}^{\mathrm{T}}\boldsymbol{H}_2\boldsymbol{A}_2(\boldsymbol{A}_2^{\mathrm{T}}\boldsymbol{H}_2\boldsymbol{A}_2)^{-1}\boldsymbol{A}_2^{\mathrm{T}}\boldsymbol{H}_2\boldsymbol{A}_{21}\boldsymbol{\omega} \\ = \ & (\boldsymbol{H}_2^{1/2}\boldsymbol{A}_{21}\boldsymbol{\omega})^{\mathrm{T}}\boldsymbol{A}_2^*\boldsymbol{H}_2^{1/2}\boldsymbol{A}_{21}\boldsymbol{\omega} \\ \leqslant \ & \rho_{\max}(\boldsymbol{A}_2^*)(\boldsymbol{H}_2^{1/2}\boldsymbol{A}_{21}\boldsymbol{\omega})^{\mathrm{T}}\boldsymbol{H}_2^{1/2}\boldsymbol{A}_{21}\boldsymbol{\omega} \\ = \ & \boldsymbol{\omega}^{\mathrm{T}}\boldsymbol{A}_{21}^{\mathrm{T}}\boldsymbol{H}_2\boldsymbol{A}_{21}\boldsymbol{\omega}, \end{aligned}$$

which proves equation (S69). Consequently,

$$\mathrm{Var}_r\{\widehat{\tau}_{\mathrm{rct}}(\boldsymbol{x}_0)\} \geqslant \mathrm{Var}_r\{\widehat{\tau}_n(\boldsymbol{x}_0)\} + o_P\left(n^{\kappa^*-1}\right)\boldsymbol{\phi}(\boldsymbol{x}_0)^{\mathrm{T}}\boldsymbol{\phi}(\boldsymbol{x}_0).$$

Specifically,

$$\begin{aligned} \mathrm{Var}_r\{\widehat{\tau}_{\mathrm{rct}}(\boldsymbol{x}_0)\} &= \boldsymbol{\phi}(\boldsymbol{x}_0)^{\mathrm{T}}E_r(\mathbf{\Sigma}_{\mathrm{rct}})\boldsymbol{\phi}(\boldsymbol{x}_0) + o_P\left(n^{\kappa^*-1}\right)\boldsymbol{\phi}(\boldsymbol{x}_0)^{\mathrm{T}}\boldsymbol{\phi}(\boldsymbol{x}_0), \\ \mathrm{Var}_r\{\widehat{\tau}_n(\boldsymbol{x}_0)\} &= \boldsymbol{\phi}(\boldsymbol{x}_0)^{\mathrm{T}}E_r(\mathbf{\Sigma}_{\mathrm{int}})\boldsymbol{\phi}(\boldsymbol{x}_0) + o_P\left(n^{\kappa^*-1}\right)\boldsymbol{\phi}(\boldsymbol{x}_0)^{\mathrm{T}}\boldsymbol{\phi}(\boldsymbol{x}_0), \end{aligned}$$

where

$$\begin{aligned} \mathbf{\Sigma}_{\mathrm{rct}} &= \left(\boldsymbol{A}_1^{\mathrm{T}}\boldsymbol{H}_1\boldsymbol{A}_1\right)^{-1}, \\ \mathbf{\Sigma}_{\mathrm{int}} &= \left(\boldsymbol{A}_1^{\mathrm{T}}\boldsymbol{H}_1\boldsymbol{A}_1 + \boldsymbol{A}_{21}^{\mathrm{T}}\boldsymbol{H}_2\boldsymbol{A}_{21} - \boldsymbol{A}_{21}^{\mathrm{T}}\boldsymbol{H}_2\boldsymbol{A}_2(\boldsymbol{A}_2^{\mathrm{T}}\boldsymbol{H}_2\boldsymbol{A}_2)^{-1}\boldsymbol{A}_2^{\mathrm{T}}\boldsymbol{H}_2\boldsymbol{A}_{21}\right)^{-1}, \end{aligned}$$

and

$$\mathbf{\Sigma}_{\mathrm{int}}^{-1} - \mathbf{\Sigma}_{\mathrm{rct}}^{-1} = \boldsymbol{A}_{21}^{\mathrm{T}}\boldsymbol{H}_2\boldsymbol{A}_{21} - \boldsymbol{A}_{21}^{\mathrm{T}}\boldsymbol{H}_2\boldsymbol{A}_2(\boldsymbol{A}_2^{\mathrm{T}}\boldsymbol{H}_2\boldsymbol{A}_2)^{-1}\boldsymbol{A}_2^{\mathrm{T}}\boldsymbol{H}_2\boldsymbol{A}_{21} \geqslant \mathbf{0}.$$

Thus, we conclude Theorem 4.

Web Appendix F: Additional simulation results

F.1 Simulation results of Case 2 and Case 3 in Section 5.1 of the main paper

[Figure 1 about here.]

[Figure 2 about here.]

[Figure 3 about here.]

[Figure 4 about here.]

F.2 Additional simulation study

Here, we consider the case where $p = 4$, i.e., $\boldsymbol{X} = (X_1, X_2, X_3, X_4)^{\mathrm{T}}$, where X_1 and X_2 are continuous, and X_3 and X_4 are binary. The estimation methodology employed in this additional simulation is the same as the approach detailed in the main paper. Specifically, we stratify the data into four distinct groups based on the values of the covariates X_3 and X_4. Given that both X_3 and X_4 are binary, this stratification results in four possible combinations: $(X_3 = 0, X_4 = 0)$, $(X_3 = 1, X_4 = 0)$, $(X_3 = 0, X_4 = 1)$, and $(X_3 = 1, X_4 = 1)$. For each of these four groups, applying the same method as described in the main paper, we can get the results of RCT data-based only method and those of the RWD-based only method. However, it is important to note that in the implementation of the proposed integrative method, we stratify the RCT data into four groups based on the values of X_3 and X_4. In contrast, the RWD is not stratified, instead, the entire dataset is used for combining each of four groups from the RCT data. Specifically, the information from X_3 and X_4 in the RWD is not utilized. For the RWD, the covariates information we used is only from the continuous covariates X_1 and X_2. This further demonstrates that the proposed method allows for inconsistency in data structure between the RCT data and the RWD.

Throughout the simulation, $e(\boldsymbol{X}) = P(A = 1 \mid \boldsymbol{X}, S = 1) = 0.5$ is known. The censoring rate is set around 20% for the RCT data and 70% for the RWD. Corresponding to the

scenarios in the simulation study of the main paper, we also consider three cases, and the specific settings are as follows.

Case S1. The generations of X_u, X_1, and X_2 are the same as in Case 1 of Section 5.1 in the main paper. For the RCT data, X_3 and X_4 are generated from a Bernoulli distribution with a success probability of 0.5. In contrast, for the RWD, X_3 is generated with a success probability of 0.6, and X_4 with a success probability of 0.4. For both the RCT data and the RWD, T is generated from the survival function

$$\begin{aligned} & G_T(t \mid X_1, X_2, X_3, X_4, X_u, A) \\ = \; & (1 + 0.02t) \exp\Big[- 0.1X_u t - 0.845t \exp\big\{ - 0.5A + (0.6A - 0.3) \exp(1.5X_1) \\ & +(0.3 - 0.6A) \exp(1.5X_2) + 0.5X_3 - 0.5X_4 \big\}\Big], \end{aligned}$$

and the censoring time C is generated from a Cox proportional hazards model with the conditional hazard function taking the form $h_C(t \mid X_1, X_2, X_3, X_4) = h_{0C}(t) \exp(0.5X_1 + 0.5X_2 - 0.5X_3 - 0.5X_4)$, where $h_{0C}(t)$ is the baseline hazard function. We set $h_{0C}(t) = 0.052$ with a study duration of 4.5 for the RCT data, and $h_{0C}(t) = 3.164$ with a study duration of 3.5 for the RWD to achieve the preset censoring rates. We consider the restricted time point $L = 2$, the sample size $(n_1, n_0) = (500, 1000)$ and $(1000, 2000)$. For each configuration, 1000 simulations are repeated.

Case S2. For the RCT data, the failure time T is generated from a Cox model

$$\begin{aligned} & h_T(t \mid X_1, X_2, X_3, X_4, A) \\ = \; & 0.845 \exp\big\{ - 0.5A + (0.6A - 0.3) \exp(1.5X_1) \\ & +(0.3 - 0.6A) \exp(1.5X_2) + 0.5X_3 - 0.5X_4 \big\}. \end{aligned}$$

In contrast, for the RWD, X_u is generated from a standard normal distribution and T is

generated from a Cox model

$$
\begin{aligned}
& h_T(t \mid X_1, X_2, X_3, X_4, X_u, A) \\
= \;& 0.845 \exp\big\{ -0.5A + (0.6A - 0.3)\exp(1.5X_1) \\
& +(0.3 - 0.6A)\exp(1.5X_2) + 0.5X_3 - 0.5X_4 + X_u \big\}.
\end{aligned}
$$

We set $h_{0C}(t) = 0.055$ for the RCT data and $h_{0C}(t) = 3.587$ for the RWD. The remaining setups are the same as in Case S1.

Case S3. For both the RCT data and the RWD, T is generated from a Cox model

$$
\begin{aligned}
& h_T(t \mid X_1, X_2, X_3, X_4, X_u, A) \\
= \;& 0.845 \exp\big\{ -0.5A + (0.6A - 0.3)\exp(1.5X_1) \\
& +(0.3 - 0.6A)\exp(1.5X_2) + 0.5X_3 - 0.5X_4 + X_u \big\}.
\end{aligned}
$$

We set $h_{0C}(t) = 0.02$ with a study duration of 5.5 for the RCT data. The remaining setups are the same as in Case S2.

Case S1 corresponds to the situation where the failure time follows Cox type (non-standard Cox proportional hazards model), and $E(T_L \mid \boldsymbol{X}, A, S = 1) = E(T_L \mid \boldsymbol{X}, A, S = 0)$. Case S2 corresponds to the situation where the failure time follows Cox type (non-standard Cox proportional hazards model), but $E(T_L \mid \boldsymbol{X}, A, S = 1) \neq E(T_L \mid \boldsymbol{X}, A, S = 0)$. Case S3 addresses the situation where the failure time does not follow Cox type, and $E(T_L \mid \boldsymbol{X}, A, S = 1) = E(T_L \mid \boldsymbol{X}, A, S = 0)$. The simulation results are summarized in Figures S5–S28, , which allow us to draw conclusions similar to those in Section 5.1 of the main paper. However, the coverage performance in this additional simulation is not as favorable as that in the main paper. This may be attributed to two main reasons. Firstly, the HTE is identified based on the RCT data, which plays a crucial role in the estimation process. In this additional simulation, we stratify the RCT data into four groups and apply the estimation method to each subgroup separately. As a result, the sample size for each subgroup

is significantly reduced, which directly impacts the coverage probability. Secondly, in this additional simulation study, the standard Cox proportional hazards model is used to fit the survival data. Thus, the failure time model is always misspecified, which impacts the coverage performance. Even though, the coverage probability still reaches above 90% at almost all evaluation points. This is a commendable result for a fully nonparametric method dealing with four-dimensional covariates, including two discrete variables, as well as a very complex true function form. It demonstrates the robustness and effectiveness of the proposed integrative method even under less favorable conditions.

[Figure 5 about here.]

[Figure 6 about here.]

[Figure 7 about here.]

[Figure 8 about here.]

[Figure 9 about here.]

[Figure 10 about here.]

[Figure 11 about here.]

[Figure 12 about here.]

[Figure 13 about here.]

[Figure 14 about here.]

[Figure 15 about here.]

[Figure 16 about here.]

[Figure 17 about here.]

[Figure 18 about here.]

[Figure 19 about here.]

[Figure 20 about here.]

[Figure 21 about here.]

[Figure 22 about here.]

[Figure 23 about here.]

[Figure 24 about here.]

[Figure 25 about here.]

[Figure 26 about here.]

[Figure 27 about here.]

[Figure 28 about here.]

References

Cox, D. D. (1984). Multivariate smoothing spline functions. *SIAM Journal on Numerical Analysis* **21**, 789–813.

Cox, D. D. (1988). Approximation of method of regularization estimators. *The Annals of Statistics* **16**, 694–712.

Gu, C. (2013). *Smoothing Spline AVOVA Models.* New York: Springer.

Hoeffding, W. (1963). Probability inequalities for sums of bounded random variables. *Journal of the American Statistical Association* **58**, 13–30.

Liu, Y., Mao, G., and Zhao, X. (2020). Local asymptotic inference for nonparametric regression with censored survival data. *Journal of Nonparametric Statistics* **32**, 1015–1028.

Newey, W. K. (1997). Convergence rates and asymptotic normality for series estimators. *Journal of Econometric* **79**, 147–168.

O'Sullivan, F. (1993). Nonparametric estimation in the Cox model. *The Annals of Statistics* **21**, 124–145.

Shang, Z. and Cheng, G. (2013). Local and global asymptotic inference in smoothing spline models. *The Annals of Statistics* **41**, 2608–2638.

van der Vaart, A. W. (1998). *Asymptotic Statistics.* US: Cambridge University Press.

van der Vaart, A. W. and Wellner, J. A. (1996). *Weak Convergence and Empirical Processes: With Applications to Statistics.* New York: Springer.

Received October 2007. *Revised February* 2008. *Accepted March* 2008.

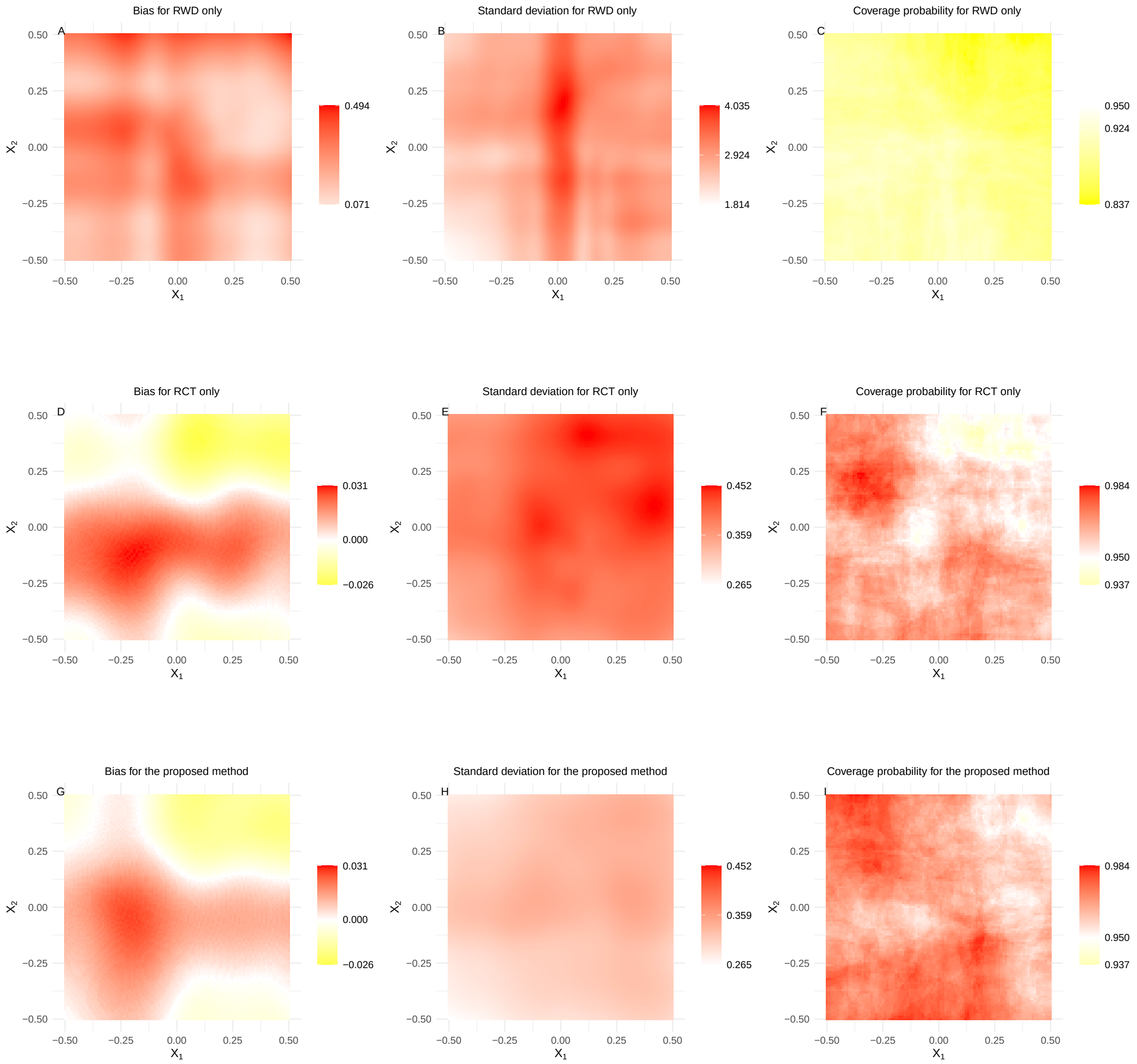

Figure S1. The simulation results of Case 2 with $(n_1, n_0) = (500, 1000)$.

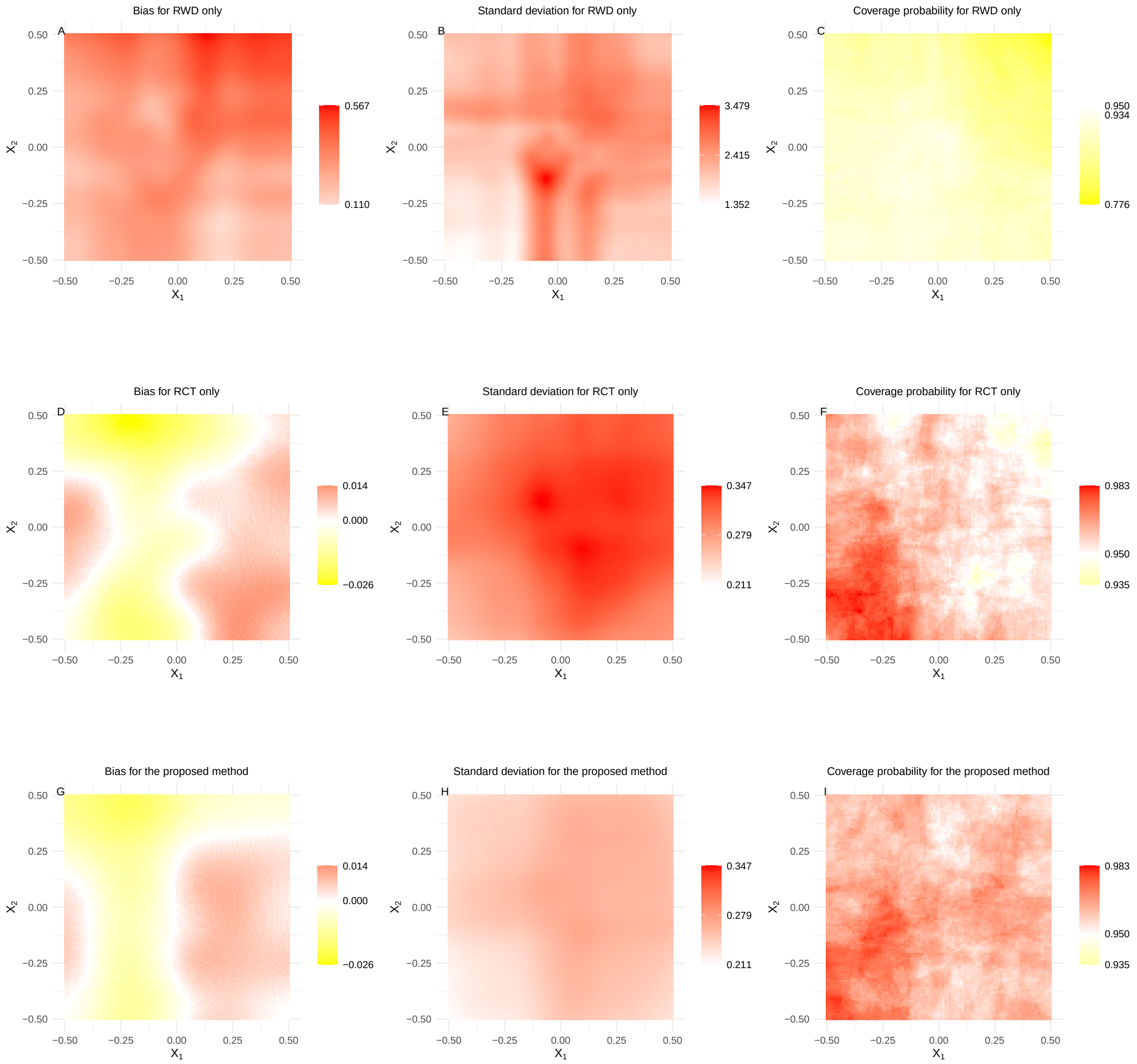

Figure S2. The simulation results of Case 2 with $(n_1, n_0) = (1000, 2000)$.

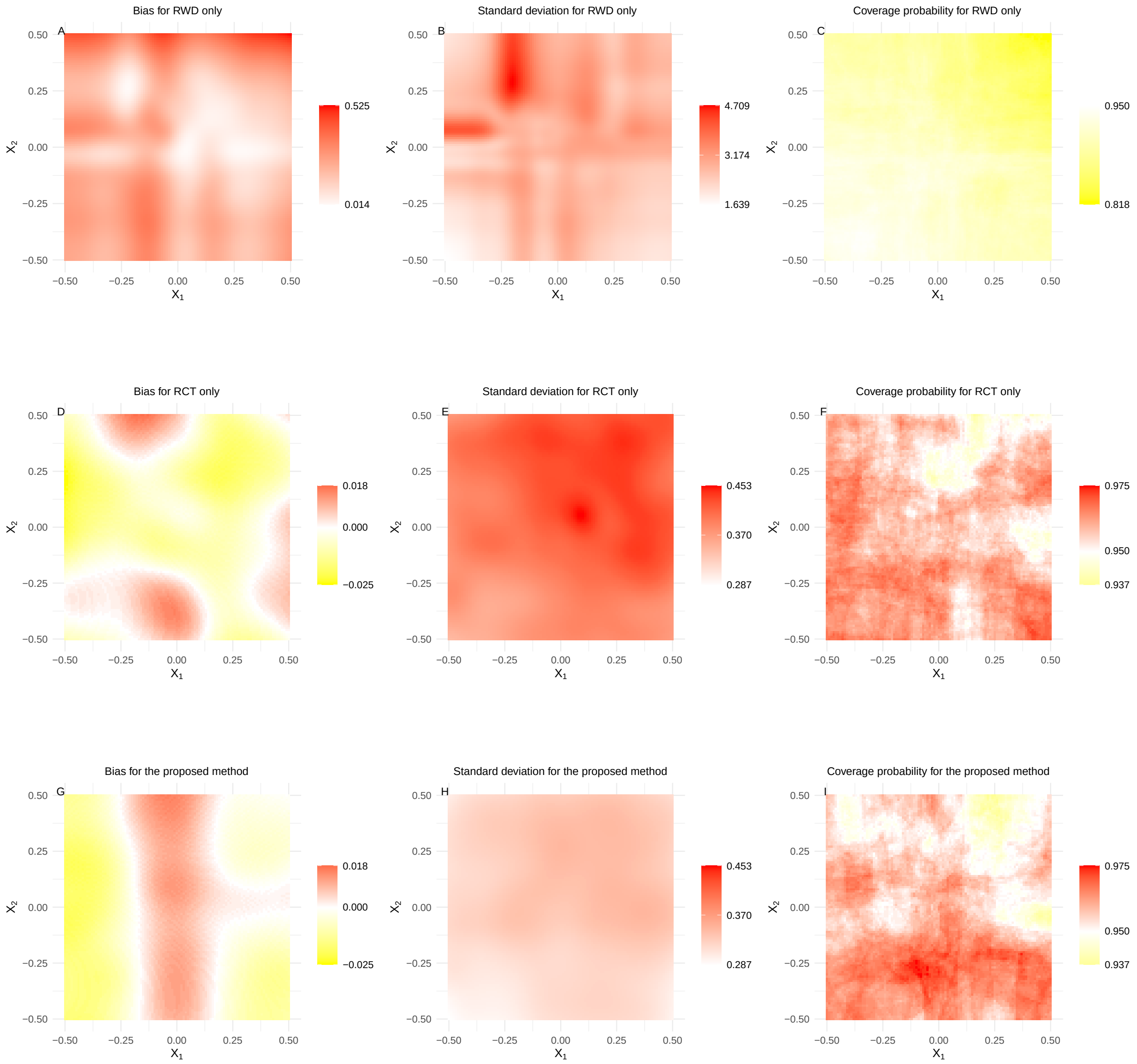

Figure S3. The simulation results of Case 3 with $(n_1, n_0) = (500, 1000)$.

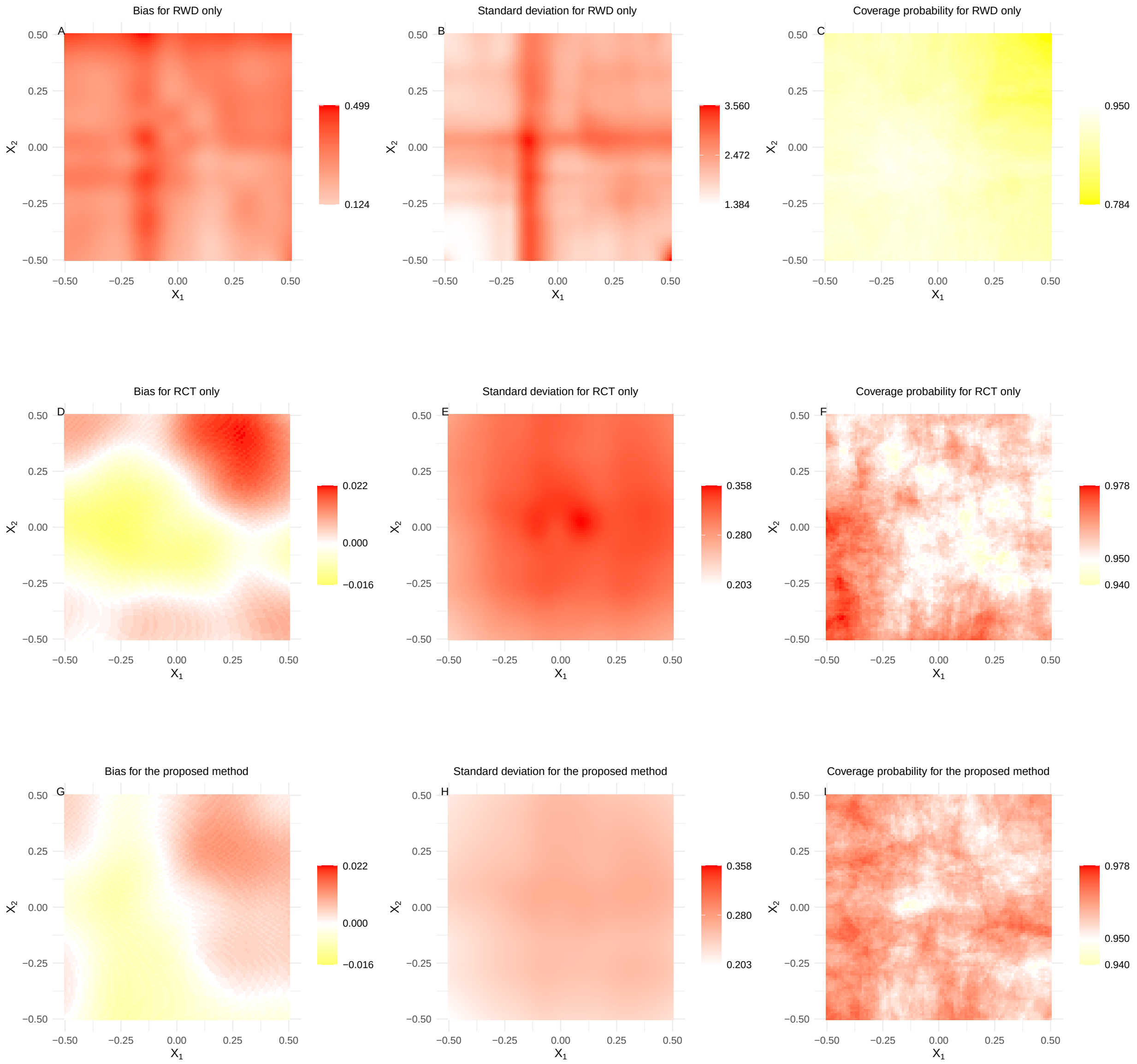

Figure S4. The simulation results of Case 3 with $(n_1, n_0) = (1000, 2000)$.

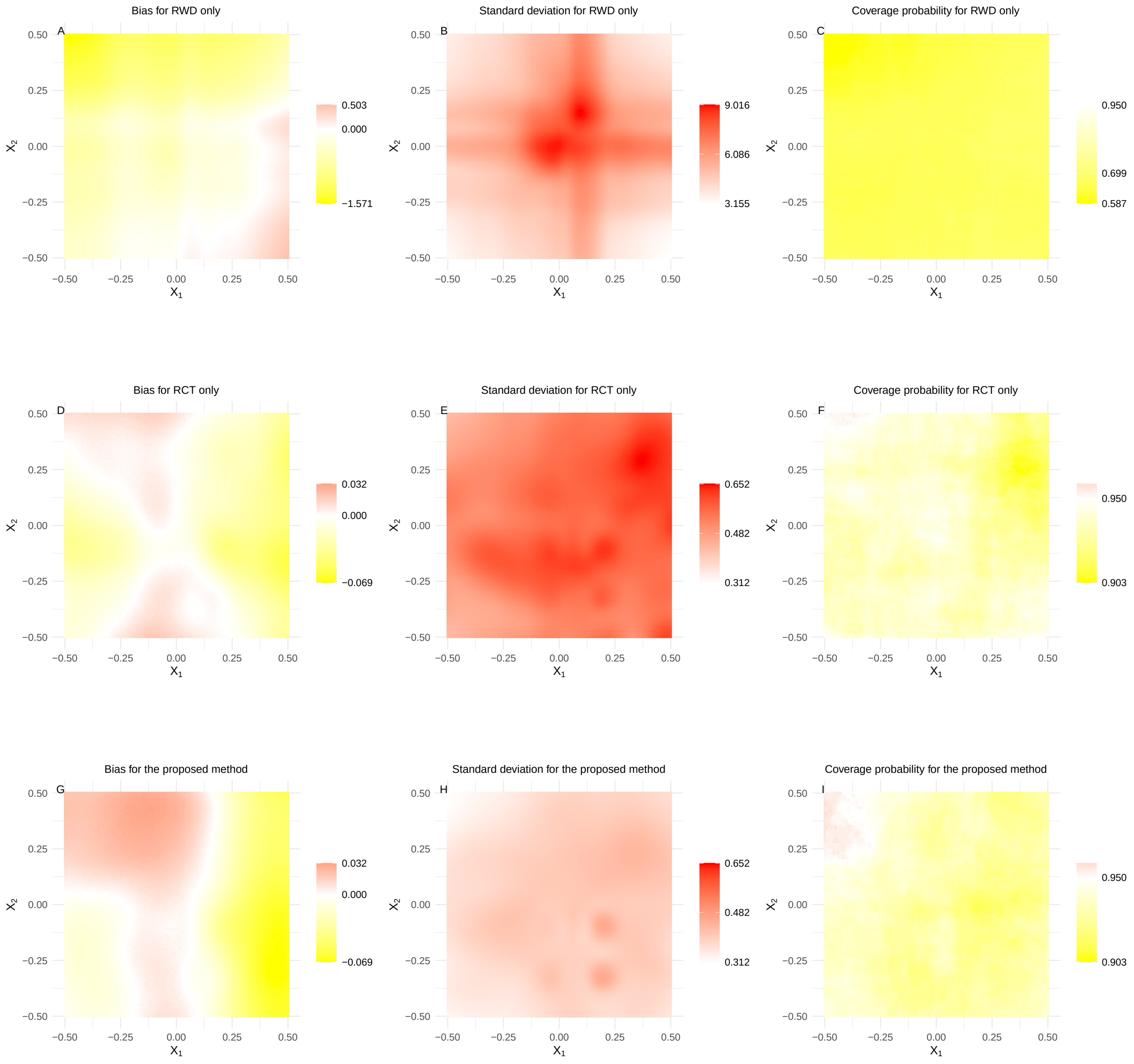

Figure S5. The simulation results of Case S1 with $(n_1, n_0) = (500, 1000)$, $X_3 = 0$, and $X_4 = 0$.

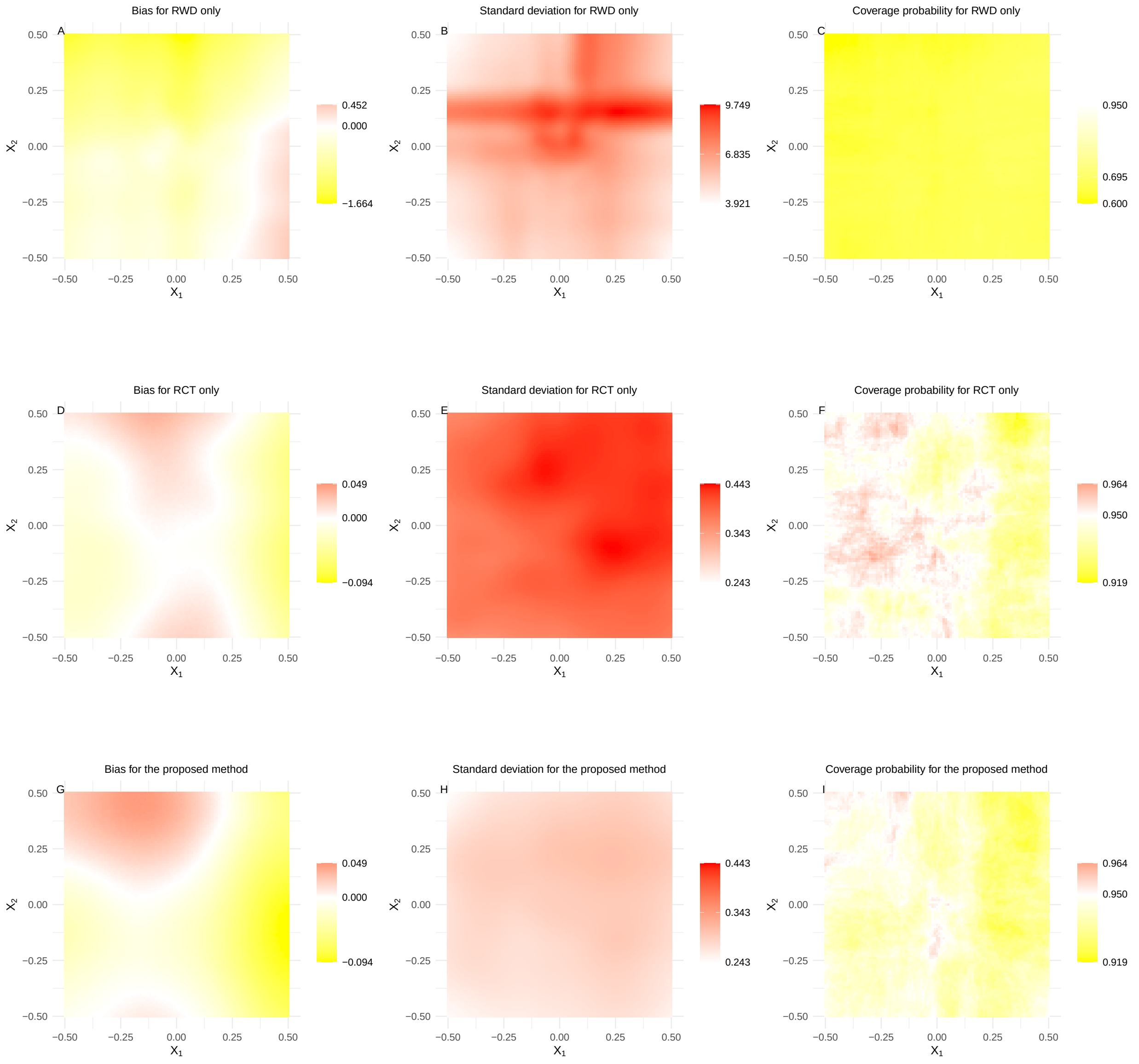

Figure S6. The simulation results of Case S1 with $(n_1, n_0) = (1000, 2000)$, $X_3 = 0$, and $X_4 = 0$.

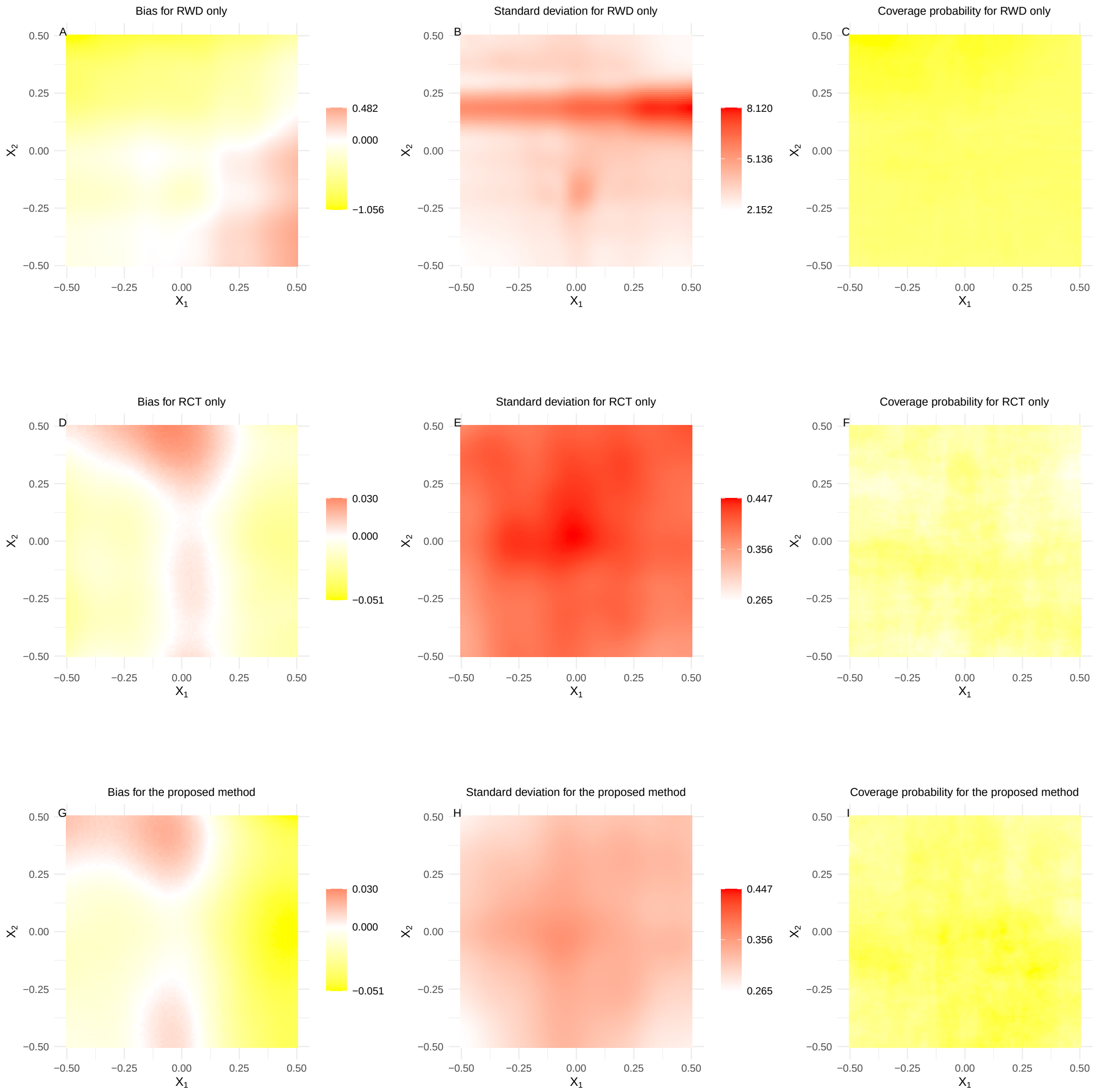

Figure S7. The simulation results of Case S1 with $(n_1, n_0) = (500, 1000)$, $X_3 = 1$, and $X_4 = 0$.

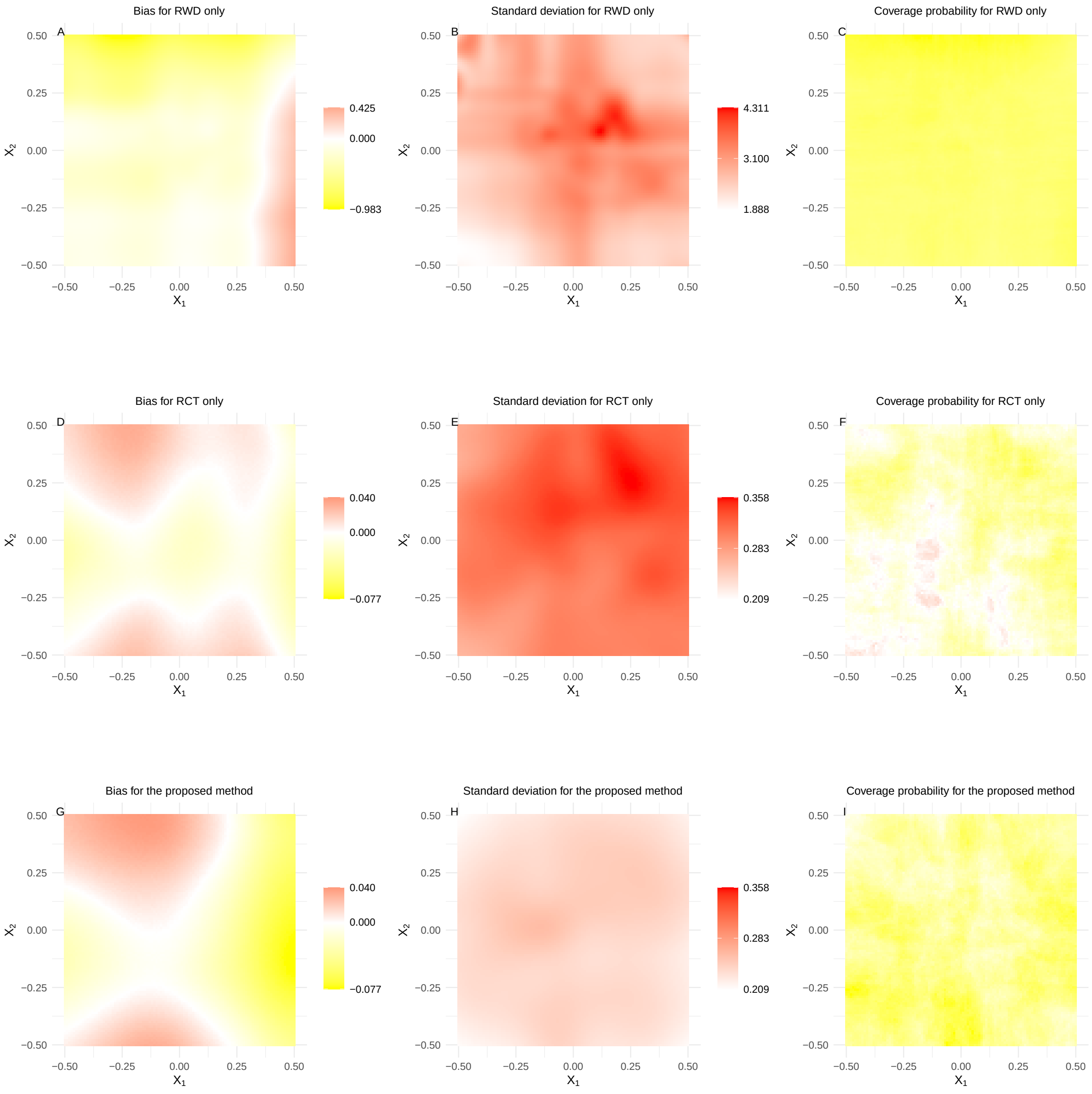

Figure S8. The simulation results of Case S1 with $(n_1, n_0) = (1000, 2000)$, $X_3 = 1$, and $X_4 = 0$.

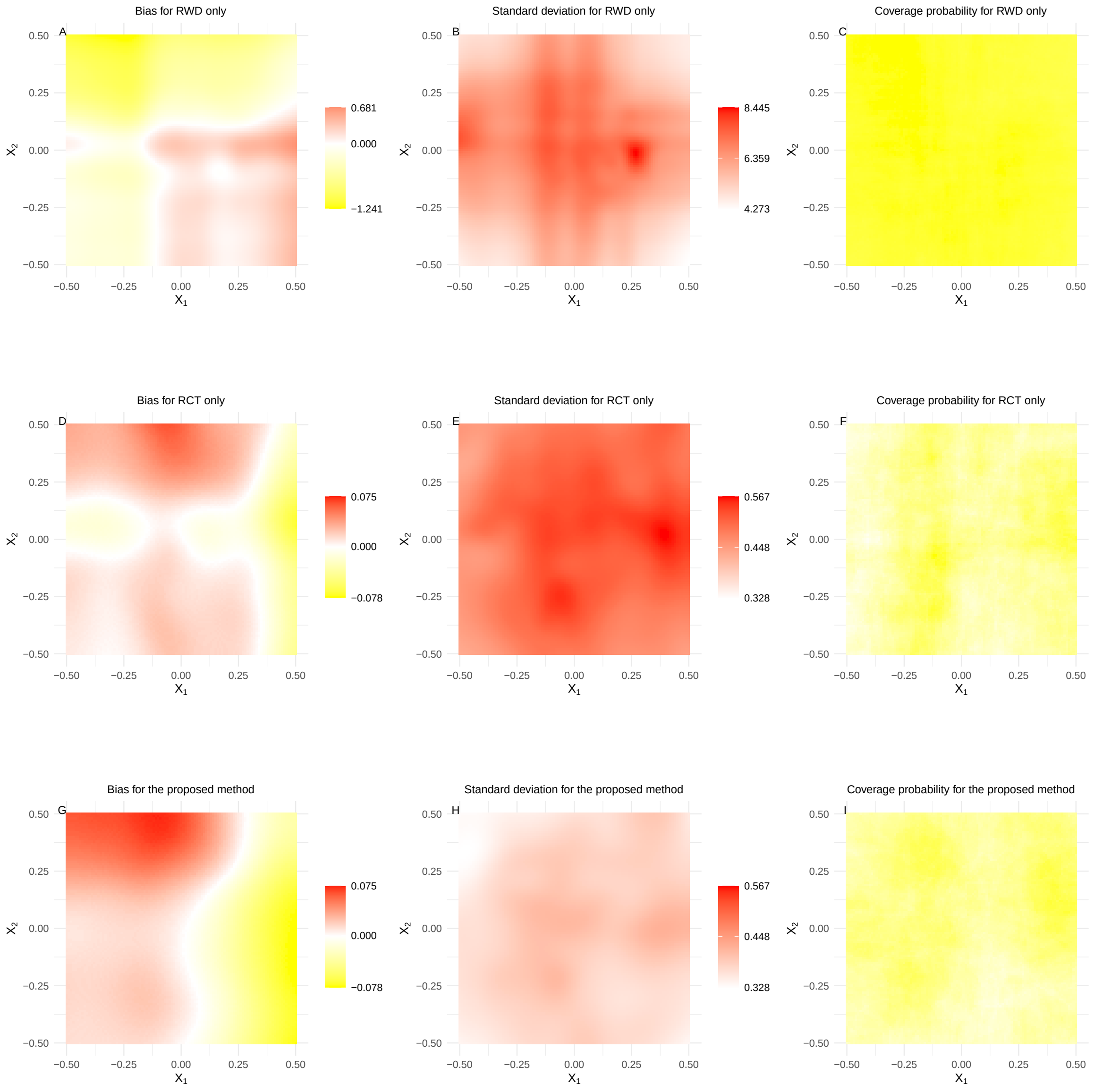

Figure S9. The simulation results of Case S1 with $(n_1, n_0) = (500, 1000)$, $X_3 = 0$, and $X_4 = 1$.

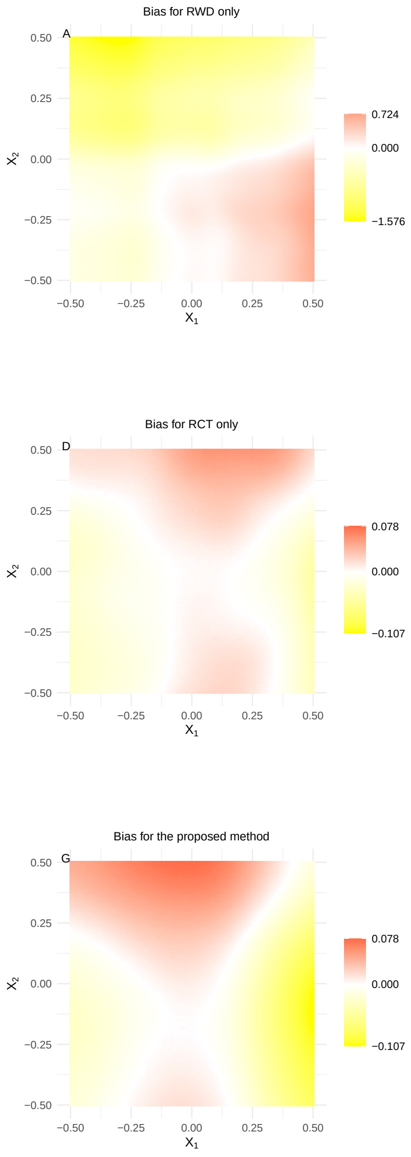

Figure S10. The simulation results of Case S1 with $(n_1, n_0) = (1000, 2000)$, $X_3 = 0$, and $X_4 = 1$.

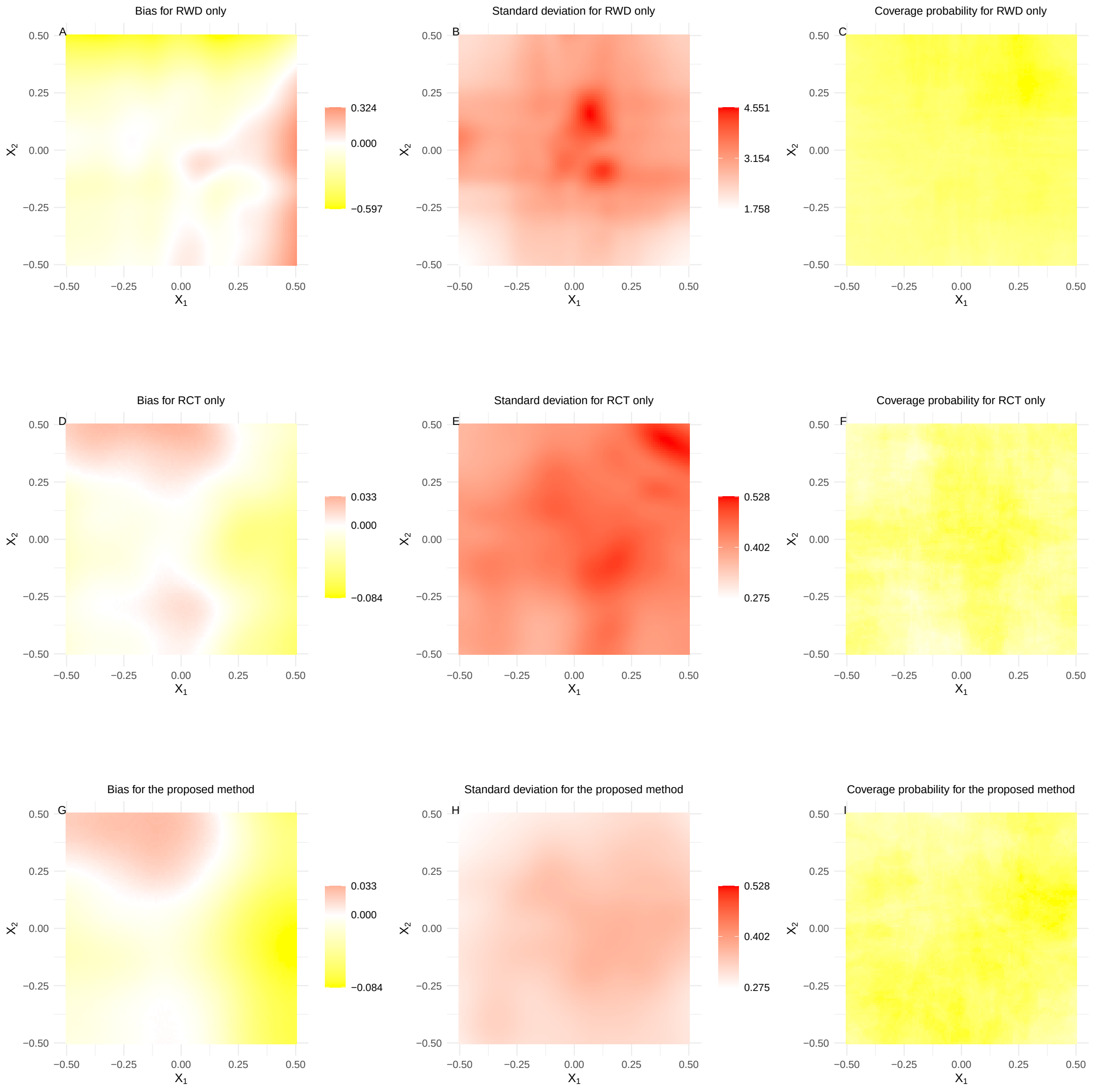

Figure S11. The simulation results of Case S1 with $(n_1, n_0) = (500, 1000)$, $X_3 = 1$, and $X_4 = 1$.

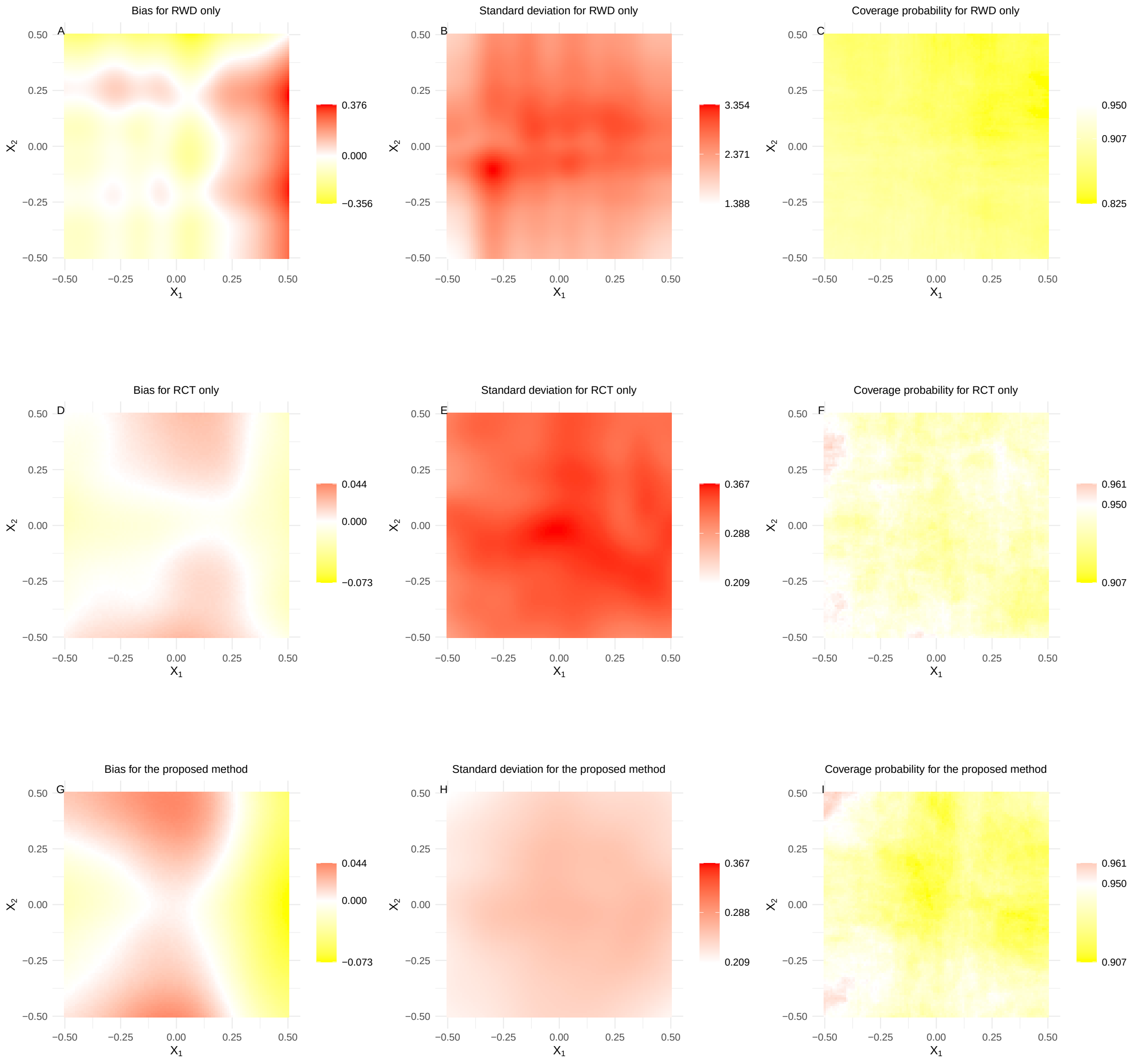

Figure S12. The simulation results of Case S1 with $(n_1, n_0) = (1000, 2000)$, $X_3 = 1$, and $X_4 = 1$.

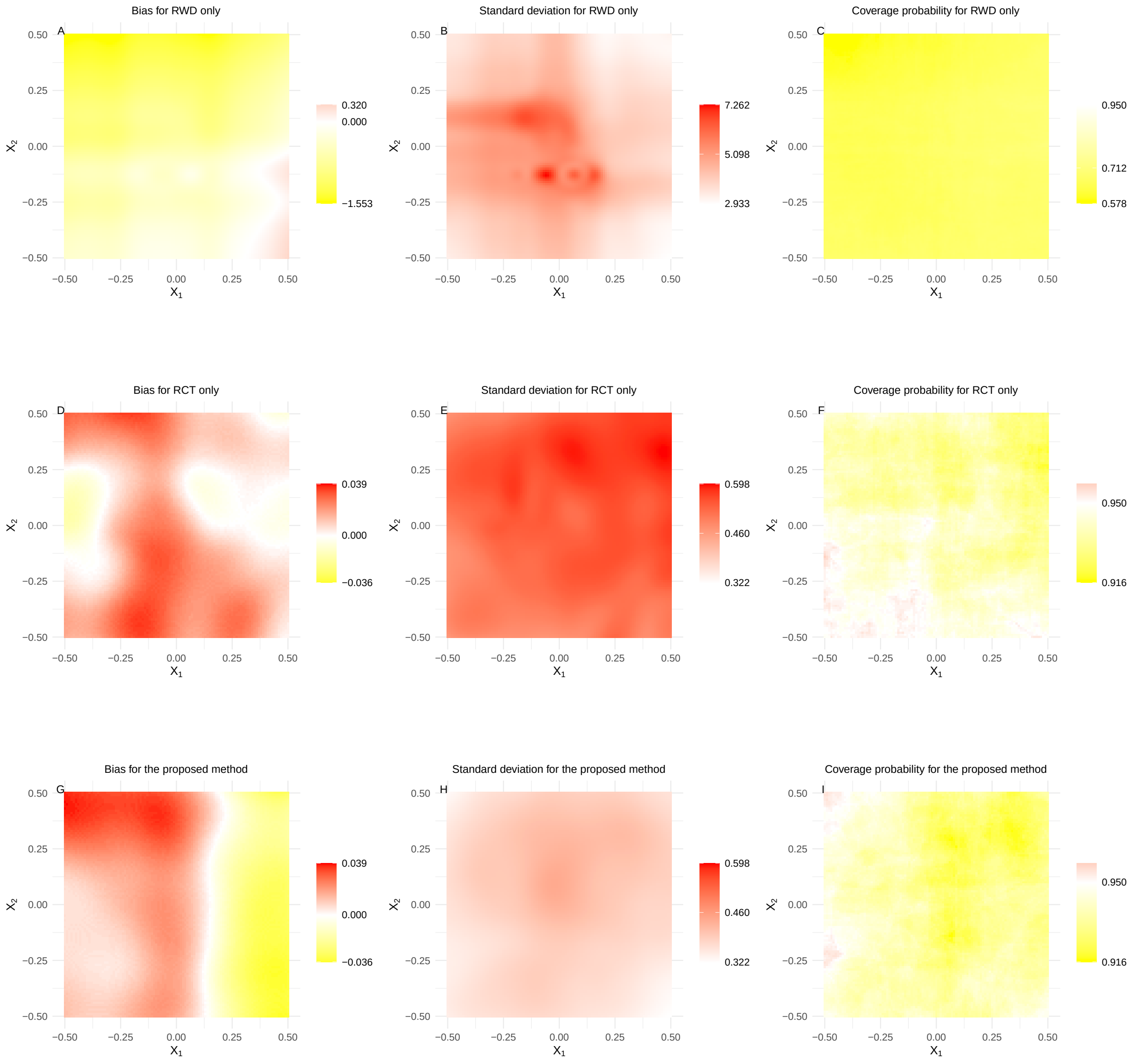

Figure S13. The simulation results of Case S2 with $(n_1, n_0) = (500, 1000)$, $X_3 = 0$, and $X_4 = 0$.

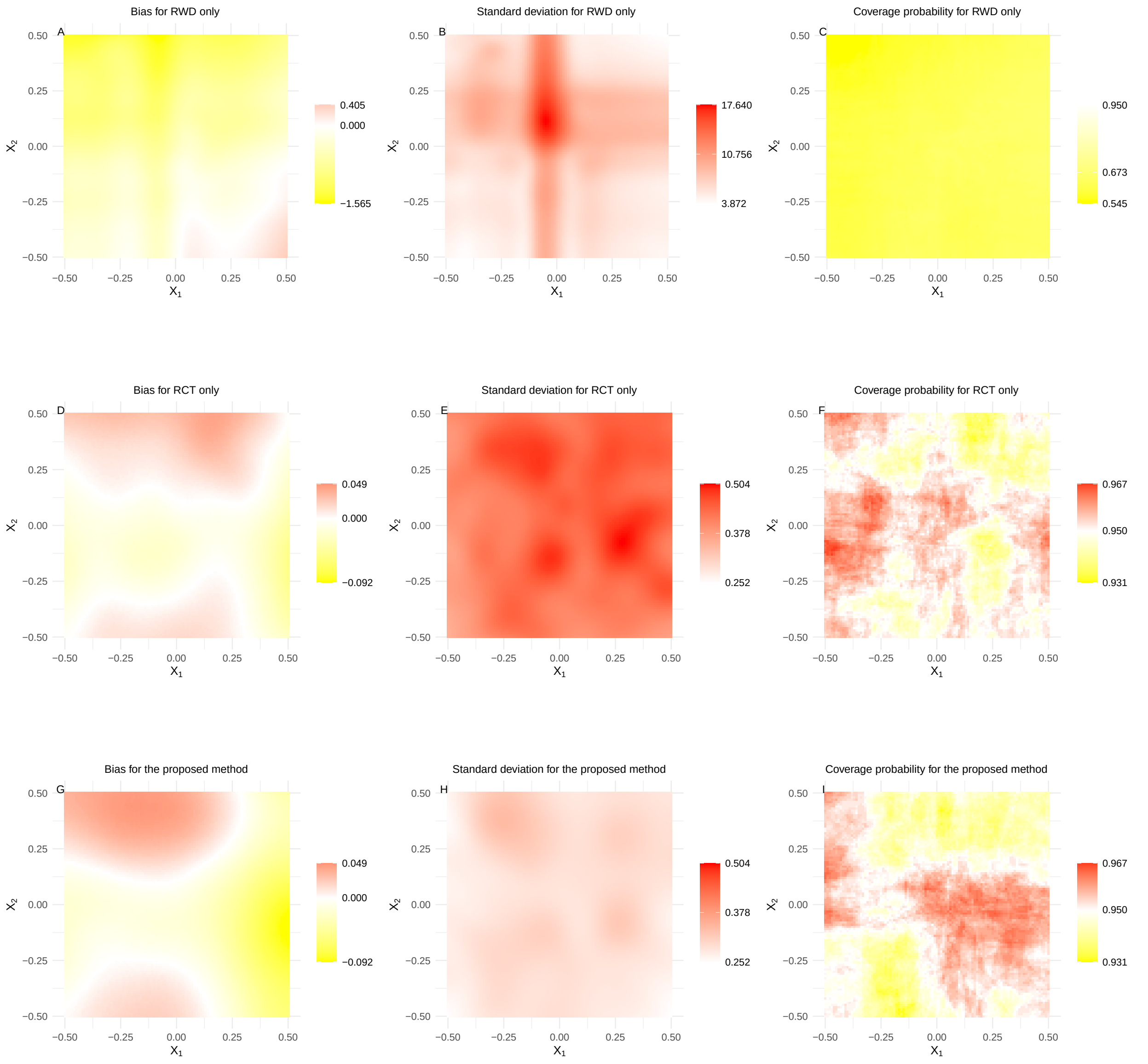

Figure S14. The simulation results of Case S2 with $(n_1, n_0) = (1000, 2000)$, $X_3 = 0$, and $X_4 = 0$.

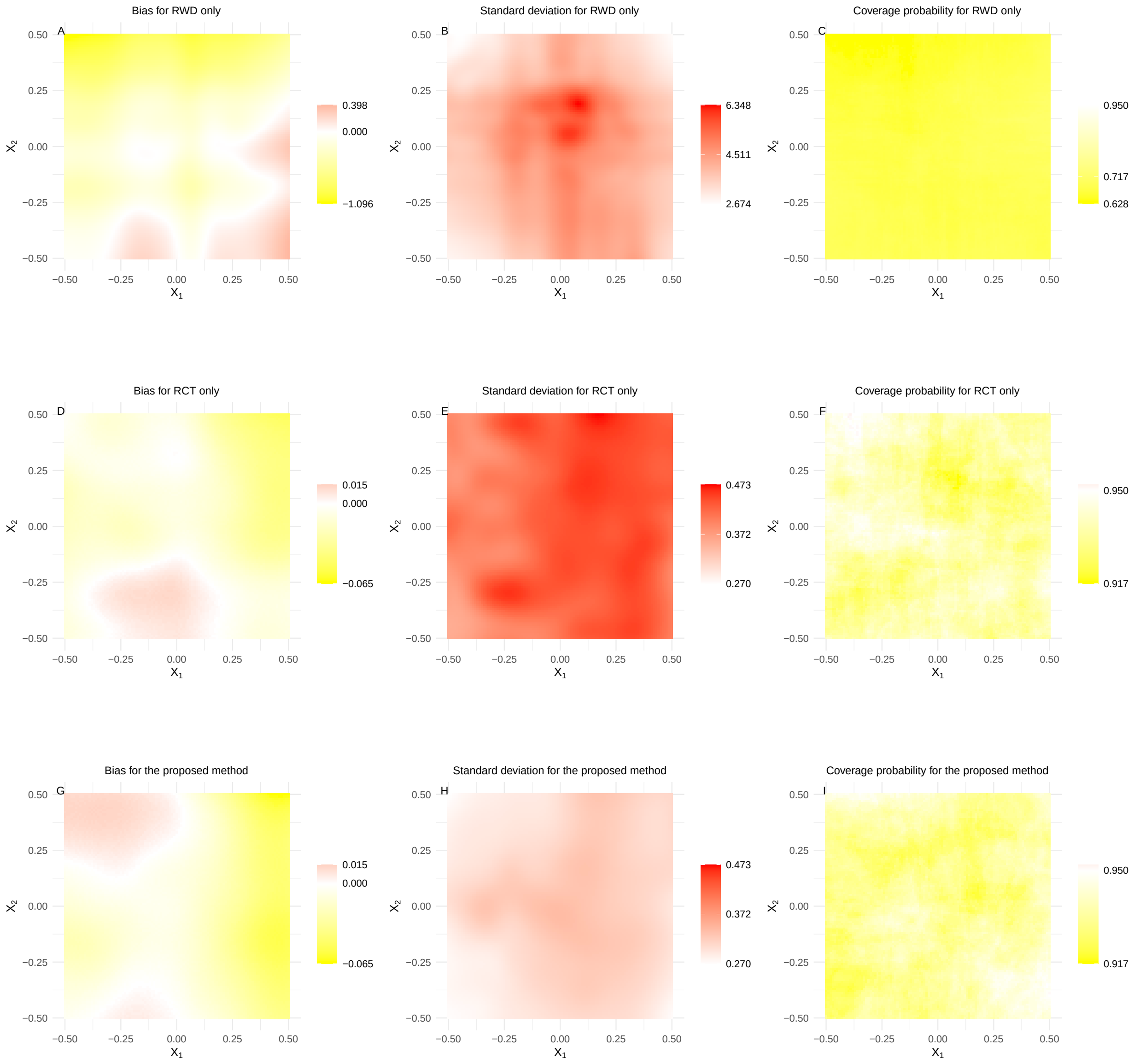

Figure S15. The simulation results of Case S2 with $(n_1, n_0) = (500, 1000)$, $X_3 = 1$, and $X_4 = 0$.

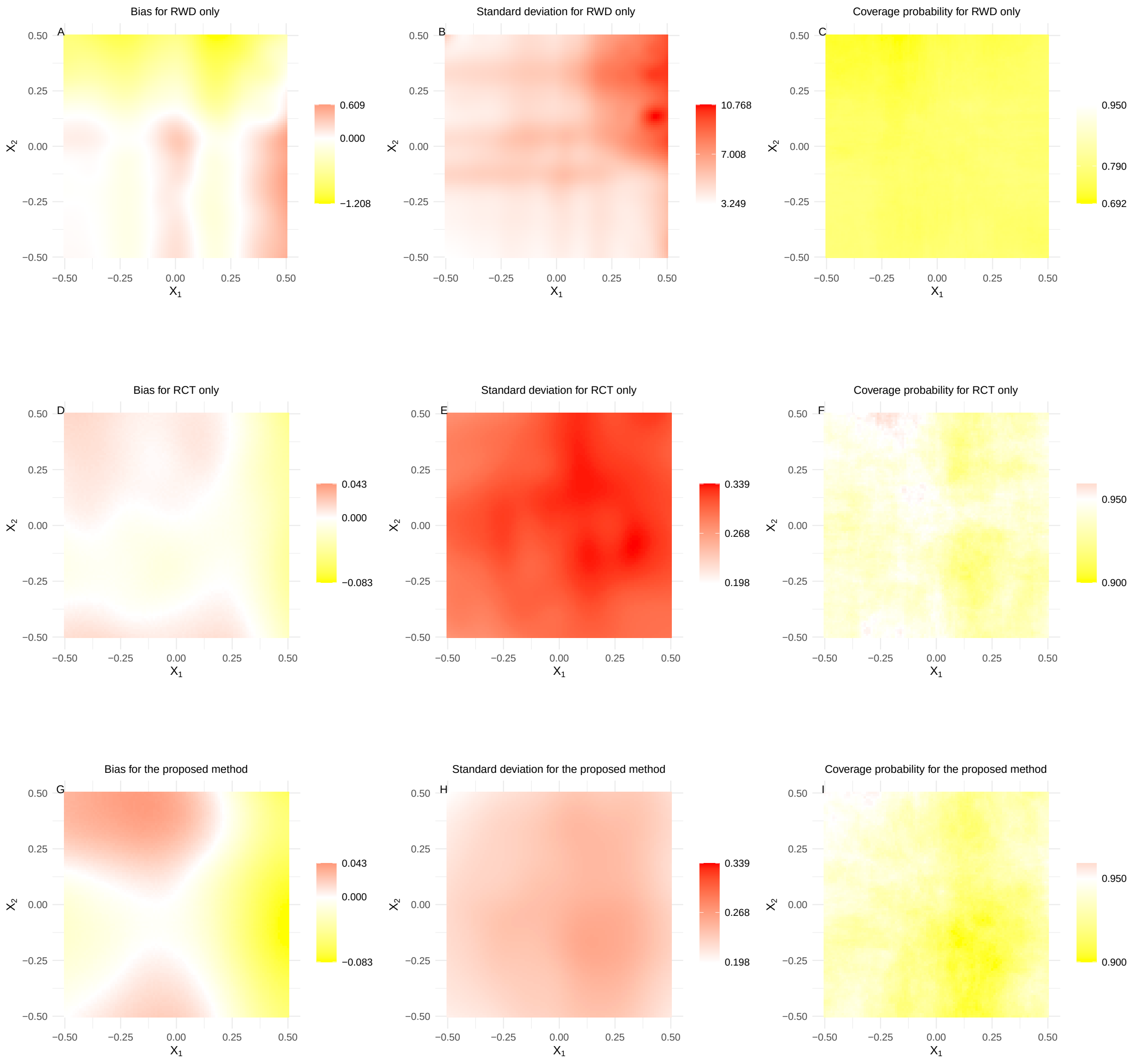

Figure S16. The simulation results of Case S2 with $(n_1, n_0) = (1000, 2000)$, $X_3 = 1$, and $X_4 = 0$.

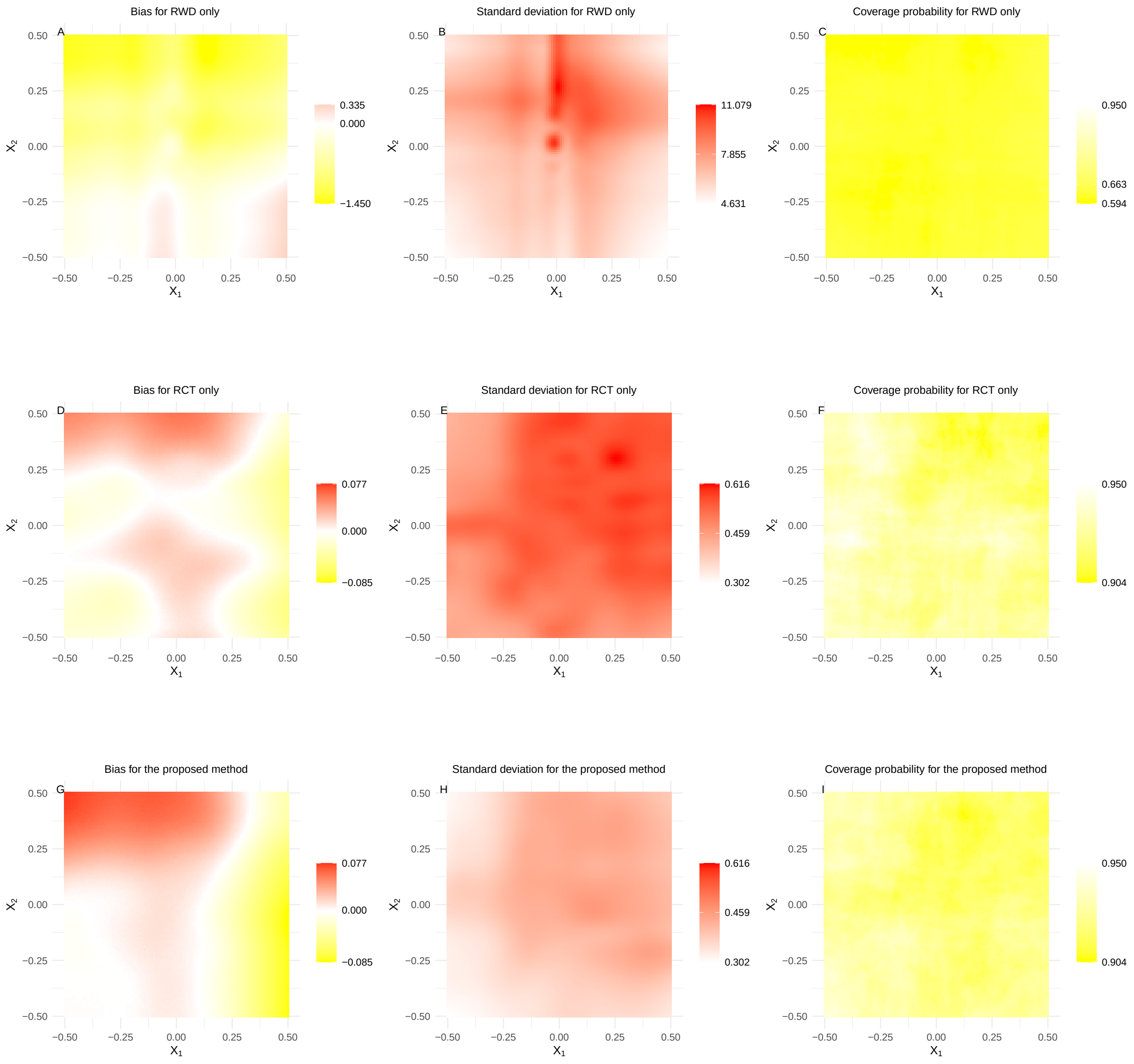

Figure S17. The simulation results of Case S2 with $(n_1, n_0) = (500, 1000)$, $X_3 = 0$, and $X_4 = 1$.

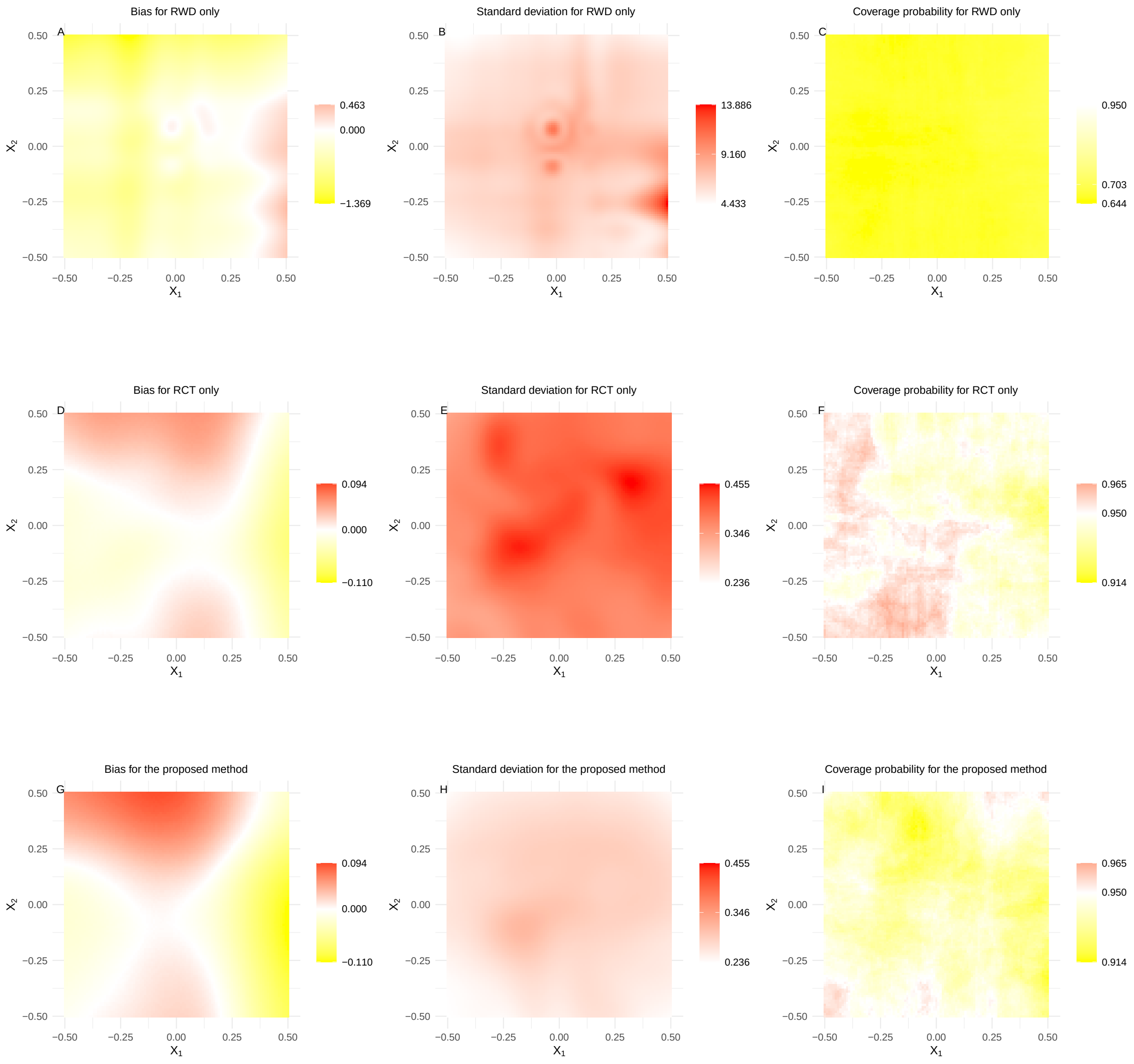

Figure S18. The simulation results of Case S2 with $(n_1, n_0) = (1000, 2000)$, $X_3 = 0$, and $X_4 = 1$.

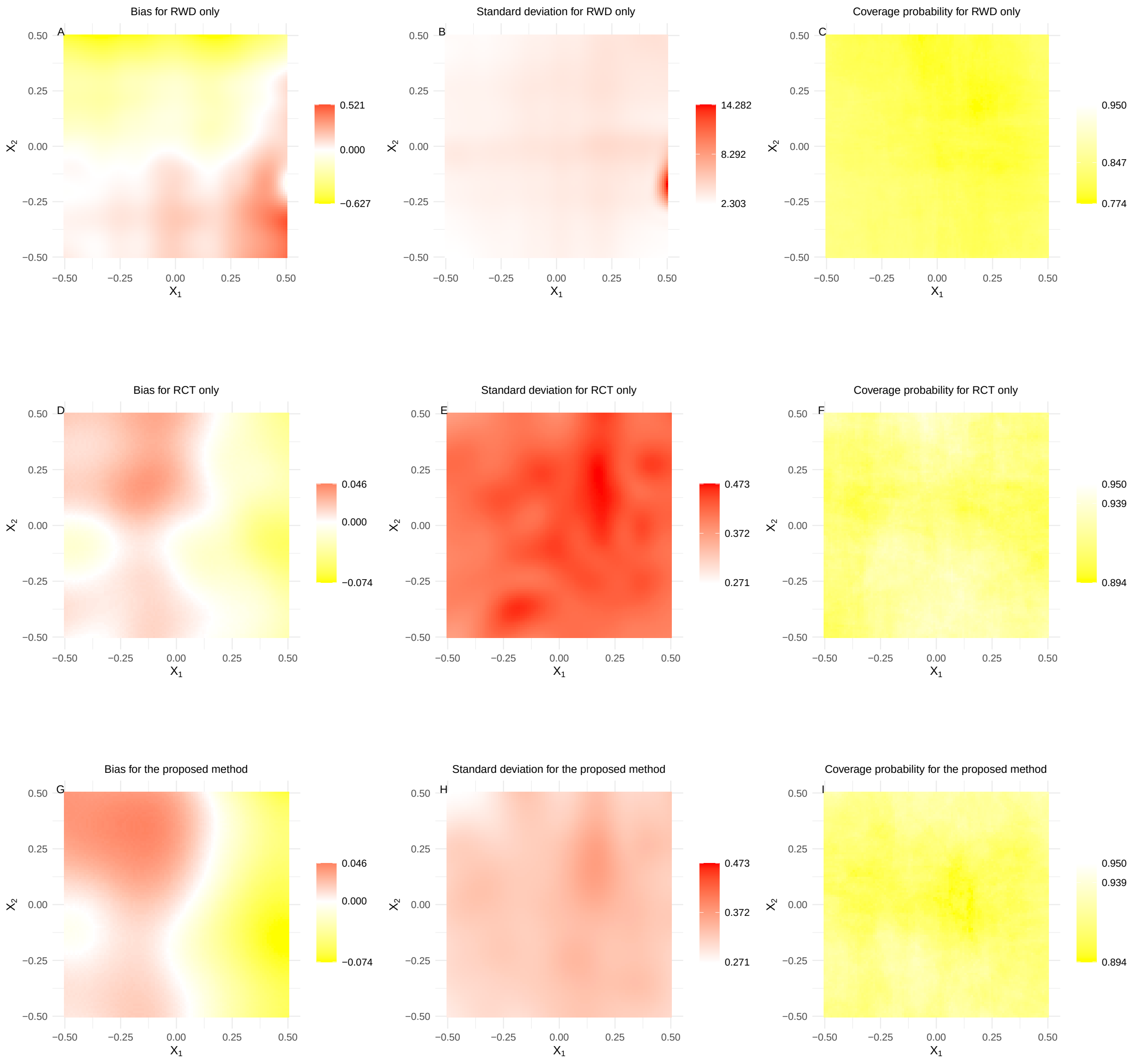

Figure S19. The simulation results of Case S2 with $(n_1, n_0) = (500, 1000)$, $X_3 = 1$, and $X_4 = 1$.

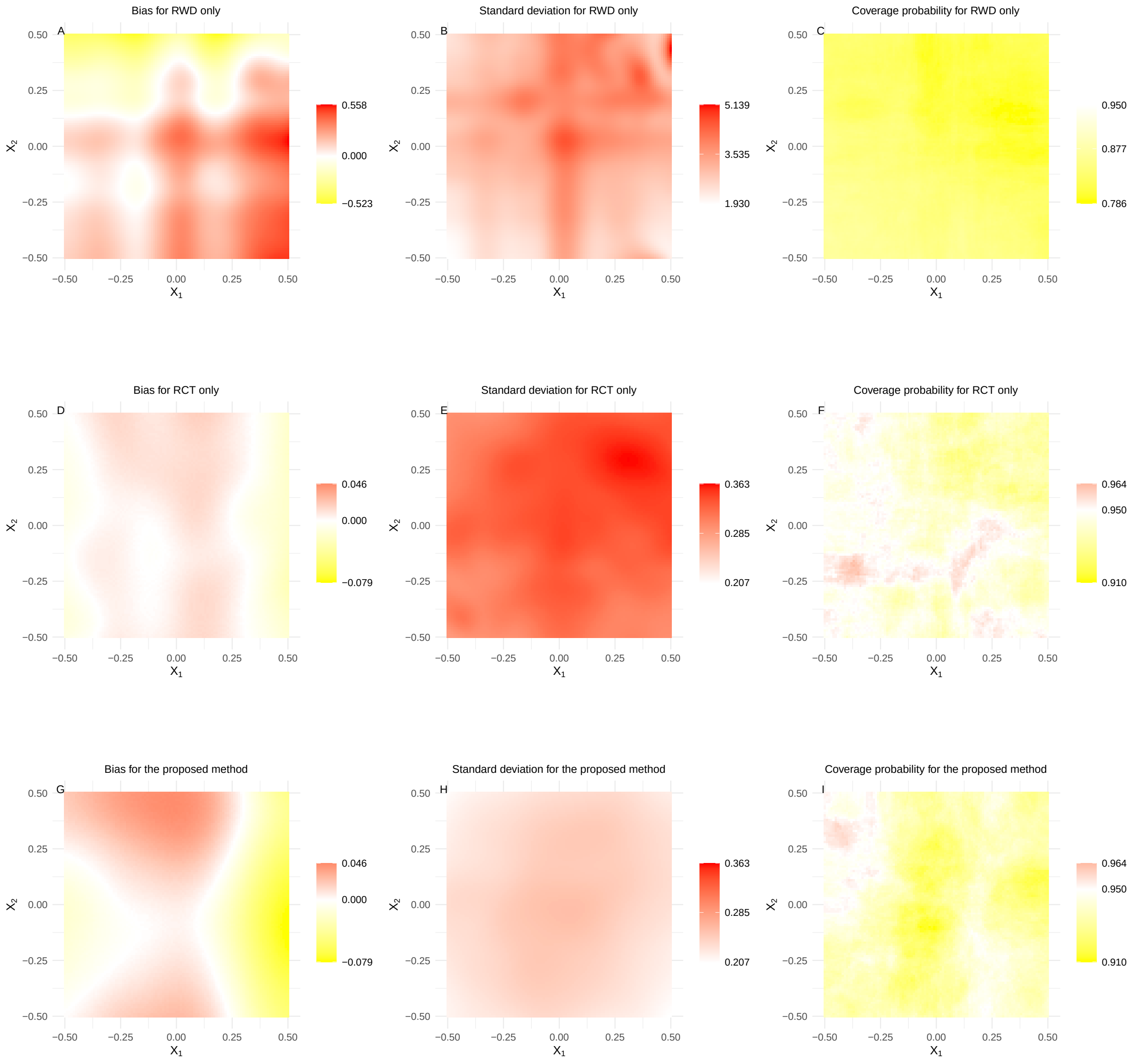

Figure S20. The simulation results of Case S2 with $(n_1, n_0) = (1000, 2000)$, $X_3 = 1$, and $X_4 = 1$.

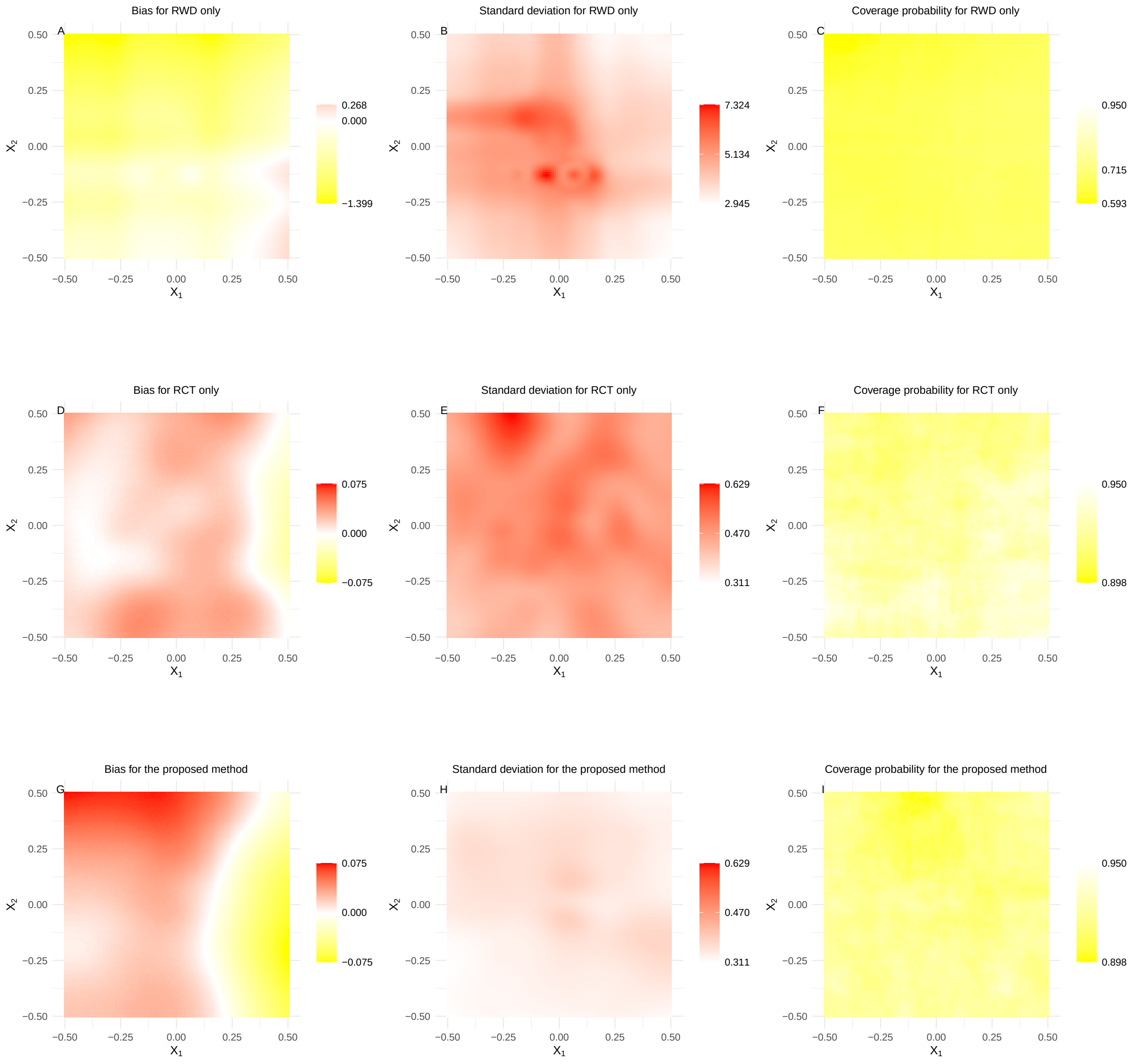

Figure S21. The simulation results of Case S3 with $(n_1, n_0) = (500, 1000)$, $X_3 = 0$, and $X_4 = 0$.

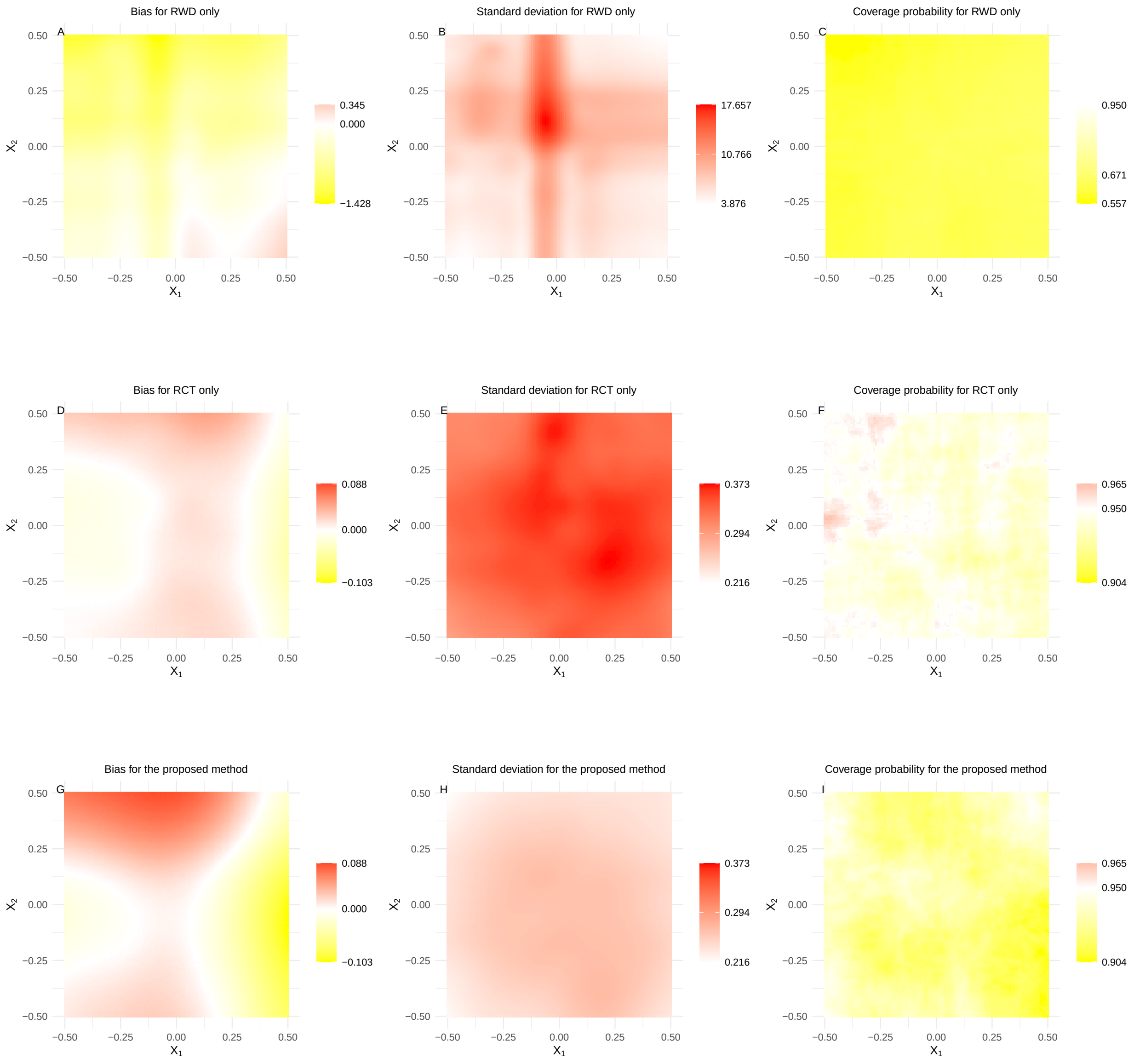

Figure S22. The simulation results of Case S3 with $(n_1, n_0) = (1000, 2000)$, $X_3 = 0$, and $X_4 = 0$.

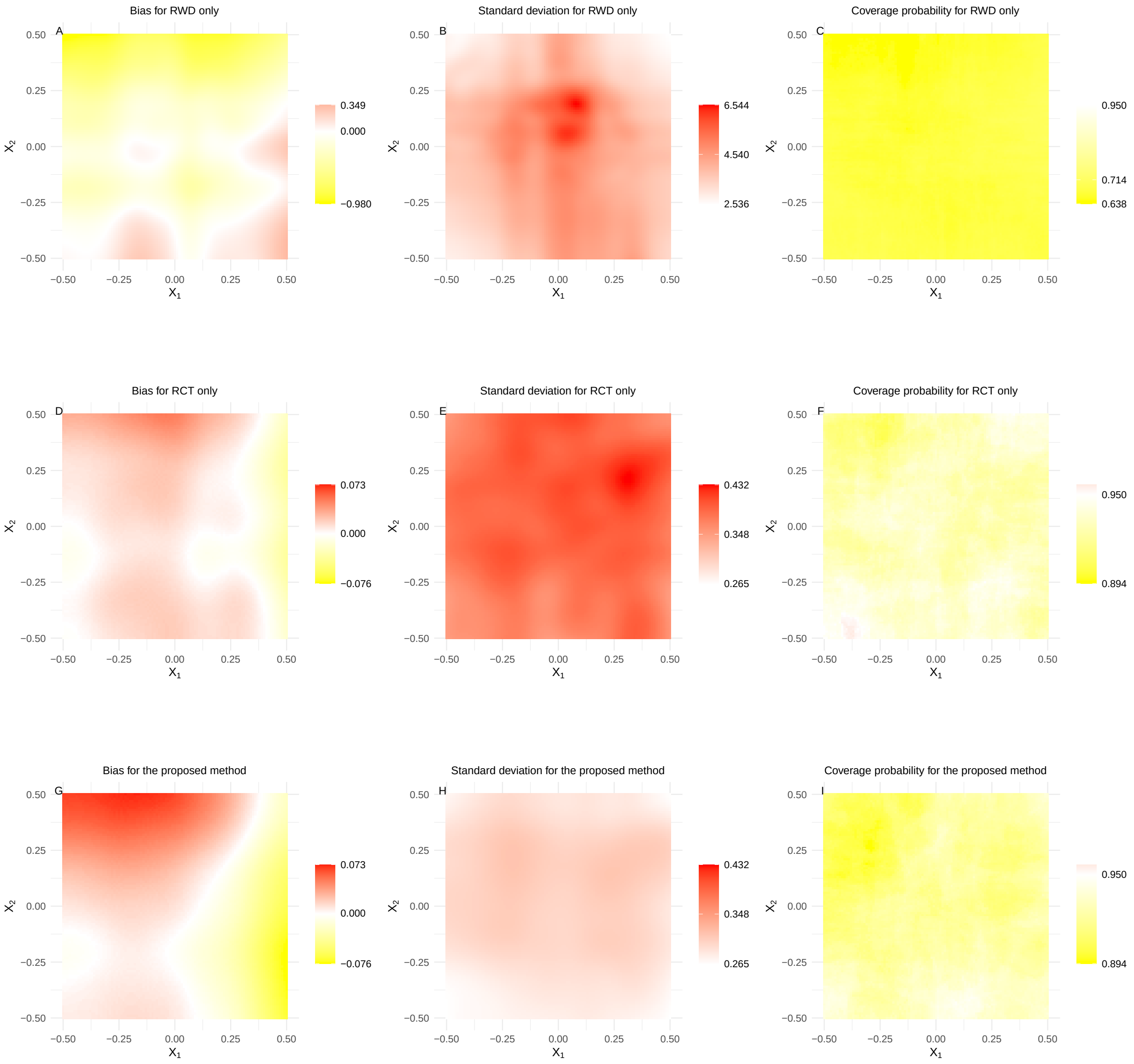

Figure S23. The simulation results of Case S3 with $(n_1, n_0) = (500, 1000)$, $X_3 = 1$, and $X_4 = 0$.

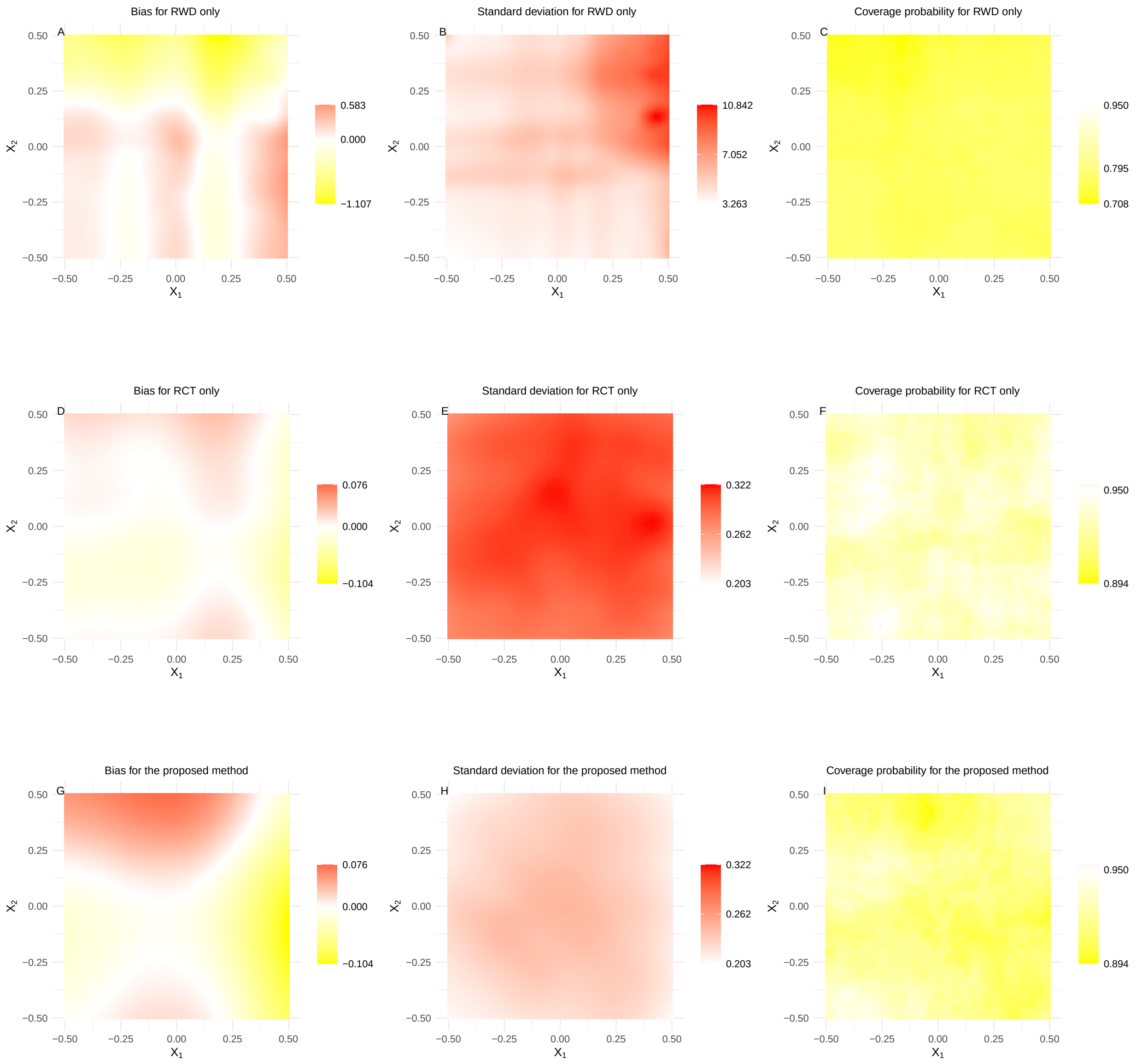

Figure S24. The simulation results of Case S3 with $(n_1, n_0) = (1000, 2000)$, $X_3 = 1$, and $X_4 = 0$.

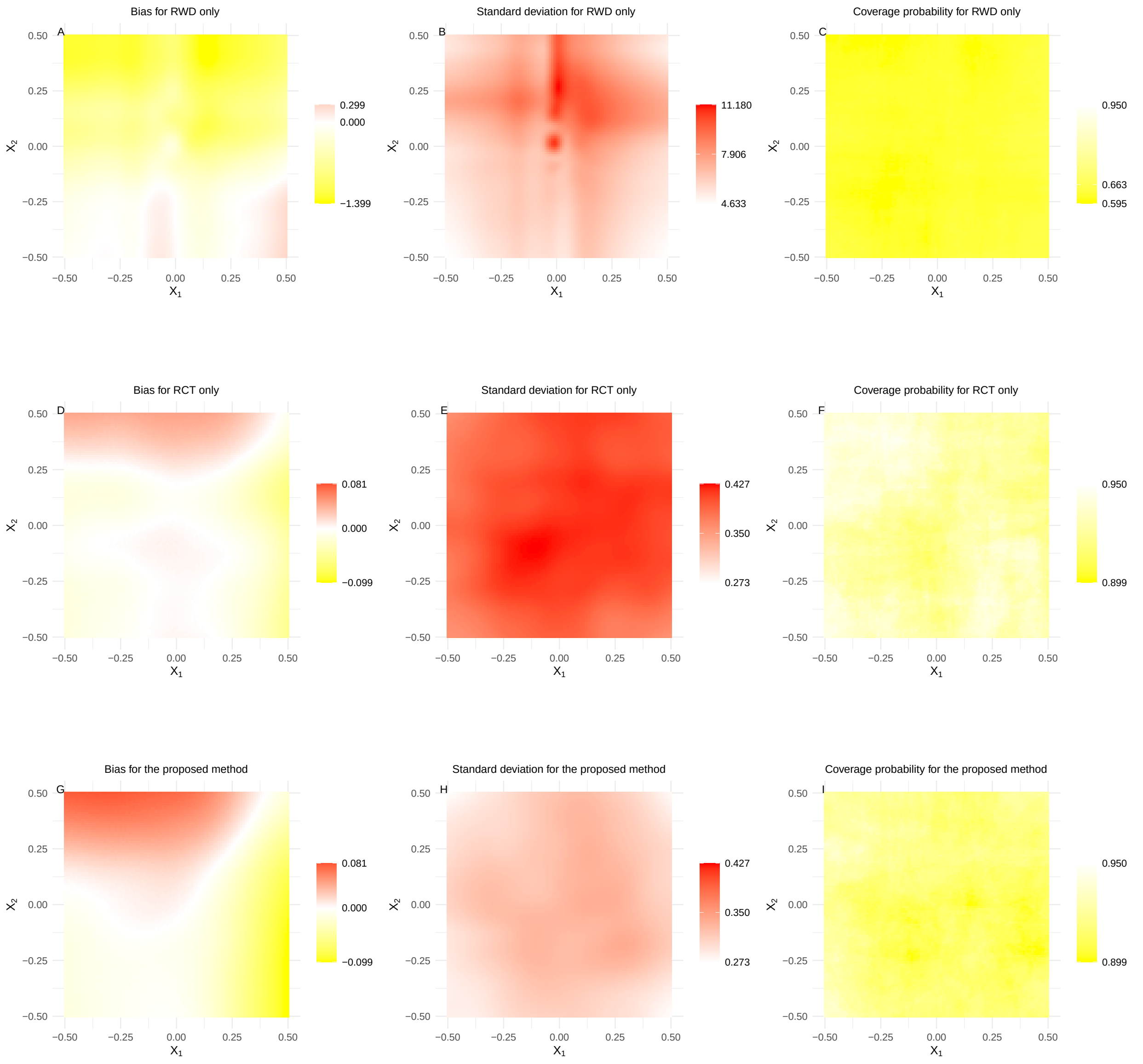

Figure S25. The simulation results of Case S3 with $(n_1, n_0) = (500, 1000)$, $X_3 = 0$, and $X_4 = 1$.

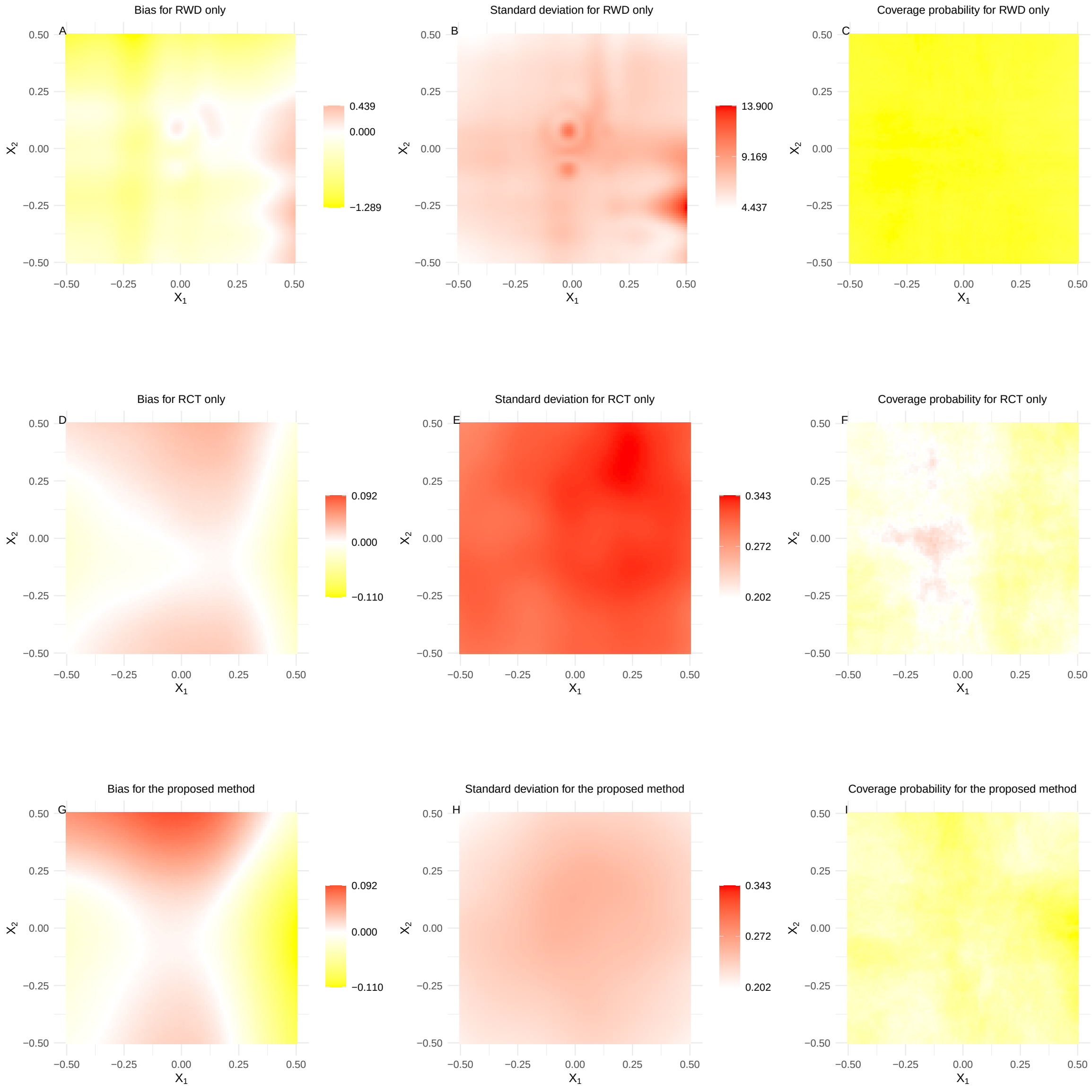

Figure S26. The simulation results of Case S3 with $(n_1, n_0) = (1000, 2000)$, $X_3 = 0$, and $X_4 = 1$.

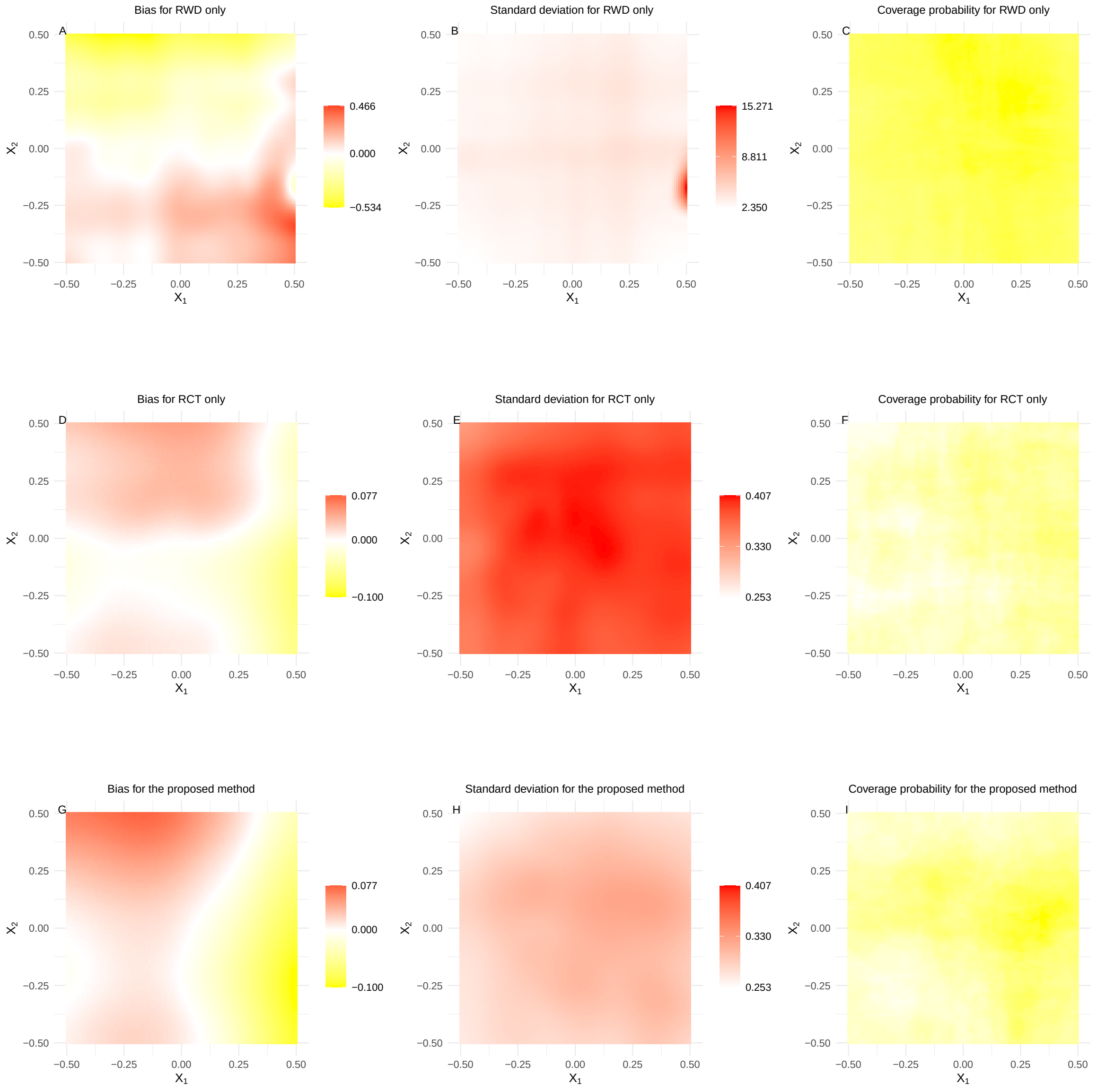

Figure S27. The simulation results of Case S3 with $(n_1, n_0) = (500, 1000)$, $X_3 = 1$, and $X_4 = 1$.

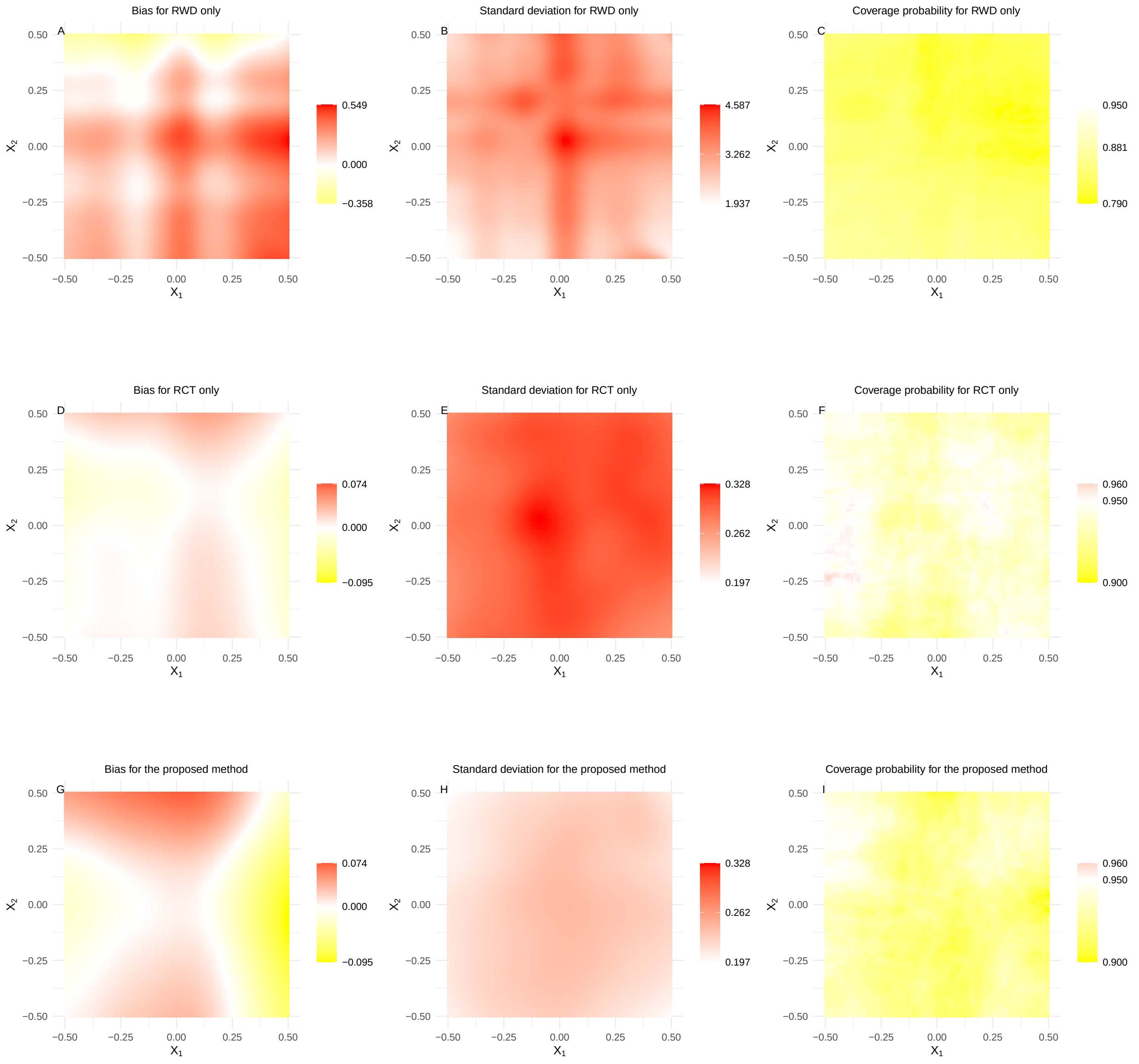

Figure S28. The simulation results of Case S3 with $(n_1, n_0) = (1000, 2000)$, $X_3 = 1$, and $X_4 = 1$.
